# Natural Algaecide Sphingosines Identified in Hybrid Straw Decomposition Driven by White‐Rot Fungi

**DOI:** 10.1002/advs.202300569

**Published:** 2023-07-03

**Authors:** Jing Hu, Effiong Kokoette, Caicai Xu, Shitao Huang, Tao Tang, Yiyi Zhang, Muyuan Liu, Yuzhou Huang, Shumiao Yu, Jie Zhu, Marianne Holmer, Xi Xiao

**Affiliations:** ^1^ Ocean College Zhejiang University #1 Zheda Road Zhoushan Zhejiang 316021 China; ^2^ Key Laboratory of Marine Ecological Monitoring and Restoration Technologies of Ministry of Natural Resources Shanghai 201206 China; ^3^ Key Laboratory of Watershed Non‐point Source Pollution Control and Water Eco‐security of Ministry of Water Resources College of Environmental and Resources Sciences Zhejiang University Hangzhou Zhejiang 310058 China; ^4^ Department of Biology University of Southern Denmark Odense 5230 Denmark

**Keywords:** allelochemicals, harmful algal blooms, metabolome, sphingosines, transcriptome

## Abstract

Harmful algal blooms (HABs), which are promoted by eutrophication and intensified by global warming, occur worldwide. Allelochemicals, which are natural chemicals derived from plants or microbes, are emerging weapons to eliminate these blooms. However, the cost and technical challenges have limited the discovery of novel antialgal allelochemicals. Herein, the decomposition of agricultural straws is manipulated by white‐rot fungi and achieved elevated antialgal efficiency. The transcriptomic analysis reveals that nutrient limitation activated fungal decomposition. By using a comparative nontarget metabolomics approach, a new type of allelochemical sphingosines (including sphinganine, phytosphingosine, sphingosine, and *N*‐acetylsphingosine) is identified. These novel natural algaecides exhibit superior antialgal capability, with as high as an order of magnitude lower effective concentration on blooming species than other prevalent allelochemicals. The co‐expression relationship between transcriptomic and metabolomic results indicate that sphinganine is strongly correlated with the differentially expressed lignocellulose degradation unigenes. The algal growth suppression is triggered by the activation of programmed cell death, malfunction of algal photosystem and antioxidant system, the disruption on CO_2_ assimilation and light absorption. The sphingosines reported here are a new category of allelochemicals in addition to the well‐known antialgal natural chemicals, which are potential species‐specific agents for HABs control identified by multi‐omics methodology.

## Graphical abstract

Using the enhanced degradation of white‐rot fungi, a significantly elevated antialgal property is achieved on agricultural straw, a novel natural algaecide, sphingosines are identified. These species–specific chemicals show superior antialgal capability against a series of typical blooming species. The inhibitory mechanism of a representative compound, sphinganine (DHS), lies in the interference of a series of physiological activities and gene expressions.

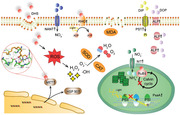

## 1 Introduction

Microalgae, as important primary producers in aquatic systems, contributes to half of the carbon fixed by photosynthesis on earth.^[^ ^1^ ^]^ In recent decades, harmful algal blooms (HABs) driven by eutrophication have been exacerbated by global warming,^[^ ^2^, ^3^, ^4^, ^5^, ^6^ ^]^ posing great threat on the sustainability of aquatic ecosystems and human society.^[^ ^7^ ^]^ Allelochemicals produced by plants, such as agricultural straws, or microbes have been suggested as a promising weapon to mitigate HABs. This is attributed to their superior species‐specific inhibitory abilities on bloom‐forming microalgae and high biodegradability, which exempts them from the risk of bioaccumulation.^[^ ^8^ ^–^ ^9^ ^]^

Using agricultural straw, which is rich in allelochemicals, has been a well‐known and worldwide practice in HABs prevention.^[^ ^10^ ^]^ This phenomenon was accidentally discovered in 1980 when adding rotting hay reduced filamentous microalgae growth in a British lake.^[^ ^11^ ^]^ Phenolic compounds derived from the degraded straw leachate have been suggested as phytotoxic chemicals that reduced the cyanobacterial and phytoplankton activity.^[^ ^12^, ^13^ ^]^ However, there is limited knowledge on the components of straw exudates and how to stabilize the antialgal effects of the straw.^[^ ^14^ ^]^ This information is needed, as 23% of the 268 documented applications failed in mitigating blooms.^[^ ^10^ ^]^ Only recently, white‐rot fungi (WRF) have been reported to facilitate the decomposition of barley straw and accelerate the progress of algal inhibition.^[^ ^15^ ^]^ Microbial activities involved in lignin degradation and the release of inhibitory chemicals were suggested to be responsible for the enhanced inhibitory capacity.^[^ ^15^ ^]^ However, it is still largely unknown how the WRF activities affect changes in the chemical composition during the decomposition and which chemicals play a role in enhancing the antialgal ability.

The limited knowledge of the WRF‐mediated straw system may largely be attributed to the complexity of the metabolites produced in the process of fungi activities and straw decomposition. To investigate the enhancement mechanism of WRF decomposition on straw’s antialgal properties, we applied a multi‐omics approach to unveil the transcriptomic and metabolomic profiles during the decomposition process. Through comparative nontarget metabolomics analysis on WRF‐mediated profiles, we discovered a novel type of allelochemical, sphingosines, which include sphinganine (DHS), phytosphingosine (PHS), sphingosine (SPH), and *N*‐acetylsphingosine (APH). These chemicals exhibit extremely low IC_50_ concentrations (half‐maximal inhibitory concentration), which are an order of magnitude lower than other well‐known antialgal chemicals. We have shown that they efficiently control the growth of six typical HAB‐forming species while remaining harmless to commercially beneficial algae, such as *Chlorella*. The mechanisms underlying algal inhibition include the activation of programmed cell death, dysfunction of the photosynthetic and antioxidation system, and the disturbance of several physiological/biochemical processes. Molecular docking visualized that ribulose‐1,5‐bisphosphate carboxylase/oxygenase II (*RuB*), rhodopsin (*RHO*), and metacaspase (*MET*) homology modeling proteins provided abundant sites for binding with sphinganine. Overall, we discovered a novel and highly efficient category of allelochemicals—sphingosines—through thorough investigation on the WRF‐enhanced antialgal capacity of agricultural straw, and uncovered their antialgal mechanism and species‐specific antialgal abilities against bloom‐forming species.

## 2 Results

### 2.1 WRF Enhances the Antialgal Capability of Agricultural Straw

**Figure 1:**
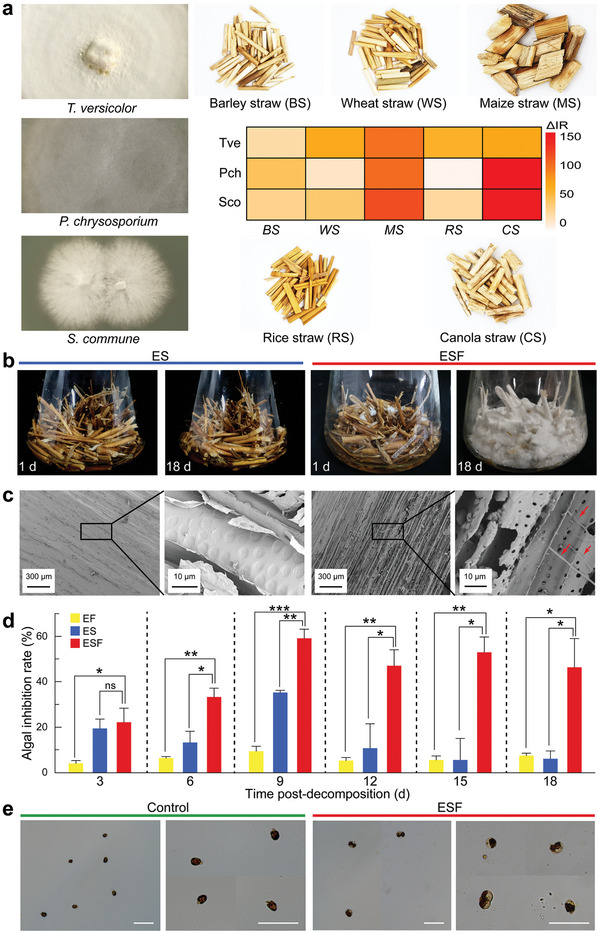
Agricultural straws degraded by white rot fungi (WRF) lead to enhanced algistatic ability. a) Enhancement of the antialgal capability (ΔIR) of agricultural straw decomposed by three species of WRF: *Trametes versicolor* (Tve), *Phanerochaete chrysosporium* (Pch), *Schizophyllum commune* (Sco). The exudates obtained from the straw without decomposition were taken as the background inhibitory level. The bioassays were performed on exponential *A. carterae* with an initial density of 5.0 × 10^4^ cells mL^−1^. b) Canola straw submerged in sterile water (ES, left) and inoculated with *T. versicolor* (ESF, right), the time stamps show the days after submersion or inoculation. c) Scanning electron micrographs of straws from ES and ESF groups on 18 days, showing the presence and penetration of hyphae on vessel walls (arrowheads) in (b). d) Increase in the algal inhibition rates of ESF, with comparison to the algal inhibition rates of ES and EF (*T. versicolor* exudates). e) Bright field micrographs of *A. carterae* morphological changes after 9 days exposure to ESF (right) compared to normal cells (left), the captured items are representatives of 100 cells observed in each treatment. Scale bar, 50 µm. a–e) All experiments were performed in three biological replicates and two technical replicates.

In general, the inhibitory effect of barley straw in the field has been reported to occur after several months of natural decomposition,^[^ ^16^, ^17^ ^]^ and previous studies on the antialgal enhancement of white rot fungi have focused only on barley straw.^[^ ^15^ ^]^ Therefore, we selected three common WRF species, namely *Trametes versicolor*, *Phanerochaete chrysosporium*, and *Schizophyllum commune*, to degrade five common agricultural straws, including barley (*Hordeum vulgare*), wheat (*Triticum aestivum*), maize (*Zea mays*), rice (*Oryza sativa*), and canola (*Brassica napus*) straws for 90 days. The growth inhibition of a worldwide toxic bloom‐forming species, *Amphidinium carterae*, by the degraded straw extract varied, with the algal inhibition rate ranging from 3% to 159% (**Figure** **1**a).

To gain insight into the WRF enhanced antialgal property, we chose canola straw and *T. versicolor* to decompose for 18 days for further investigation. It was observed that the mycelia pellicle inoculated on canola straw started extending slender hyphae after 24 hours, and a thick fungal mat was formed to colonize the straw after 18 days (Figure 1b). Scanning electron micrographs indicated the attack of fungal hyphae on straw vessel walls (Figure 1c). We then tested the straw extracts with (ESF) and without (ES) white‐rot fungi decomposition, as well as the exudates of *T. versicolor* (EF) on *A. carterae*. The antialgal property of ESF significantly exceeded ES after 6 days, and the antialgal ability of EF remained as low as 4–9% over the entire experiment (Figure 1d). Furthermore, we observed that ESF altered the morphology of algal cells. The general intact and oval algal cells were swollen and nearly circular, and the intracellular structure became granular and transparent (Figure 1e). Overall, our results demonstrated that the antialgal capability of agricultural straws was significantly improved by WRF, and the enhancement was mostly derived from the microbial straw decomposition.

### 2.2 Transcriptomic Profile of *T. versicolor* during Canola Straw Decomposition

**Figure 2:**
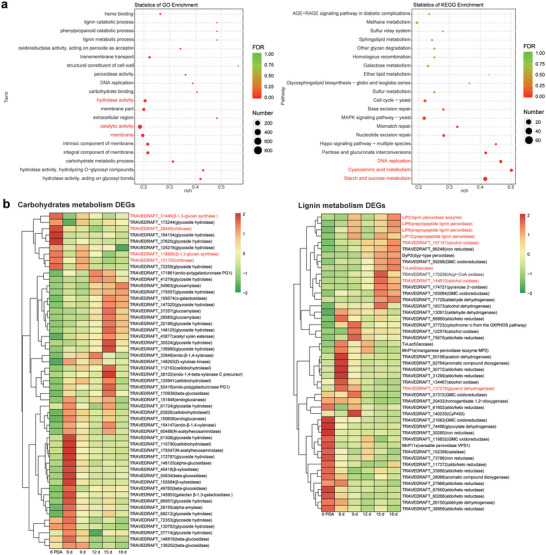
Nutrient deficiency is a prerequisite for lignocellulose degradation in *T. versicolor*. a) Top 20 terms enriched in GO (left) and pathways enriched in KEGG (right). The enriched terms or pathways of GO and KEGG were following the criterion of the adjusted *p*‐value (FDR). b) Expression level of the DEGs involved in the carbohydrate metabolism (left) and lignin metabolism (right). The DEGs presented in the heatmap were differentially expressed in pairwise comparison between 6, 9, 12, 15, 18 days straw‐cloned *T. versicolor* and 6 days fungi grown on potato dextrose agar (6 PDA). NCBI referenced gene symbol and description was given for DEGs. Go terms, KEGG pathways and gene names in red indicated specific discussed in the text.

Accordingly, we performed the *de novo* transcriptome assembly on the sequenced library to investigate the *T. versicolor* transcriptomic profile along with the decomposition progress. Particularly, we assembled a total of 33 437 unigenes in *T. versicolor*, which were further annotated by the Gene Ontology (GO) and the Kyoto Encyclopedia of Genes and Genomes (KEGG) database (Figure S1, Supporting Information). We next screened for the enriched pathways and differentially expressed unigenes (DEGs) during the decomposition. Notably, we identified 4031 DEGs (1781 upregulated and 2250 downregulated) between cultured *T. versicolor* and *T. versicolor* grown on straw. GO analysis showed that the DEGs were enriched in terms of catalytic activity, membrane, and hydrolase activity (**Figure** **2**a). In addition, the identified DEGs in pathways such as starch and sucrose metabolism, cyanoamino acid metabolism, and DNA replication were enriched in the KEGG database (Figure 2a).

Considering the significance of polysaccharides in WRF proliferation and the lignin‐based barrier that impedes carbohydrate utilization, we conducted a further analysis of the DEGs encoding oxidoreductases, carbohydrate‐active enzymes, and peroxidases (PODs) that are involved in lignocellulose degradation. As a result, we observed an upregulation of an array of unigenes encoding glycoside hydrolases on the 6th day, while unigenes involved in the synthesis of cell walls such as *β*‐1,3‐glucan synthase and chitinase were suppressed (Figure 2b). Following the 6th day, unigenes responsible for cell wall synthesis were activated, indicating the beginning of fungal exponential growth after a transient lag phase. Additionally, we found that the unigenes related to the secondary metabolism, specifically lignin peroxidase, manganese peroxidase, and laccase, were not expressed until the fungi were inoculated on the straw. The expression of these ligninolytic enzymes was consistent with auxiliary enzymes like alcohol oxidase and glycerol dehydrogenase (Figure 2b), which have been reported to generate reactive and nonspecific free radicals to increase the substrate accessibility.^[^ ^18^ ^]^ The reducing sugar concentration of ESF increased from 82.59 mg L^−1^ on the 3rd day to 747.92 mg L^−1^ on the 6th day, which coincides with the expression of carbohydrate‐active enzymes, and subsequently declined to 400 mg L^−1^ (Table S1, Supporting Information).

Together, this indicates that the nutrient limitation at the beginning of inoculation triggered the lignin decomposition by *T. versicolor*, and the co‐expression of accessory and ligninolytic enzymes contributes to the depolymerization of lignocellulose, resulting in changes to the dynamics of bioactive compounds in straw extracts and, consequently, variations in the antialgal property between ESF and ES. This raises the question of how the chemicals derived from WRF‐rotted straw change during decomposition.

### 2.3 Toward a Molecular Understanding of *T. versicolor* Driven Decomposition of Canola Straw

**Figure 3:**
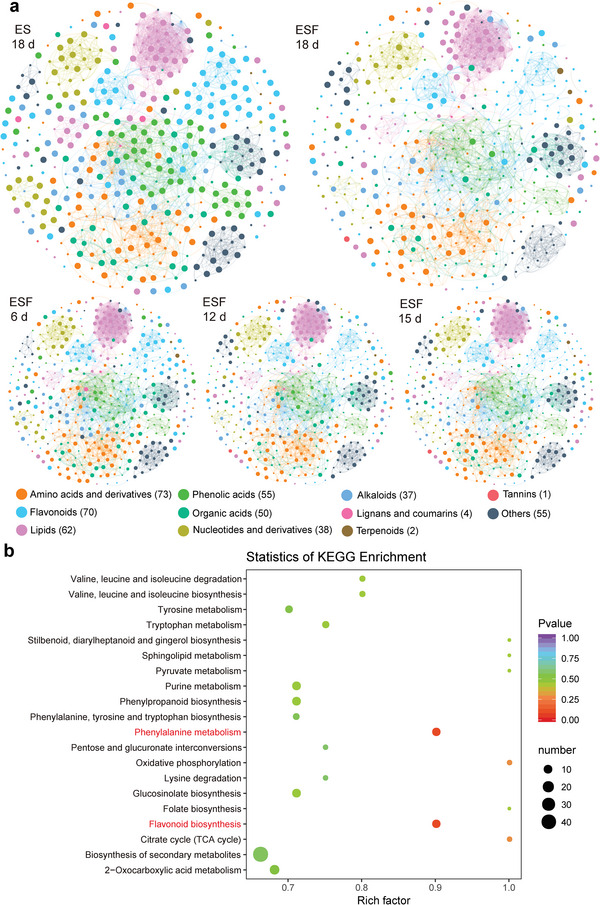
WRF decomposition elicits the alteration of straw extract attributes. a) The metabolite molecular network was presented with nodes colored by the eleven major metabolite clusters, each node represents an identified metabolite. The interlink between nodes was based on the structural similarity of metabolites with a Tanimoto index ≥ 0.75, the size of each node is proportional to the ion abundance of each metabolite in UPLC‐MS/MS analysis. b) KEGG enrichment of the differentially regulated metabolites between 18 days’ ES and ESF. Totally, 20 KEGG pathways with the top hypergeometric *p*‐value were displayed, pathways in red indicated specific addressed in the text. The data was shown the mean of three biological replicates.

Agricultural straws are a prolific source of natural compounds with intricate chemical structures and potent bioactivities, including polyphenolics, fatty acids, amino acids and their derivatives, which could contribute to the antialgal property of straw.^[^ ^19^ ^]^ Therefore, we analyzed canola straw extracts using UPLC‐MS/MS. Metabolite molecular network is a powerful tool for identifying analogs of natural products, exploring underlying bioactive molecules, identifying key metabolites and pathways involved in disease, developing novel medicines, and promoting the understanding of the chemistry of ecological interactions.^[^ ^20^, ^21^, ^22^, ^23^ ^]^ Thus, to further investigate the spectrometric dynamics of metabolites driven by the white rot fungi, we used Tanimoto coefficient to cluster the structural similar molecules identified in the straw extract.^[^ ^24^ ^]^ As a result, we identified 11 well‐defined classes and a total of 447 metabolites using UPLC‐MS/MS (**Figure** **3**a, Table S2, Supporting Information). The molecular network revealed that phenolic acids were the most abundant metabolites and decreased during the decomposition, followed by organic acids, nucleotides and their derivatives. Principal component analysis indicated a clear separation between ESF and ES, suggesting that the metabolite profiles with and without fungi are distinguishable (Figure S2, Supporting Information). More specifically, we observed significant regulation of 306 metabolites, with 221 downregulated and 85 upregulated in the pairwise comparison (Table S3, Supporting Information). KEGG pathway analysis showed significant enrichment in the flavonoid biosynthesis and phenylalanine metabolism pathways (Figure 3b). The abundance of (+)‐gallocatechin, 7,4'‐dihydroxyflavone, (−)‐epicatechin, and (+)‐catechin was significantly upregulated in ESF in the flavonoid biosynthesis pathway, while hesperetin, chlorogenic acid, myricetin, *p*‐coumaroyl quinic acid, and kaempferol were significantly downregulated. Additionally, the concentration of most metabolites in the phenylalanine metabolism pathway, such as phenylacetylglycine, trans‐cinnamate, and 4‐hydroxyphenylacetate, was significantly downregulated, while l‐phenylalanine was significantly upregulated. These findings support the notion that WRF‐mediated microbial activities play a key role in modifying the metabolic fingerprint profile of canola straw, which contributes to its elevated antialgal capability.

### 2.4 Upregulated Metabolites Induce the Growth Arrest of *A. carterae* and Other Bloom‐Forming Species

**Figure 4:**
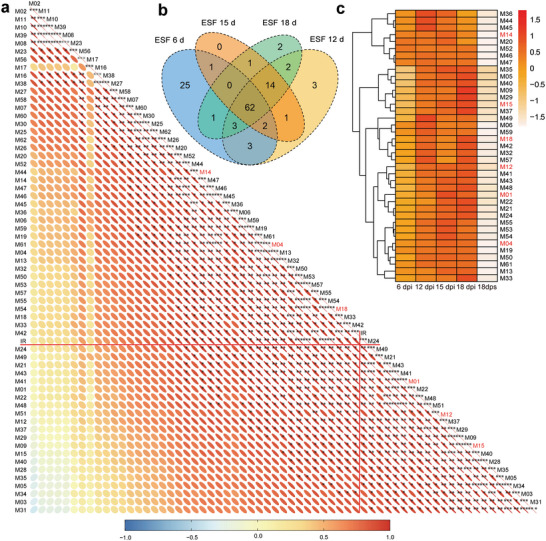
The relative content of upregulated metabolites (URMs) showed a significant correlation with algal inhibition rate in ESF. a) Pearson correlation coefficients between algal inhibition rate and metabolites’ relative content represented by ion abundance. The size of the node represents *p*‐value, whereas the direction of the node displays the positive (red) or negative (blue) of the correlation coefficients. Nodes represent the correlation result between metabolites and algal inhibition rate were labeled with red lines. b) Venn diagram showing the time‐course distribution of URMs among different comparison groups. All metabolites in four ESF groups (6 days, 12 days, 15 days, 18 days) were compared with the metabolites in 18 days’ ES, respectively. c) Heatmap depicts the relative ion abundance of positively correlated URMs among straw groups (*r* > 0.75, *p* < 0.05). The values were means of three replicates, “dpi” and “dps” stand for “days post inoculation” and “days post submersion”, respectively. The top 6 accessible metabolites tested in the validation work are in red. M1: l‐methionine, M4: choline alfoscerate, M12: sphinganine, M14: DL‐alanyl‐DL‐phenylalanine, M15: 3‐hydroxyanthranilic acid, M18: phytosphingosine.

**Figure 5:**
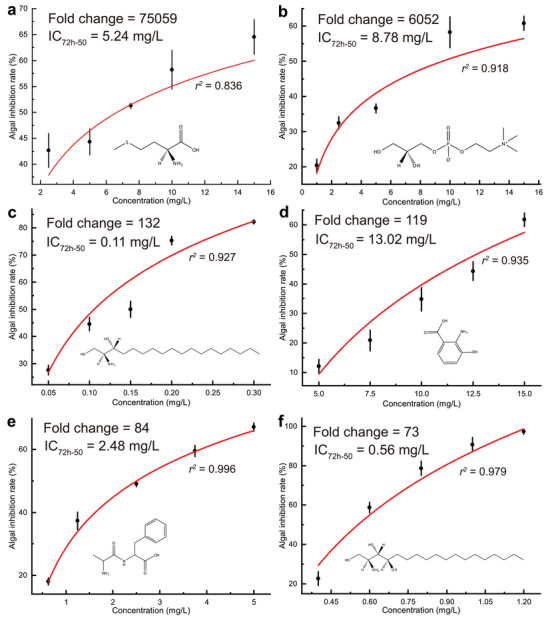
Inhibition regression lines of top six upregulated metabolites (URMs) in ESF. a) l‐methionine (M01), b) choline alfoscerate (M04), c) sphinganine (M12), d) 3‐hydroxyanthranilic acid (M15), e) DL‐alanyl‐DL‐phenylalanine (M14), f) phytosphingosine (M18). a–f) The inhibition test of pure chemicals was performed in a six‐well plate with an initial density of 5.0 ×10^4^ cells mL^−1^ (10 mL). The applied volume of each chemical was controlled in 1‰ (v/v), and the equivalent volume of the respective solvent was added as a control. The fold changes show the upregulated times of the same metabolite observed in 18 days’ ESF and ES. All experiments were performed in three biological replicates and each biological replicate was applied in duplicate.

Specifically, we identified 97, 90, 81, and 85 upregulated metabolites (URMs) in the pairwise comparison between ES and ESF on Day 6, 12, 15, and 18, respectively. Among these, we found a total of 62 URMs that were shared across all four time points (**Figure** **4**b). Next, we performed a correlation analysis between the abundance of these URMs and the antialgal capability, and identified 39 URMs from eight chemical categories that significantly correlated with the antialgal efficiency (Figure 4a). The hierarchical clustering showed that the concentration of these URMs increased during the mid‐later decomposition (Figure 4c). To investigate the enhanced antialgal property in ESF, we examined the antialgal ability of the URMs that showed a significant positive correlation with the largest fold changes, namely, l‐methionine, choline alfoscerate, sphinganine, 3‐hydroxyanthranilic acid, DL‐alanyl‐DL‐phenylalanine, and phytosphingosine. All these chemicals exhibited antialgal effects on *A. carterae*, with IC_50_ ranged from 0.11 to 13.02 mg L^−1^ (**Figure** **5**).

Notably, we observed that the DHS and PHS showed surprisingly strong antialgal capabilities against *A. carterae*, with low IC_50_ of 0.11 and 0.56 mg L^−1^, respectively (Figure 5e,f). Therefore, we tested more sphingosines against seven other algal species, including DHS and PHS identified from canola straw, and two additional chemicals, SPH and APH. Beyond our expectation, all the tested chemicals demonstrated extremely potent algal inhibitory efficiencies against bloom‐forming algae (**Table** **1**). Among these, DHS (IC_50_: 0.06–0.11 mg L^−1^), PHS (IC_50_: 0.00–0.56 mg L^−1^), and SPH (IC_50_: 0.12–0.26 mg L^−1^) showed greater antialgal activity than APH (IC_50_: 0.25–0.96 mg L^−1^). The species tested from the phylum of Dinoflagellata, Ochrophyta, Haptophyta, Bacillariophyta, and Cyanobacteria were all found to be vulnerable to sphingosines. Notably, *Chlorella* sp., a species with beneficial use in aquaculture, exhibited up to two orders of magnitude higher resistance to these chemicals than previous bloom‐forming species, with an IC_50_ of 0.49–0.67 mg L^−1^. There has been limited research conducted on the sensitivity of organisms at different trophic levels to sphingosines. Therefore, we conducted the quantitative structure‐activity relationships (QSAR) models to examined the development toxicity, mutagenicity, and ecotoxic effects of sphingosines on other nontarget organisms. The results showed that all tested chemicals were non‐toxic on development toxicity and exhibited negative mutagenicity (Table S4, Supporting Information). The 50% lethal or growth inhibition concentrations were much higher than their effective concentrations on HABs species. For instance, the QSAR model predicted the 48‐h *Tetrahymena pyriformis* 50% growth inhibition concentration of DHS as 1.66 mg L^−1^, the 48‐h *Daphnia magna* 50% lethal concentration as 4.65 mg L^−1^, the 96‐h *Fathead minnow* 50% lethal concentration as 0.79 mg L^−1^, and the oral rat lethal concentration as 8469.37 mg L^−1^.

**Table 1:** 72h‐IC_50_ (mg L^−1^) of target species treated with sphingosines

		DHS	PHS	SPH	APH
Target species and formula	Phylum	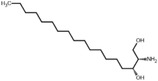	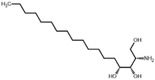	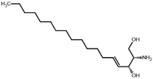	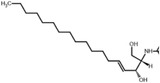
*Amphidinium carterae*	Dinoflagellata	0.112	0.561	0.257	0.961
*Heterosigma akashiwo*	Ochrophyta	0.108	0.033	0.117	0.248
*Phaeocystis globosa*	Haptophyta	0.062	0.030	0.134	0.961
*Isochrysis galbana*	Haptophyta	0.069	0.004	0.244	NT
*Skeletonema costatum*	Bacillariophyta	0.091	0.005	0.188	NT
*Microcystis aeruginosa FACHB‐905*	Cyanobacteria	0.094	0.014	0.129	NT
*Chlorella* sp.	Chlorophyta	0.674	0.468	0.628	NT

^a)^ NT, None test.

**Figure 6:**
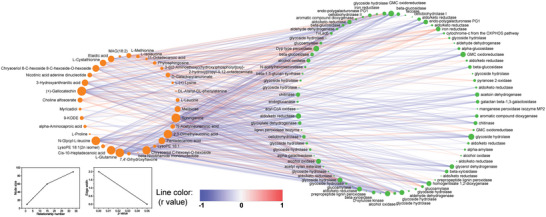
Co‐occurrence network between upregulated metabolites (URMs, left) and differentially expressed unigenes (DEGs, right) involved in lignocellulose degradation. The linear relationship was analyzed over 18 days using the Pearson correlation coefficient (*p* < 0.05), and the line color indicates the *r* value positive (red) or negative (blue). The node size represents the relationship number, and the edge width shows the *p* value.

Interestingly, the co‐occurrence network to elucidate the relationship between the shared URMs and differentially expressed unigenes showed that DHS is one of the most strongly correlated node among the shared URMs (**Figure** **6**). A positive correlation between DHS and the Dyp‐type peroxidase was detected, which is a newly discovered haem‐containing peroxidase degrading non‐phenolic lignin.^[^ ^25^ ^]^ The expression of Dyp‐type peroxidase increased 34 times after 18 days of decomposition, implying its important role in the upregulation of DHS and the elevated antialgal property of canola straw extract.

### 2.5 Modulation of DHS Prevents *A. carterae* Blooming

DHS is a free sphingoid bases that play vital structural, cellular, and regulatory roles in eukaryotic organism.^[^ ^26^, ^27^ ^]^ However, the suppression of DHS on algal growth has not been reported, and the mechanism remains unknown.

**Figure 7:**
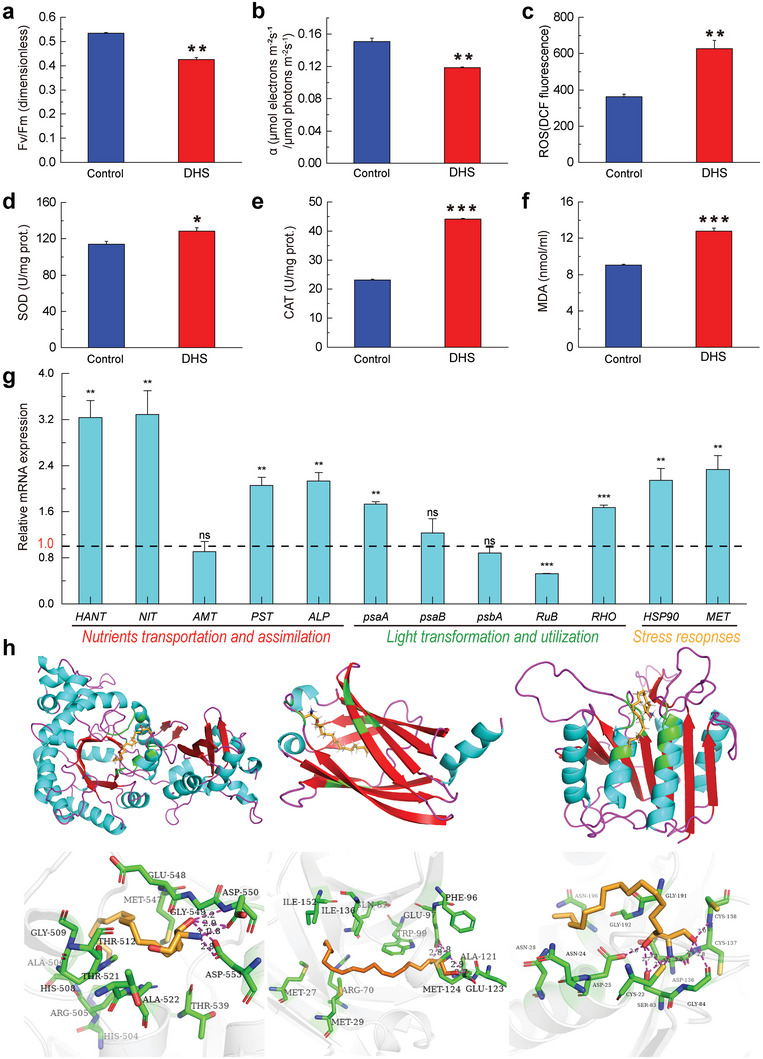
Physiological stress, transcriptional response, and structural complex docking of *A. carterae* induced by DHS. a,b) Photosynthetic parameters observed by PAM fluorometry on exposed algal cells. c) Intracellular ROS levels of exposed algal cells. d,e) Intracellular SOD and CAT activities of exposed algal cells. f) MDA content of exposed algal cells. g) Relative mRNA expression abundance of genes involved in photosynthesis activities, nutrients assimilation, and environmental stress defense. h) Homology modeling of *RuB* (left), *RHO* (middle), and *MET* (right) encoded proteins and their respective predominant interactions with DHS. Protein receptors are shown as ribbons, ligands and the key amino acid residues are shown as sticks, and hydrogen bonds are shown as magenta dashes. a–f) All experiments were performed in three independent experiments under 1‰ (v:v) DMSO or 0.13 mg L^−1^ DHS (120 h‐IC_50_), and algal initial density was 5 × 10^4^ cells mL^−1^.

We first investigated the effect of DHS on algal photosynthesis. After 5 days of exposure, the Fv/Fm and *α* values of the cells decreased by 20% and 21%, respectively (**Figure** **7**a,b), indicating disrupted algal photosynthesis by DHS. We also measured intracellular ROS, superoxide dismutase activity (SOD), catalase activity (CAT), and malondialdehyde content (MDA). Notably, DHS treatment increased the algal ROS level by 2‐fold (Figure 7c), which activated the enzymatic antioxidant activity of SOD and CAT by 13% (Figure 7d) and 91% (Figure 7e), respectively. Consistent with the ROS level and enzyme activity, the lipid peroxidation products MDA also increased by 41% (Figure 7f). These results demonstrated that DHS triggers oxidative stress in algae.

We then analyzed the relative expression of genes responsible for intracellular functions. Four transcripts encoding high‐affinity nitrate transporter (*HANT*), nitrite transporter (*NIT*), phosphate transporter (*PST*), and alkaline phosphatase (*ALP*) were upregulated for 3.24, 3.29, 2.06, and 2.13 times, respectively (Figure 7g). The expression of photosystem I (PSΙ) P700 chlorophyll a (*psaA*) and rhodopsin (*RHO*) was significantly upregulated, while no difference was found in the core protein of PSΙ processes (*psaB*) and photosystem II (PSII) protein D1 (*psbA*). In contrast, the transcript abundance of a key enzyme for CO_2_ assimilation, *RuB*, was prominently suppressed by 50%. Additionally, the expression abundance of heat shock protein 90 (*HSP90*) and metacaspase (*MET*) were both overexpressed. Thus, *A. carterae* genes involved in photosynthetic activities, energy, nutrients acquisition, and environmental stress resistance were disturbed following the exposure of DHS.

Furthermore, we conducted molecular docking on all significantly regulated genes' homology modeling proteins to reveal the potential interaction behavior between DHS and these receptors. The absolute value of forecasted binding energy indicated that *RuB*, *RHO*, and *MET* proteins were the most potential targets, explained by their superior binding affinity among all simulated proteins (**Table** **2**). For instance, the optimal binding model diagrammatically depicted five hydrogen bonds and twelve hydrophobic interactions in the DHS‐*RuB* system (Figure 7h). The amino/hydroxyl groups of DHS formed hydrogen bonds with the residues Asp550, and Asp553 of *RuB*. The residues His504, Arg505, Ala506, and others participated in the hydrophobic interaction (Table 2). As a result, the homology modeling proteins provided diverse binding sites to bond with DHS. This result suggested that proteins encoded by *RuB*, *RHO*, and *MET* might be representative targets for intracellular disruption. The upregulation of *RHO* and *MET* implies that a negative feedback loop was activated to compensate for the deactivation of these target proteins.

**Table 2:** Predicted binding sites and their binding energy (ΔG kcal/mol) simulated by molecular docking

Receptors	Δ*G*	Residues participate in hydrogen‐bonding interaction	Residues participate in hydrophobic interaction
*HANT*	−6.01	Asp315, Thr316	Ser176, Leu200, Phe203, Ile227, Gly228, Gly231, Asn237, Trp240, Cys305, Leu311, Leu312
*NIT*	−5.56	Val200, Leu202, Asn206	Leu131, Val134, Lys135, Cys195, Gly199, Glu201, Gly205, Val209
*ALP*	−5.59	Glu119, Ile120, Thr122	Cys121, Pro173, Asn174, Ser175, Ala232, Met233, Gly234, Val235, Trp279, Glu283, Glu303
*psaA*	−6.01	Pro103, Ser104, Ile105, Thr328	Ala106, Asn114, Ile115, Asn117, Ile124, Arg125, Val126, Asp329, Val667
*RuB*	−6.82	Asp550, Asp553	His504, Arg505, Ala506, His508, His509, Thr512, Thr521, Ala522, Thr539, Met547, Glu548, Gly549
*RHO*	−6.76	Glu97, Ala121	Met27, Met29, Val58, Arg70, Gln87, Phe96, Trp99, Glu123, Met124, Ile136, Ile152
*HSP90*	−5.81	Gly120, Gly199	Leu14, Met83, Thr100, Phe103, Phe119, Val121, Phe123, Tyr124
*MET*	−6.74	Cys22, Asp25, Ser83, Gly84, Asp136, Cys138	Asn24, Asn28, Cys137, Gly191, Gly192, Asn196

^a)^ The simulation result for *PST* was not available as the pocket of its homology modeling protein for docking was inapplicable.

## 3 Discussion

### 3.1 Algal Inhibitory Effect and Mechanism of Sphingosines

In this work, we identified a new category of antialgal allelochemical—sphingosines. Both DHS and PHS, which were identified in canola straw decomposition, and their analogs exhibit a superior antialgal ability against HABs formers compared with other prevailing allelochemicals. Taking the notorious *M. aeruginosa* as an example, 2‐phenyl‐phenol (IC_50_: 0.15 mg L^−1^)^[^ ^13^ ^]^ and berberine (IC_50_: 0.28 mg L^−1^)^[^ ^28^ ^]^ are the most pronounced allelochemicals from the category of polyphenolics and alkaloids. However, for the same algal species, allelochemicals from the category of PHS showed surprisingly low IC_50_ of 0.01 mg L^−1^ (Table 1). Moreover, for a typical red‐tide organism *H. akashiwo*, 0.5 mg L^−1^ of a novel allelochemical N*ω*‐acetylhistamine, secreted by the algicidal bacterium *Bacillus* sp. was reported to inhibit the algal growth by 50%,^[^ ^29^ ^]^ whereas the corresponding concentration for PHS in our work was as low as 0.03 mg L^−1^ (Table 1). Meanwhile, *Chlorella* sp., which is a food source in aquaculture, exhibited significantly resistance to PHS, DHS, and SPH. Therefore, sphingosines could serve as a new highly efficient category of species‐specific allelochemical for mitigating HABs.

**Figure 8:**
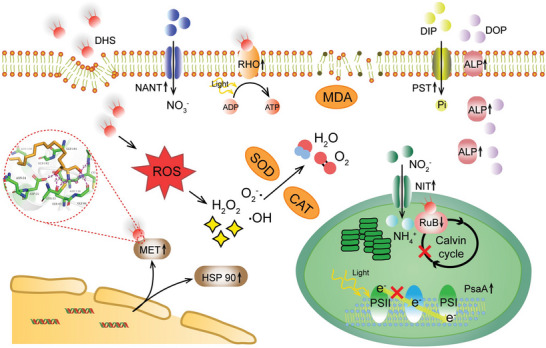
Proposed *A. carterae* inhibitory model induced by DHS. DHS inhibitory mechanism lies in the activation of autocatalytic PCD, the malfunction of the photosystem and the antioxidant system, the transportation and assimilation impediment of nutrients, transformation, and utilization disruption of light. Molecular docking simulates the potential binding model between DHS and receptors.

The investigation of the algal inhibitory mechanism revealed that DHS impeded the growth of *A. carterae* through several aspects, including the influence on photosynthesis, the interference of intracellular redox balance, the disruption of nutrients and light assimilation processes, and the induction of programmed cell death (**Figure** **8**). For instance, our results showed that activities of SOD and CAT were activated in algae cells treated with DHS, which was accompanied by the overproduction of intracellular ROS. The increase in MDA levels indicated an imbalance in the intracellular ROS dynamic equilibrium and irreversible lipid peroxidation on the cellular membrane. Phytoplankton cells have been reported to activate autocatalytic cell‐suicide under the stress of xenobiotics to promote the population stability.^[^ ^30^ ^]^ In dinoflagellate, metacaspase was suggested to play dual roles, acting as the initiator of programmed cell death and also having non‐death roles such as stress acclimation, cell division, and the aging process.^[^ ^31^ ^]^ The upregulation of metacaspase and the declined population indicated that DHS induced the programmed cell death in *A. carterae*. A similar cell death pathway mediated by DHS or PHS has also been reported in *Arabidopsis*.^[^ ^32^, ^33^ ^]^

Beyond above mechanisms observed on the target species. The unique characteristics of sphingosines lie in the fact that they are intermediates in sphingolipids biosynthesis, which are critical components of eukaryotic cell plasma membrane and endomembrane system,^[^ ^34^ ^]^ their structure shares high structural similarity with lipid bilayers. By using the lipophilicity descriptor octanol‐water partition coefficient (LogP) to represent chemicals’ cell permeability, we found that sphingosines exhibit a higher LogP than the most natural highly efficient allelochemicals (**Table** **3**). This indicates that sphingosines have high cell membrane permeability. Since sphingolipids are involved in multiple cellular and regulatory process, alterations in sphingolipids biosynthesis can be toxic.^[^ ^35^ ^]^ Therefore, the exposure to sphingosines might exert the inhibitory effects by altering normal sphingolipid metabolism, such as acting as agonist or antagonist. This potent explanation accounts for the superior antialgal property of this novel natural allelochemical. Moreover, the molecular docking results showed that abundant hydrogen bonds and hydrophobic interactions were formed between DHS and the investigated proteins (Figure 7h). These interactions might interfere with the target proteins’ acting force, resulting in changes in enzymatic conformations and deactivating these enzymes. The structural and molecular level investigation between DHS and target organs or proteins deepens our understanding on sphingosines’ transmembrane transportation and intracellular function after exposure to microalgae cells.

**Table 3:** The category, molecular weight, octanol–water partition coefficient (LogP) and effective concentration (IC_50_: mg L^‐1^) of natural highly efficient allelochemicals

Category	Allelochemical	Molecular weight	LogP	Target species	IC_50_	Reference
Polyphenolics	2‐Phenyl‐phenol	170.2	3.1	*M. aeruginosa*	0.15	[13]
Polyphenolics	5,4′‐Dihydroxyflavone	254.2	2.1	*M. aeruginosa*	0.22	[9]
Polyphenolics	Myricetin	318.2	1.2	*P. globosa*	0.31	[36]
Polyphenolics	Pyrogallic acid	126.1	0.5	*M. aeruginosa*	0.65	[37]
Alkaloids	3‐Indoleacrylic acid	187.2	2.2	*P. donghaiense*	0.14	[38]
Alkaloids	Berberine	336.4	3.6	*M. aeruginosa*	0.28	[28]
Fatty acids	Nonanoic acid	158.2	3.5	*M. aeruginosa*	0.50	[39]
Esters	Ethyl 2‐Methylacetoacetate	144.2	0.9	*M. aeruginosa*	0.49	[40]
Sphingosines	Sphinganine	301.5	5.8	*P. globosa*	0.06	This work
Sphingosines	Phytosphingosine	317.5	4.6	*P. globosa*	0.01	This work
Sphingosines	Sphingosine	299.5	5.3	*H. akashiwo*	0.12	This work
Sphingosines	*N*‐Acetylsphingosine	341.5	5.5	*H. akashiwo*	0.25	This work

### 3.2 Pronounced Antialgal Property of Agricultural Straw Manipulated by WRF

In our experiments, the straw decomposition induced by WRF improved the performance of straw extract on algal growth suppression (Figure 1a). The enhancement was attributed to the fact that the decomposition of straw leads to the release of inhibitory substances (Figure 4a), which were activated by the secondary metabolism of WRF under nutrient depletion. In agreement with the regulatory role of *T. versicolor* on canola straw, *P. chrysosporium*, *P. ostreatus*, and *T. versicolor* were also reported to enhance the impact of barley straw on algal inhibition, and the fungi‐inoculated straw reduced the inhibition lag time from 8 weeks for the fresh straw to 1 week for degraded straw.^[^ ^15^ ^]^ In fact, agricultural straws have received intensive applications in marine, brackish, and freshwater habitats,^[^ ^41^, ^42^, ^43^ ^]^ and the allelochemicals produced or released during straw decomposition were the principal substances characterizing inhibitory properties.^[^ ^44^ ^]^ Our results suggest that after the extraction of allelochemicals, the residues obtained from the fermentation of agricultural straws using WRF could be further utilized for producing feed, biofertilizers, or biofuels. This approach provides a new potential for valorizing WRF and agricultural wastes, as numerous undesirable compounds like aromatic compounds and aldehyde compounds are reduced during the extraction of allelochemicals.

To the best of our knowledge, this is the first time that DHS and PHS have been discovered as allelochemicals in agricultural straw. As intermediates of sphingolipids, both DHS and PHS were detectable in fungi and plant and are involved in a plethora of biological functions, including growth regulation, cell migration, and apoptosis.^[^ ^45^ ^]^ The de novo pathway of DHS in plants and fungi begins with the condensation of serine and palmitoyl‐CoA. The intermediate compound, 3‐ketodihydrosphingosine, is then reduced via 3‐ketodihydrosphingosine reductase to produce DHS. Finally, the introduction of a double bond can transform DHS to PHS.^[^ ^26^ ^]^ In contrast with DHS and PHS, SPH is not directly synthesized in the sphingolipid biosynthesis. Instead, it is released from the breakdown of ceramide.^[^ ^46^ ^]^ Although the sphingolipid metabolism in *T. versicolor* was not significantly activated during decomposition, the identification of both DHS and PHS in ES and the increase of DHS and PHS in ESF indicate that the elevation of these allelochemicals was derived from fungi‐mediated straw decomposition. These chemicals may be released from the decomposed straw or passively transported from fungal plasma membranes or via the active exocytosis. Given the influence of biodegradation conditions like temperature, moisture content, aeration on WRF degradation activities, optimizing cultivation may be necessary to further improve straw antialgal capability.

### 3.3 Comparative Nontarget Metabolomics Facilitates Discovering a Novel Prospective Antialgal Allelochemical

Besides the plentiful lignocellulosic materials in canola straw, hundreds of minor components were also identified in this study (Figure 3a). We used correlation analysis to identify a series of URMs responsible for the improved antialgal activity of the ESF. In the bioassay, we discovered that five of the tested chemicals exhibited antialgal allelochemical activity for the first time, except for l‐methionine. Therefore, the comparative nontarget metabolomics was effective in efficiently screening for bioactive metabolites in agricultural straw.

Plants and microbes produce very complex secondary metabolites, and bioassay‐guided isolation has been the most classical and prevalent approach for investigating potent bioactive compounds in numerous metabolites.^[^ ^47^, ^48^, ^49^ ^]^ Using solvents with different polarities, bio‐derived exudates, or extracts could be separated into various fractions. By combining this approach with other analytical techniques, such as nuclear magnetic resonance and high‐resolution mass spectrometry, numerous novel allelochemicals that mediate complex trophic interactions have been identified.^[^ ^50^, ^51^, ^52^ ^]^ In the last two decades, ≈100 plant‐derived allelochemicals have been discovered around the world.^[^ ^19^ ^]^ For instance, a novel allelochemical ethyl 2‐methylacetoacetate, was identified from the reed (*Phragmitis australis*).^[^ ^40^ ^]^ A pair of chiral flavonolignans, as anti‐cyanobacterial allelochemicals, were found in barley straw.^[^ ^14^ ^]^ However, the stepwise separation and bioactivity determination involved in traditional methods can be tedious and time consuming. In contrast, the nontarget metabolomics approach used in this study requires only a small amount of materials and can be applied to numerous unidentified metabolites observed in mass spectrometry. Furthermore, this approach enables investigation of the bioactivity of differential metabolites revealed by correlation analysis simultaneously.

The nontarget metabolomics strategy has been increasingly employed in various research areas, ranging from biotechnology to human health, and has proven to have the huge potential to generate unexpected and highly important insights.^[^ ^53^ ^]^ For instance, nontarget metabolomics was used to investigate the changes in metabolism of *Rheum tanguticum* growing under canopy and open habitats.^[^ ^54^ ^]^ Additionally, the systematic characterization of antioxidant metabolites based on nontarget metabolomics has been proposed as a new approach for the selection and characterization of desired metabolites.^[^ ^55^ ^]^ However, to the best of our knowledge, this is the first report on the identification of antialgal chemicals using nontarget metabolomics combined with a bioassay‐guided approach. In addition, nontarget metabolomics combined with data‐driven analysis allows for high‐throughput screening of voluminous natural metabolites. Moreover, the potential candidates predicted by activity correlation analysis could be validated by high‐flux screening methods like droplet‐based microfluidics^[^ ^56^ ^]^ and further investigating the bioactivity of analogs through the quantitative structure‐activity relationship analysis.^[^ ^57^ ^]^ Therefore, in addition to providing new molecular insights into canola straw extracts, our findings suggest that comparative nontarget metabolomics has the potential to be fully exploited for the efficient discovery of novel allelochemicals.

Plants‐derived allelochemicals have been suggested to be more biocompatible and biodegradable than traditional algaecides.^[^ ^8^ ^]^ Sphingosines have been reported to exhibit both growth‐inhibitory and growth‐stimulating effects,^[^ ^58^ ^]^ which largely depend on the concentration and the target species. In our study, DHS was found to selectively control the growth of the tested HABs species at the level of 0.10 mg L^−1^, significantly below its reported phytotoxic levels of 15 mg L^−1^ to duckweed (*Lemna pausicosta*).^[^ ^59^ ^]^ Quantitative structure‐activity relationships models have been widely employed in ecological risk assessment to estimate the physicochemical properties and toxicokinetic parameters associated with the absorption, distribution, metabolism, and excretion processes of chemicals.^[^ ^60^ ^]^ Using the QSAR model, the toxicity estimation results did not indicate any development toxicity or mutagenicity of the four sphingosines investigated in this work, and the toxic concentrations on nontarget organisms were much higher than their effective concentrations on target species, which indicates the prospects of developing sphingosines as a new category of algaecide.

### 3.4 Practical Prospects and Challenges in Future Product Development

Traditional algaecides such as copper‐based, hydrogen peroxide‐based and chlorine‐based agents, which can be relatively easily manufactured and have been extensively tested and applied in various aquatic systems, including canals, reservoirs and lakes.^[^ ^61^, ^62^ ^]^ However, the extrapolation of laboratory results to the field for most identified allelochemicals has been hindered, largely due to the high cost of mass production.^[^ ^19^ ^]^ As sphingolipids are substantially present in eukaryotic cell plasma membrane and tonoplast, accounting for as much as 10% of total lipids in plants.^[^ ^63^ ^]^ It is feasible to extract sphingosine from sphingolipids‐rich tissues or cells. For instance, PHS has been reported to be readily obtained in high purity from fermented yeast broth.^[^ ^64^, ^65^ ^]^ Van den Berg, et al.^[^ ^66^ ^]^ developed a commercial method for the large‐scale production of PHS with a reasonable price, as well as a simple and low‐cost synthetic approach for transferring PHS to SPH.^[^ ^67^, ^68^ ^]^ DHS has been reported to be straightforwardly synthesized from the easily prepared serine‐derived Weinreb amide^[^ ^68^ ^]^ or enantioselectively synthesized from the commercially available fatty acids.^[^ ^69^ ^]^

Although the persistence of sphingosines in natural water bodies is not well documented in the literature, it is known that various biotic and abiotic factors can affect it, such as cell density, water temperature, alkalinity, depth, stratification, and mixing.^[^ ^61^ ^]^ Direct spraying or injecting allelochemicals into waters can lead to local high concentrations and result in toxicity. Therefore, allelochemicals‐mediated sustained‐released microspheres (SRMs) have been suggested as a practical solution to enhance allelochemicals stability, equilibrize active ingredients concentration and extend the release time of products.^[^ ^70^, ^71^ ^]^ In addition, SRMs have recently been reported to reduce the ecotoxic impact of allelochemicals on submerged plants, aquatic invertebrate, and their associated microflora.^[^ ^72^, ^73^ ^]^ Although the resistance potential of target species against sphingosines remains to be investigated, the development of combinations of different active ingredients, including sphingosines, can be considered to avoid the potential adaption of target species.

To date, regulations regarding the application of aquatic algaecides, especially those involving biological control methods are quite scanty and merely focus on chemical methods. For instance, in the United States, National Pollutant Discharge Elimination System permit requirements have been in place for the application of aquatic chemical pesticide such as algaecides since 2011.^[^ ^74^ ^]^ These requirements consist of a suite of components, including problem definition, confirmation of algaecide post‐treatment concentration, assessment of algaecide performance, and response of nontarget species.^[^ ^75^ ^]^ Currently, six active ingredients, including acrolein, copper, diquat dibromide, endothall, flumioxazin, and peroxide‐based compounds, have been registered and approved by the United States Environmental Protection Agency for use as algaecides.^[^ ^76^ ^]^ All of the approved algaecides have been registered under the federal law known as the Federal Insecticide, Fungicide, and Rodenticide Act.^[^ ^77^ ^]^ Additionally, individual states may have their own regulations and permits governing the use of algaecides. In the European Union (EU), algaecides were regulated under the Biocidal Products Regulation and must undergo a thorough authorization process.^[^ ^78^ ^]^ The approved algaecide active substances in EU include hydrogen peroxide, active chlorine released from sodium hypochlorite, calcium hypochlorite, peracetic acid, etc.^[^ ^79^ ^]^ In China, the registration and evaluation of algaecides are regulated by the Ministry of Agriculture and Rural Affairs according to the measures for the Administration of Pesticide Registration.^[^ ^80^ ^]^

**Figure 9:**
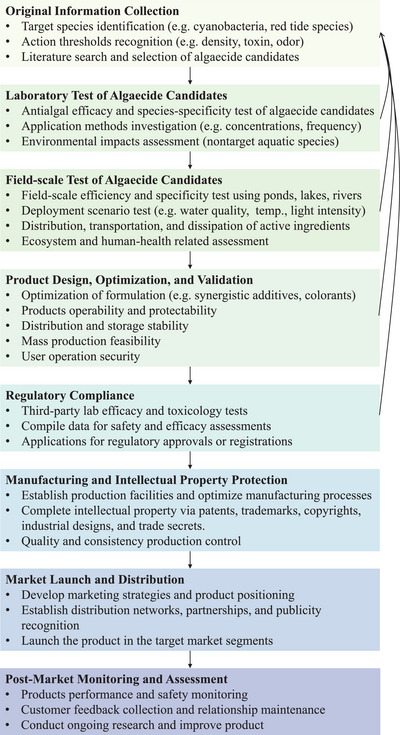
Workflow for the development of the commercialized algaecide.

The development of a marketable algaecide consists of several processes, including the original information collection; laboratory test of algaecide candidates; field‐test of algaecide candidates; product design, optimization, and validation; regulatory compliance; manufacturing and intellectual property protection; market launch and distribution; post‐market monitoring and assessment (**Figure** **9**). The development of a commercially successful algaecide poses challenges in multiple aspects. It entails significant investments of funds and time in the design and test stages to thoroughly review the products before their release, any negative findings concerning safety and environmental impacts during the research, development, and regulatory parts may necessitate the redesign and retests of the product. Additionally, a new algaecide needs to find its differentiation and competitive advantage in the market, meet customer needs, and fulfill sustainable development requirements.

Overall, the use of sphingosines for HABs mitigation should be approached with caution, and should be based on a comprehensive understanding of the site‐specific water quality conditions and characteristics. More pilot studies and field‐scale tests are needed to assess the response of target species, as well as any potential side effects on nontarget aquatic organisms and ecosystem functions, before this method can be safely applied.

## 4 Conclusions

In summary, we enhanced the antialgal property of canola straw by subjecting it to white‐rot fungi decomposition, resulting in a significant increase in its effectiveness. Transcriptomics profiles revealed that nutrient limitation at the beginning of inoculation triggered lignin decomposition by *T. versicolor*, and that the co‐expression of accessory and ligninolytic enzymes contributed to the depolymerization of lignocellulose. This, in turn, resulted in the dynamics of bioactive compounds in straw extracts and the subsequently enhancement of the antialgal property. Moreover, our comparative nontarget metabolomics analysis led us to the identification a novel category of natural infochemical, sphingosines. It exhibited superior antialgal capability with an orders of magnitude lower effective concentration than other prevalent plant‐derived algaecides on various typical blooming species including *Amphidinium carterae*, *Heterosigma akashiwo*, *Phaeocystis globosa*, *Isochrysis galbana*, *Skeletonema costatum*, and *Microcystis aeruginosa*. Algal species with commercially beneficial usage in aquaculture, namely, *Chlorella* sp. was proven to be resistant to this chemical. The growth inhibition mechanism of sphinganine against a cosmopolitan toxic bloom‐forming species (*A. carterae*) was investigated. The study indicated that inhibition lies in the activation of autocatalytic programmed cell death, the malfunction of the photosystem and the antioxidant system, as well as the disruption on CO_2_ assimilation and light absorption.

## 5 Experimental Section

### Straw, Fungi, and Algae Preparation

Fresh dried barley straw, originated from Tibet; and wheat, maize, rice, and canola straws collected from Jiangsu province, China were subsequently chopped into 3–5 cm segments, rinsed by deionized water, and dried at 40 °C for 96 h. The straw fragments were then enclosed into a 500 mL acid‐washed Erlenmeyer flask and autoclaved at 121 °C for 15 min. They were allowed to cool before inoculation or submersion.

In this study, three strains of WRF, *P. chrysosporium* BNCC 189286 and *T. versicolor* BNCC 145690, which were obtained from the Benai Chuanglian Biology Research Institute (Beijing, China), and *S. commune*, which was isolated from white‐rotted wood at Zhejiang University, China were investigated. All WRF were cultured using potato dextrose agar (PDA) and maintained at 25 °C. The constituents of PDA including 1 L potato extract, 3.0 g KH_2_PO_4_, 1.5 g MgSO_4_, 20.0 g glucose, 10 mg thiamine, and 20 g agar. All the chemicals used were of analytical grade and purchased from SINOPHARM (Shanghai, China).

*A. carterae*, a worldwide toxic “red tide” former, was provided by the Institute of Marine Biology and Pharmacology, Zhejiang University, China. The algae were sub‐cultured every week using f/2 medium^[^ ^81^ ^]^ at 25 °C with a 12: 12 h light:dark cycle (50 µmol photons m^−2^ s^−1^). The culture and maintenance of *Heterosigma akashiwo*, *Phaeocystis globosa*, *Isochrysis galbana*, and *Skeletonema costatum* were performed with the same procedures as *A. carterae*, whereas additional Na_2_SiO_3_ was added for *Skeletonema costatum. Microcystis aeruginosa* FACHB‐905 and *Chlorella* sp. were obtained from the Chinese Academy of Sciences, Freshwater Algae Culture Collection at the Institute of Hydrobiology, and BG11 medium^[^ ^82^ ^]^ was applied for the culture of these species.

### Decomposition of Canola Straw by WRF and Extraction of Decomposition Liquid

To examine the enhancement of WRF decomposition on straw extract's antialgal capability, *T. versicolor*, *P. chrysosporium*, and *S. commune* were inoculated on barley, wheat, maize, rice, and canola straws. In total, ten mycelia pellicles were inoculated with a 5 mm diameter puncher on 10 g autoclaved straw submerged in 100 mL sterile water. Then the straws were degraded under a 25 °C incubator for 90 days to obtain the extract of straw degraded by fungi (ESF). In contrast, the leachate of autoclaved straw submerged in sterile water without inoculation was defined as the extract of straw (ES).

To uncover the mechanism behind the enhanced antialgal property of straw, *T. versicolor* was selected to decompose canola straw for 18 days, using the same decomposition method as in the WRF selection part. The algal inhibition rate was documented every three days. Furthermore, the leachate of *T. versicolor* collected from fungi cultured on the PDA was defined as the extract of fungi (EF), while the extracts of ES and ESF mentioned above were obtained in the same way. Briefly, another 100 mL (200 mL for EF) of sterile water was added into ESF and ES to extract the liquid with shaking (150 rpm) for 0.5 h at 25 °C. The extracts were then filtered through a 0.22 µm filter (Millipore, MilliporeSigma, USA) and stored at −80 °C for subsequent experiments.

### Examination of ES, ESF, and EF Extracts’ Antialgal Property

To examine the ES, ESF, and EF extract's algal inhibition rate, 100 mL of *A. carterae* in exponential phase containing 10 mL straw extract were grown in 250 mL Erlenmeyer flasks, whereas 10 mL of *f*/2 medium replacing the straw extract was taken as the control group to check the inhibitory effect. After 7 days inhibition test, 2 mL of culture was transferred to a 2.5 mL tube, the algal cells were fixed with Lugol's iodine solution and checked with the microscope (BX53, Olympus, Japan) at ×200 magnification using a Neubauer chamber (NO. T729.1, Carl Roth, Germany). The algal inhibition rate was calculated using the following formula:

(1) $$\mathrm{inhibition}\mathrm{rate}\;=\;\left(N_{C}-N_{T}\right)/N_{C}\;\times 100$$

where *N*_C_ is algae density in the control group, *N*_T_ is algae density in the treated group.

### Scanning Electron Microscopy and Light Microscope Inspection

To document the impact of WRF on straw, the microstructural changes of canola straw under the decomposition of *T. versicolor* were examined by the scanning electron microscope (Sigma 500, Carl Zeiss, Germany). Pieces of 18 days’ ESF and ES were cleaned, dried, and sputter‐coated with a layer of Au before scanning. Images were captured at an acceleration voltage of 3 kV. To compare the effects of the straw extract on *A. carterae*, morphological photographs of algal cells grown in the control medium and 9 days of ESF extract were taken with a light microscope at ×200 and ×400 magnifications, respectively.

### Determination of Culture Conditions and Straw Constituents

The released reducing sugar content was assayed by the method described by Nelson with glucose as the standard.^[^ ^83^ ^]^ To determine the degree of lignocellulose degraded by WRF, the method described by Van Soest,^[^ ^84^ ^]^ measuring three macromolecules cellulose, hemicellulose, and acid‐insoluble lignin in the straw was applied. The dry weight of the degraded straw was obtained by drying the straw to a constant weight.

### RNA Extraction, cDNA Libraries Preparation, and Illumina Sequencing

To examine the transcriptome profiles of WRF during the decomposition progress, the tissues of *T. versicolor* cultured in potato dextrose agar (6 days) and canola straw (6, 9, 12, 15, and 18 days) were collected and frozen in liquid nitrogen immediately before RNA extraction. Total RNA and cDNA libraries of these materials were obtained using the Trizol Reagent (Invitrogen Life Technologies) and TruSeq RNA Sample Preparation Kit (Illumina, San Diego, CA, USA), and the cDNA libraries were further sequenced on a Hiseq X platform (Personalbio, Shanghai China). The Trinity program was used to perform *de novo* assembly of the clean reads,^[^ ^85^ ^]^ the longest transcript of every cluster was taken as the representative unigene for further analytical work.

The annotation of all unigenes was conducted with GO and KEGG databases. The fragments per kilobase of transcript per million mapped reads (FPKM) value of each unigene was estimated as the gene expression level using RNA‐seq by expectation‐maximization (RSEM) .^[^ ^86^ ^]^ DEGs were screened with a |log_2_FoldChange| > 1 and *p*‐value < 0.05 using DESeq (Bioconductor, USA), the DEGs were enriched in the Terms of Gene Ontology and KEGG pathways, and a corrected *p*‐value < 0.05 was used as the threshold to determine the GO functional terms with significant enrichment between different groups.

### UPLC‐MS/MS

To prepare the qualified sample for UPLC‐MS/MS, 100 mL ESF and ES extract were concentrated and evaporated to dryness using a rotary evaporator (IKA RV8, IKA), and 100 mg powder was re‐dissolved in methanol before analysis. The methanol extracts were analyzed with a UPLC‐MS/MS system (UPLC, Shim‐pack UFLC SHIMADZU CBM30A, https://www.shimadzu.com.cn/; MS/MS, Applied Biosystems 4500 QTRAP, https://www.appliedbiosystems.com.cn/).

The analytical conditions were as follows, UPLC column, Waters ACQUITY UPLC HSS T3 C18 (1.8 µm, 2.1 mm × 100 mm); The mobile phase consisted of solvent A, pure water with 0.04% acetic acid, and solvent B, acetonitrile with 0.04% acetic acid. Sample measurements were performed with a gradient program that employed the starting conditions of 95% A, and 5% B. Within 10 min, a linear gradient to 5% A, 95% B was programmed, and a composition of 5% A, 95% B was kept for 1 min. Subsequently, a composition of 95% A, and 5.0% B was adjusted within 0.10 min and kept for 2.9 min. The column oven was set to 40 °C; The injection volume was 4 µL. The effluent was alternatively connected to an ESI‐triple quadrupole‐linear ion trap (Q TRAP)‐MS.

Linear ion trap (LIT) and triple quadrupole (QQQ) scans were acquired on a Q TRAP, API 4500 Q TRAP UPLC/MS/MS System, equipped with an ESI Turbo Ion‐Spray interface, operating in positive and negative ion mode and controlled by Analyst 1.6.3 (AB Sciex, USA). The ESI source operation parameters were as follows: an ion source, turbo spray; source temperature 550 °C; ion spray voltage 5500 V (positive ion mode)/−4500 V (negative ion mode); ion source gas I, gas II, curtain gas was set at 50, 60, and 30.0 psi, respectively; the collision gas was high. Instrument tuning and mass calibration were performed with 10 and 100 µmol/L polypropylene glycol solutions in QQQ and LIT modes, respectively. QQQ scans were acquired as multiple reaction monitoring (MRM) experiments with collision gas (nitrogen) set to 5 psi. Declustering potential (DP) and collision energy (CE) for individual MRM transitions were done with further DP and CE optimization.^[^ ^87^ ^]^ A specific set of MRM transitions were monitored for each period according to the metabolites eluted within this period.

### Metabolites Identification and Differentially Regulated Metabolites Enrichment

The qualitative and quantitative analyses of the metabolites were conducted in a self‐constructed database (MWDB) and public metabolites database using primary and secondary mass spectrometry. The metabolism molecular network was performed with structure similarity and a threshold value of Tanimoto index at 0.75 was applied to create the network,^[^ ^24^ ^]^ the molecular network data were visualized using the layout algorithm Fruchterman‐Reingold with Gephi 0.9.2.^[^ ^88^ ^]^

Differentially regulated metabolites between groups were determined by variable importance project (VIP) ≥ 1 and absolute fold change ≥ 1 or fold change ≤ 0.5. VIP values were extracted from the orthogonal partial least squares discrimination analysis (OPLS‐DA) result, which also contains score plots and permutation plots, these plots were generated using the R package MetaboAnalystR. The data were log_2_ transformed and mean centering before OPLS‐DA. To avoid overfitting, a permutation test (200 permutations) was performed. KEGG pathway database enrichment analyses of differentially regulated metabolites were further implemented to analyze the significantly enriched pathways with a hypergeometric test, and a threshold of *p* < 0.05.^[^ ^89^ ^]^

### Comparative Nontarget Metabolomics Analysis

To conduct a comparative nontarget metabolomics analysis, the identified metabolites were first pairwise compared between fungi‐decomposed straw extracts (ESF on the 6th, 12th, 15th and 18th day) and submerged straw extract (ES on the 18th day) for URMs. A metabolites assemblage consists of 62 shared URMs was then acquired. Afterward, the relative abundance of shared URMs and the algal inhibition rate over the whole decomposition were correlated using Pearson correlation coefficients. Based on the correlation coefficient matrix, the metabolites that might dominate the inhibitory effects of decomposed straw extracts were selected with the threshold value of *r* >0.75, *p* <0.05. Eventually, 39 URMs significantly correlated with the algal inhibition rate were selected.

### Co‐occurrence between URMs and DEGs

The correlation and visualization between shared URMs and DEGs involved in the carbohydrate and lignin metabolism were conducted using R v.4.1.0^[^ ^90^ ^]^ and Cytoscape v.3.9.1,^[^ ^91^ ^]^ Pearson correlation coefficients were employed for the correlation analysis with a threshold of *p* <0.05.

### Inhibitory Effects Confirmation of URMs and Sphingosines on Test Species, and the Toxicity Estimation on Nontarget Species

To confirm the antialgal ability of URMs and sphingosines, l‐methionine, choline alfoscerate, 3‐hydroxyanthranilic acid, and PHS were purchased from Aladdin (Shanghai, China), and DL‐alanyl‐DL‐phenylalanine, DHS, SPH, and APH purchased from J&K Chemicals (Beijing, China). Choline alfoscerate, DHS, PHS, SPH, APH, and 3‐hydroxyanthranilic acid were dissolved in DMSO (Aladdin, China) to obtain a stock concentration of 20 g L^−1^. l‐methionine and DL‐alanyl‐DL‐phenylalanine were dissolved into sterile water to prepare the stock solution. The stock solutions were then diluted to a concentration of working solution using the corresponding solvent before application. The working solution ratio used was 1‰ (v/v) of the culture system, which had proved that DMSO will have no effects on the growth of algae, and the microalgae inoculated in the f/2 medium was defined as the control. The initial cell density was selected based on the cell densities of algae blooms that occurred in natural aquatic environment, and the density of *A. carterae*, *H. akashiwo* and *S. costatum* were 5.0 × 10^4^ cells mL^−1^, *P. globosa*, *I. galbana*, and *Chlorella. sp*. was 5.0 × 10^5^ cells mL^−1^, *M. aeruginosa* was 1.0 × 10^6^ cells mL^−1^. The inhibition rate was calculated as mentioned above, and the antialgal property of commercial standards was converted to 72h‐IC_50_ using a probit regression method.^[^ ^92^ ^]^

The toxicity estimation, including the assessment of development toxicity, mutagenicity, as well as the determination of 50% lethal (LC_50_) and growth inhibition (IGC_50_) concentration of sphingosines, was conducted using the Toxicity Estimation Software Tool 5.1 software released by the U.S. Environmental Protection Agency.^[^ ^93^ ^]^

### Physiological Parameters Analysis of *A. Carterae*

DHS was found to have an outstanding inhibitory effect on the growth of *A. carterae* described above, algal cells treated under the 120h‐IC_50_ (0.13 mg L^−1^, 100 mL) were performed to analyze the impairment of DHS on the photosynthetic system, and antioxidant system. To determine the effects of DHS on photosynthetic efficiency, the maximum quantum yield of photosystem II (Fv/Fm) and the slope of the rapid light curve (*α*) were measured by a fluorescence monitor (MINI‐PAM‐ II/R, Walz, Germany) in vivo.

The intracellular ROS level was measured by the fluorescent probe, 2’,7’‐ dichlorodihydrofluorescein diacetate (DCFH‐DA) according to the manufacturer's instruction (Beyotime Biotechnology, Shanghai, China). To measure the activities of antioxidative enzymes (SOD, CAT) and the peroxidation products of lipids (MDA), algal cells were collected and centrifuged (3000 × *g*, 5 min, 4 °C) using a refrigerated centrifuge (Allegra X‐30R, Beckman Coulter), then the precipitate was resuspended in 2 mL of phosphate‐buffered saline (50 mm, pH 7.8). The cells were next disrupted using an ultrasonic crusher (JY92‐IIN, Scientz, China), followed by centrifugation (3000 × *g*, 10 min, 4 °C), the supernatant was then stored at −80 °C before determination. The measurement of SOD, CAT, and MDA was applied using the assay kit (Nanjing Jiancheng Bioengineering Institute, China) following the manufacturer's operation manuals.

### RT‐qPCR Analysis of *A. Carterae*

For the RT‐qPCR analysis of *A. carterae*, genes related to photosynthesis, environmental stress, ATP generation, and nutrients assimilation were chosen to check their expression level under the treatment of DHS. Algal cells were harvested through centrifugation (3000 × *g*, 5 min, 4 °C), the total RNA was extracted with Trizol Reagent (Invitrogen Life Technologies, USA), and the cDNA was reverse transcript by the digested RNA using the Reverse Transcription kit (Qiagen, Germany) strictly following the manufacturer's instructions. The reaction was performed on a real‐time fluorescence quantification system (Light Cycler 480 II, Roche, Switzerland), and the thermal cycle conditions were: one preheating stage at 95 °C for 5 min, followed by 40 cycles of 95 °C for 15 s and 60 °C for 30 s. 18S rRNA was taken as the housekeeping gene as its high stability in *A. carterae* under different environmental conditions, the relative expression of target genes was normalized with 18S rRNA using 2^−△△Ct^, see a method in Effiong et al.^[^ ^38^ ^]^ The primers for all genes were listed in Table S5, Supporting Information.

### Molecular Docking

The homology structures of all target proteins were assembled using homology modeling software EasyModeller 4.0 based on the protein sequences downloaded from National Center for Biotechnology Information.^[^ ^94^ ^]^ Molecular software LeDock was performed to process these proteins automatically to produce docking input files. To obtain an appropriate binding site, POCASA 1.1 was applied to predict possible binding pockets of all target proteins. The maximum number of binding poses was 20 and the root‐mean‐squared deviation (RMSD) was set as 1.0 Å amid the docking process. The lowest binding energy and conformation of each structural complex were used for further analysis, and the visualization of every optimal protein‐ligand structure was performed by PyMOL 2.5.0 software.^[^ ^95^ ^]^

### Statistical Analysis

Data are presented as means ± standard error of independent experiments in this work. Levene and Shapiro–Wilk tests were performed to check the homogeneity and normality of the algal inhibition rate, and the two‐tailed unpaired Student's t‐tests were used to check the variance between pairwise groups (*p < 0.05, **p < 0.01, ***p < 0.001). All variance tests were conducted with SPSS 22.0 (IBM Corp., NY, USA) and results were analyzed using Origin 2023 (OriginLab Corp., MA, USA). The gene expression level and metabolite abundance were normalized before the construction of the molecular network and the heatmaps. Pearson correlation analysis was also performed in SPSS 22.0 (IBM Corp., NY, USA), and differences with *p* < 0.05 were considered significant (*p < 0.05, **p < 0.01, ***p < 0.001).

## Conflict of Interest

The authors declare no conflict of interest.

## Author Contributions

X.X., J.H., and E.K. designed the experiments; J.H., E.K., conducted the experiments; T.T., S.H., M.L., and Y.H. assisted the analysis of metabolites; S.Y. and Y.P. assisted the analysis of transcriptome; C.X., J.Z. confirmed the inhibitory effects of DHS; X.X. and H.J. analyzed and interpreted the results; J.H. and E.K. wrote the article; and X.X, M.H. revised the article.

## Supporting information

Supporting Information

## Data Availability

The data that support the findings of this study are available from the corresponding author upon reasonable request.

## References

[1] C. B. Field , M. J. Behrenfeld , J. T. Randerson , P. Falkowski , Science 1998, 281, 237. 9657713 10.1126/science.281.5374.237

[2] J. C. Ho , A. M. Michalak , N. Pahlevan , Nature 2019, 574, 667. 31610543 10.1038/s41586-019-1648-7

[3] C. J. Gobler , O. M. Doherty , T. K. Hattenrath‐Lehmann , A. W. Griffith , Y. Kang , R. W. Litaker , Proc. Natl. Acad. Sci. U. S. A. 2017, 114, 4975. 28439007 10.1073/pnas.1619575114 PMC5441705

[4] C. J. Gobler , Harmful Algae 2020, 91, 101731. 32057341 10.1016/j.hal.2019.101731

[5] M. Liu , J. He , Y. Huang , T. Tang , J. Hu , X. Xiao , Water Res. 2022, 219, 118591. 35598469 10.1016/j.watres.2022.118591

[6] M. Liu , J. Hu , Y. Huang , J. He , K. Effiong , T. Tang , S. Huang , Y. D. Perianen , F. Wang , M. Li , X. Xiao , Environ. Res. Lett. 2023, 18, 014034.

[7] J. Huisman , G. A. Codd , H. W. Paerl , B. W. Ibelings , J. M. H. Verspagen , P. M. Visser , Nat. Rev. Microbiol. 2018, 16, 471. 29946124 10.1038/s41579-018-0040-1

[8] B. Li , Y. Yin , L. Kang , L. Feng , Y. Liu , Z. Du , Y. Tian , L. Zhang , Chemosphere 2021, 267, 128869. 33218724 10.1016/j.chemosphere.2020.128869

[9] C. Xu , S. Yu , J. Hu , K. Effiong , Z. Ge , T. Tang , X. Xiao , Sci. Total Environ. 2022, 838, 156055. 35598674 10.1016/j.scitotenv.2022.156055

[10] K. Effiong , J. Hu , C. Xu , T. Tang , H. Huang , J. Zeng , X. Xiao , J. Renewable Mater. 2020, 8, 461.

[11] I. M. Welch , P. R. F. Barrett , M. T. Gibson , I. Ridge , J. Appl. Phycol. 1990, 2, 231.

[12] N. C. Everall , D. R. Lees , Water Res. 1997, 31, 614.

[13] D. Murray , B. Jefferson , P. Jarvis , S. A. Parsons , Environ. Technol. 2010, 31, 455. 20450120 10.1080/09593331003663294

[14] X. Xiao , H. Huang , Z. Ge , T. B. Rounge , J. Shi , X. Xu , R. Li , Y. Chen , Environ. Microbiol. 2014, 16, 1238. 24034604 10.1111/1462-2920.12226

[15] D. Murray , S. A. Parsons , P. Jarvis , B. Jefferson , Water Res. 2010, 44, 1373. 20042214 10.1016/j.watres.2009.11.014

[16] J. R. Newman , P. R. F. Barrett , J. Aquat. Plant Manage. 1993, 31, 203.

[17] R. S. Iredale , A. T. McDonald , D. G. Adams , Water Res. 2012, 46, 6095. 22989994 10.1016/j.watres.2012.08.040

[18] C. S. Evans , M. V. Dutton , F. Guillén , R. G. Veness , FEMS Microbiol. Rev. 1994, 13, 235.

[19] X. Zhu , G. Dao , Y. Tao , X. Zhan , H. Hu , J. Hazard. Mater. 2021, 401, 123403. 32659587 10.1016/j.jhazmat.2020.123403

[20] J. Watrous , P. Roach , T. Alexandrov , B. S. Heath , J. Y. Yang , R. D. Kersten , M. van der Voort , K. Pogliano , H. Gross , J. M. Raaijmakers , B. S. Moore , J. Laskin , N. Bandeira , P. C. Dorrestein , Proc. Natl. Acad. Sci. U. S. A. 2012, 109, E1743. 22586093 10.1073/pnas.1203689109 PMC3387089

[21] D. D. Nguyen , C.‐H. Wu , W. J. Moree , A. Lamsa , M. H. Medema , X. Zhao , R. G. Gavilan , M. Aparicio , L. Atencio , C. Jackson , J. Ballesteros , J. Sanchez , J. D. Watrous , V. V. Phelan , C. van de Wiel , R. D. Kersten , S. Mehnaz , R. De Mot , E. A. Shank , P. Charusanti , H. Nagarajan , B. M. Duggan , B. S. Moore , N. Bandeira , B. Ø. Palsson , K. Pogliano , M. Gutiérrez , P. C. Dorrestein , Proc. Natl. Acad. Sci. U. S. A. 2013, 110, E2611. 23798442 10.1073/pnas.1303471110 PMC3710860

[22] K. Kleigrewe , J. Almaliti , I. Y. Tian , R. B. Kinnel , A. Korobeynikov , E. A. Monroe , B. M. Duggan , V. Di Marzo , D. H. Sherman , P. C. Dorrestein , L. Gerwick , W. H. Gerwick , J. Nat. Prod. 2015, 78, 1671. 26149623 10.1021/acs.jnatprod.5b00301 PMC4681511

[23] M. Wang , J. J. Carver , V. V. Phelan , L. M. Sanchez , N. Garg , Y. Peng , D. D. Nguyen , J. Watrous , C. A. Kapono , T. Luzzatto‐Knaan , C. Porto , A. Bouslimani , A. V. Melnik , M. J. Meehan , W.‐T. Liu , M. Crüsemann , P. D. Boudreau , E. Esquenazi , M. Sandoval‐Calderón , R. D. Kersten , L. A. Pace , R. A. Quinn , K. R. Duncan , C.‐C. Hsu , D. J. Floros , R. G. Gavilan , K. Kleigrewe , T. Northen , R. J. Dutton , D. Parrot , et al., Nat. Biotechnol. 2016, 34, 828. 27504778 10.1038/nbt.3597 PMC5321674

[24] F. Fontaine , E. Bolton , Y. Borodina , S. H. Bryant , Chem. Cent. J. 2007, 1, 12. 17880744 10.1186/1752-153X-1-12 PMC1994057

[25] C.‐C. Chen , L. Dai , L. Ma , R.‐T. Guo , Nat. Rev. Chem. 2020, 4, 114. 37128024 10.1038/s41570-020-0163-6

[26] R. Mashima , T. Okuyama , M. Ohira , Future Sci. OA 2020, 6, FSO434. 10.2144/fsoa-2019-0094 PMC6920741 31915535

[27] Y. A. Hannun , L. M. Obeid , Nat. Rev. Mol. Cell Biol. 2018, 19, 175. 29165427 10.1038/nrm.2017.107 PMC5902181

[28] D. Jančula , J. Gregorová , B. Maršálek , Aquac. Res. 2010, 41, 598.

[29] Q. Zhu , B. Wu , L. Zhao , Ecotox. Environ. Safe. 2021, 208, 111423. 10.1016/j.ecoenv.2020.111423 33075586

[30] C. Gallo , G. d'Ippolito , G. Nuzzo , A. Sardo , A. Fontana , Nat. Commun. 2017, 8, 1292. 29101388 10.1038/s41467-017-01300-1 PMC5670183

[31] X. Huang , B. Huang , J. Chen , X. Liu , J. Plankton Res. 2016, 38, 83.

[32] M. Saucedo‐García , A. Guevara‐García , A. González‐Solís , F. Cruz‐García , S. Vázquez‐Santana , J. E. Markham , M. G. Lozano‐Rosas , C. R. Dietrich , M. Ramos‐Vega , E. B. Cahoon , M. Gavilanes‐Ruíz , New Phytol. 2011, 191, 943. 21534970 10.1111/j.1469-8137.2011.03727.x

[33] C. Lachaud , E. Prigent , P. Thuleau , S. Grat , D. Da Silva , C. Brière , C. Mazars , V. Cotelle , Cell Death Differ. 2013, 20, 209. 22935611 10.1038/cdd.2012.114 PMC3554326

[34] E. Huby , J. A. Napier , F. Baillieul , L. V. Michaelson , S. Dhondt‐Cordelier , New Phytol. 2020, 225, 659. 31211869 10.1111/nph.15997 PMC6973233

[35] A. H. Merrill Jr. , J. Biol. Chem. 2002, 277, 25843. 12011104 10.1074/jbc.R200009200

[36] X. Xiao , C. Li , H. Huang , Y. P. Lee , Environ. Sci. Pollut. Res. 2019, 26, 23763. 10.1007/s11356-019-05482-7 31209750

[37] S. Nakai , Y. Inoue , M. Hosomi , A. Murakami , Water Res. 2000, 34, 3026.

[38] K. Effiong , J. Hu , C. Xu , Y. Zhang , S. Yu , T. Tang , Y. Huang , Y. Lu , W. Li , J. Zeng , X. Xiao , Mar. Pollut. Bull. 2022, 178, 113657. 35452911 10.1016/j.marpolbul.2022.113657

[39] S. Nakai , S. Yamada , M. Hosomi , Hydrobiologia 2005, 543, 71.

[40] Y.‐J. Men , H.‐Y. Hu , F.‐M. Li , J. Appl. Phycol. 2007, 19, 521.

[41] D. E. Terlizzi , M. D. Ferrier , E. A. Armbrester , K. A. Anlauf , J. Appl. Phycol. 2002, 14, 275.

[42] E. F. Brownlee , S. G. Sellner , K. G. Sellner , J. Appl. Phycol. 2003, 15, 525.

[43] B. J. Lim , J. H. Park , J. W. Jung , K. S. Hwang , M. S. Son , C. H. Lim , J. E. Na , S. G. Kim , H. M. Chai , K. A. Seo , J. H. Han , S. S. Park , J. K. Park , Desalin. Water Treat. 2015, 54, 3728.

[44] D. Jancula , B. Marsalek , Chemosphere 2011, 85, 1415. 21925702 10.1016/j.chemosphere.2011.08.036

[45] T. Harayama , H. Riezman , Nat. Rev. Mol. Cell Biol. 2018, 19, 281. 29410529 10.1038/nrm.2017.138

[46] A. H. Merrill , E. M. Schmelz , D. L. Dillehay , S. Spiegel , J. A. Shayman , J. J. Schroeder , R. T. Riley , K. A. Voss , E. Wang , Toxicol. Appl. Pharmacol. 1997, 142, 208. 9007051 10.1006/taap.1996.8029

[47] G. Pishchany , E. Mevers , S. Ndousse‐Fetter , D. J. Horvath , C. R. Paludo , E. A. Silva‐Junior , S. Koren , E. P. Skaar , J. Clardy , R. Kolter , Proc. Natl. Acad. Sci. U. S. A. 2018, 115, 10124. 30228116 10.1073/pnas.1807613115 PMC6176635

[48] S. B. Ng , Y. Kanagasundaram , H. Fan , P. Arumugam , B. Eisenhaber , F. Eisenhaber , Nat. Biotechnol. 2018, 36, 570. 29979661 10.1038/nbt.4187

[49] J. MacAlpine , M. Daniel‐Ivad , Z. Liu , J. Yano , N. M. Revie , R. T. Todd , P. J. Stogios , H. Sanchez , T. R. O'Meara , T. A. Tompkins , A. Savchenko , A. Selmecki , A. O. Veri , D. R. Andes , P. L. Fidel , N. Robbins , J. Nodwell , L. Whitesell , L. E. Cowen , Nat. Commun. 2021, 12, 6151. 34686660 10.1038/s41467-021-26390-w PMC8536679

[50] G. Aliotta , A. Molinaro , P. Monaco , G. Pinto , L. Previtera , Phytochemistry 1992, 31, 109.

[51] S. Chen , T. Zheng , C. Ye , W. Huannixi , Z. Yakefu , Y. Meng , X. Peng , Z. Tian , J. Wang , Y. Ma , Y. Yang , Z. Ma , Z. Zuo , Ecotox. Environ. Safe. 2018, 163, 594. 10.1016/j.ecoenv.2018.07.115 30077157

[52] M. DellaGreca , A. Fiorentino , M. Isidori , P. Monaco , F. Temussi , A. Zarrelli , Phytochemistry 2001, 58, 299. 11551554 10.1016/s0031-9422(01)00203-5

[53] D. C. Sévin , A. Kuehne , N. Zamboni , U. Sauer , Curr. Opin. Biotechnol. 2015, 34, 1. 25461505 10.1016/j.copbio.2014.10.001

[54] F. Xiong , X. Nie , L. Yang , L. Wang , J. Li , G. Zhou , BMC Plant Biol. 2021, 21, 119. 33639841 10.1186/s12870-021-02897-8 PMC7913229

[55] S. Lee , D.‐G. Oh , D. Singh , H. J. Lee , G. R. Kim , S. Lee , J. S. Lee , C. H. Lee , Metabolites 2019, 9, 186.31527409

[56] Y. Wang , R. Jin , B. Shen , N. Li , H. Zhou , W. Wang , Y. Zhao , M. Huang , P. Fang , S. Wang , P. Mary , R. Wang , P. Ma , R. Li , Y. Tian , Y. Cao , F. Li , L. Schweizer , H. Zhang , Sci. Adv. 2021, 7, eabe3839. 34117053 10.1126/sciadv.abe3839 PMC8195480

[57] T. Tang , H. Huang , J. Hu , S. Huang , M. Liu , S. Yu , X. Xiao , Aquat. Toxicol. 2023, 256, 106420. 36774780 10.1016/j.aquatox.2023.106420

[58] R. T. Riley , E. Wang , J. J. Schroeder , E. R. Smith , R. D. Plattner , H. Abbas , H. S. Yoo , A. H. Merrill Jr. , Nat. Toxins 1996, 4, 3. 8680751 10.1002/19960401nt2

[59] T. Tanaka , H. K. Abbas , S. O. Duke , Phytochemistry 1993, 33, 779.

[60] A. Grech , C. Brochot , J.‐L. Dorne , N. Quignot , F. Y. Bois , R. Beaudouin , Sci. Total Environ. 2017, 578, 1. 27842969 10.1016/j.scitotenv.2016.10.146

[61] J. Hu , D. E. Berthold , Y. Wang , X. Xiao , H. D. Laughinghouse , Harmful Algae 2022, 120, 102347. 36470610 10.1016/j.hal.2022.102347

[62] T. Tang , E. Kokoette , J. Hu , C. Li , X. Xiao , Front. Mar. Sci. 2021, 8, 618950.

[63] L. Dunn , New Phytol 2004, 161, 677. 33873728 10.1111/j.1469-8137.2004.00992.x

[64] R. C. Dickson , R. L. Lester , Biochim. Biophys. Acta Gen. Subj. 1999, 1426, 347. 10.1016/s0304-4165(98)00135-4 9878820

[65] R. C. Dickson , R. L. Lester , Biochim. Biophys. Acta, Mol. Cell Biol. Lipids 1999, 1438, 305.

[66] R. J. B. H. N. Van den Berg , T. J. Boltje , C. P. Verhagen , R. E. J. N. Litjens , G. A. van der Marel , H. S. Overkleeft , J. Org. Chem. 2006, 71, 836. 16409007 10.1021/jo0520240

[67] R. J. B. H. N. van den Berg , C. G. N. Korevaar , G. A. van der Marel , H. S. Overkleeft , J. H. van Boom , Tetrahedron Lett. 2002, 43, 8409.

[68] R. C. So , R. Ndonye , D. P. Izmirian , S. K. Richardson , R. L. Guerrera , A. R. Howell , J. Org. Chem. 2004, 69, 3233. 15104474 10.1021/jo030355b

[69] R. Ait‐Youcef , X. Moreau , C. Greck , J. Org. Chem. 2010, 75, 5312. 20578745 10.1021/jo1003899

[70] H. Huang , X. Xiao , F. Lin , H. P. Grossart , Z. Nie , L. Sun , C. Xu , J. Shi , Water Res. 2016, 95, 113. 26986500 10.1016/j.watres.2016.02.058

[71] L. Ni , S. Rong , G. Gu , L. Hu , P. Wang , D. Li , F. Yue , N. Wang , H. Wu , S. Li , Chemosphere 2018, 212, 654. 30173112 10.1016/j.chemosphere.2018.08.045

[72] B. Li , D. Xu , L. Feng , Y. Liu , L. Zhang , Sci. Total Environ. 2023, 871, 161888. 36731566 10.1016/j.scitotenv.2023.161888

[73] B. Li , Y. Yin , X. Zhou , L. Feng , Y. Liu , Z. Du , Y. Tian , L. Zhang , J. Environ. Sci. 2023, 124, 205. 10.1016/j.jes.2021.10.020 36182132

[74] U. S. E. P. Agency , National Pollutant Discharge Elimination System (NPDES)‐Pesticide Permitting, https://www.epa.gov/npdes/pesticide‐permitting (accessed: May 2023).

[75] F. A. Kibuye , A. Zamyadi , E. C. Wert , Harmful Algae 2021, 109, 102099. 34815017 10.1016/j.hal.2021.102099

[76] A. J. Calomeni , T. D. Geer , K. J. Iwinksi , J. H. Rodgers, Jr. , J. D. Madsen , R. M. Wersal , J. Integr. Pest Manage. 2017, 8, 27.

[77] U. S. E. P. Agency , Federal Insecticide, Fungicide, and Rodenticide Act (FIFRA) and Federal Facilities, https://www.epa.gov/enforcement/federal‐insecticide‐fungicide‐and‐rodenticide‐act‐fifra‐and‐federal‐facilities (accessed: May 2023).

[78] E. C. Agency , Understanding Biocidal Products Regulation (BPR), https://echa.europa.eu/regulations/biocidal‐products‐regulation/understanding‐bpr (accessed: May, 2023).

[79] Information on biocides, https://echa.europa.eu/information‐on‐chemicals/biocidal‐products (accessed: May 2023).

[80] P.R.C. Ministry of Agriculture, Measures for the Administration of Pesticide Registration, http://www.moa.gov.cn/govpublic/CYZCFGS/202201/t20220127_6387814.htm (accessed: May 2023).

[81] R. R. L. Guillard , Culture of Phytoplankton for Feeding Marine Invertebrates. Culture of Marine Invertebrate Animals, Springer, Berlin 1975.

[82] L. Wang , H. Wang , X. Chen , Y. Xu , T. Zhou , X. Wang , Q. Lu , R. Ruan , Bioresour. Technol. 2018, 253, 188. 29353749 10.1016/j.biortech.2018.01.039

[83] N. A. Nelson , J. Biol. Chem. 1944, 153, 375.

[84] P. J. Van Soest , J. B. Robertson , B. A. Lewis , J. Dairy Sci. 1991, 74, 3583. 1660498 10.3168/jds.S0022-0302(91)78551-2

[85] X. Li , L. Yan , Q. Li , H. Tan , J. Zhou , R. Miao , L. Ye , W. Peng , X. Zhang , W. Tan , B. Zhang , Sci. Rep. 2019, 9, 5641. 30948778 10.1038/s41598-019-42157-2 PMC6449350

[86] M. Yang , L. Lu , S. Li , J. Zhang , Z. Li , S. Wu , Q. Guo , H. Liu , C. Wang , Toxins 2019, 11, 70. 30691218 10.3390/toxins11020070 PMC6410012

[87] W. Chen , L. Gong , Z. Guo , W. Wang , H. Zhang , X. Liu , S. Yu , L. Xiong , J. Luo , Mol. Plant 2013, 6, 1769. 23702596 10.1093/mp/sst080

[88] M. Bastian , S. Heymann , M. Jacomy , Proc. Int. AAAI Conf. Web Soc. Media 2009, 3, 361.

[89] M. Yang , J. Yang , L. Su , K. Sun , D. Li , Y. Liu , H. Wang , Z. Chen , T. Guo , Plant Sci. 2019, 289, 110282. 31623771 10.1016/j.plantsci.2019.110282

[90] R. Core Team , R: A Language and Environment for Statistical Computing; R Foundation for Statistical Computing, http://www.r‐project.org/index.html (accessed: May, 2022).

[91] P. Shannon , A. Markiel , O. Ozier , N. S. Baliga , J. T. Wang , D. Ramage , N. Amin , B. Schwikowski , T. Ideker , Genome Res. 2003, 13, 2498. 14597658 10.1101/gr.1239303 PMC403769

[92] C. Xu , S. Huang , Y. Huang , K. Effiong , S. Yu , J. Hu , X. Xiao , Sci. Total Environ. 2020, 714, 136737. 31982752 10.1016/j.scitotenv.2020.136737

[93] U. S. E. P. Agency , Toxicity Estimation Software Tool (TEST), https://www.epa.gov/chemical‐research/toxicity‐estimation‐software‐tool‐test#pubs (accessed: May 2023).

[94] B. K. Kuntal , P. Aparoy , P. Reddanna , BMC Res. Notes 2010, 3, 226. 20712861 10.1186/1756-0500-3-226 PMC2936912

[95] J. Yu , X. Li , H. Liu , Y. Peng , X. Wang , Y. Xu , J. Mol. Struct. 2021, 1223, 128978.

