# Supplementary material for: Natural Algaecide Sphingosines Identified in Hybrid Straw Decomposition Driven by White‐Rot Fungi
Source: Adv Sci (Weinh). 2023 Jul 3;10(25):2300569. doi: 10.1002/advs.202300569 (PMC10477863; doi:10.1002/advs.202300569)
Supplement: Supplementary file 1 — Supporting Information [file ADVS-10-2300569-s001.pdf]

ADVANCED SCIENCE
Open Access

Supporting Information

for *Adv. Sci.*, DOI 10.1002/advs.202300569

Natural Algaecide Sphingosines Identified in Hybrid Straw Decomposition Driven by White-Rot Fungi

*Jing Hu, Effiong Kokoette, Caicai Xu, Shitao Huang, Tao Tang, Yiyi Zhang, Muyuan Liu, Yuzhou Huang, Shumiao Yu, Jie Zhu, Marianne Holmer and Xi Xiao**

Supplementary Materials

Natural algaecide sphingosines identified in hybrid straw decomposition driven by white-rot fungi

Jing Hu [1,2], Effiong Kokoette [1,2], Caicai Xu [1], Shitao Huang [1], Tao Tang [1], Yiyi Zhang [1], Muyuan Liu [1,4], Yuzhou Huang [1], Shumiao Yu [1], Jie Zhu [1], Marianne Holmer [3], Xi Xiao* [1,2,4]

[1] Ocean College, Zhejiang University, #1 Zheda Road, Zhoushan, Zhejiang 316021, China
[2] Key Laboratory of Marine Ecological Monitoring and Restoration Technologies of Ministry of Natural Resources, Shanghai, 201206, China
[3] Department of Biology, University of Southern Denmark, Odense, 5230, Denmark
[4] Key Laboratory of Watershed Non-point Source Pollution Control and Water Eco-security of Ministry of Water Resources, College of Environmental and Resources Sciences, Zhejiang University, Hangzhou, Zhejiang 310058, China

*Corresponding author:
Dr. Xi Xiao
Prof., Ocean College, Zhejiang University,
Email: xi@zju.edu.cn,
Mobile: +86-15088785518
Fax: +86-0580 2092891

Supplementary Figures

Supplementary Figure S1 Enriched GO terms in respect of biological process, cellular component, molecular function, and top 20 enriched KEGG pathways in annotation result.

Supplementary Figure S2 Principal component analysis (PCA) of straw extract metabolites over different decomposition conditions.

Supplementary Tables

Supplementary Table S1 Decomposition capacity of Trametes versicolor on canola straw and reducing sugar in fungi rotted straw extract.

Supplementary Table S2 Ion abundance of metabolites among different straw extracts.

Supplementary Table S3 Variable importance in projection and fold change of differentially regulated metabolites between the pairwise comparison of 18 day's straw extracts.

Supplementary Table S4 Development toxicity, mutagenicity, 50% lethal (LC_{50}) and growth inhibition (IGC_{50}) concentration of sphingosines on nontarget organisms.

Supplementary Table S5 Information of primers used in RT-qPCR for *A. carterae*.

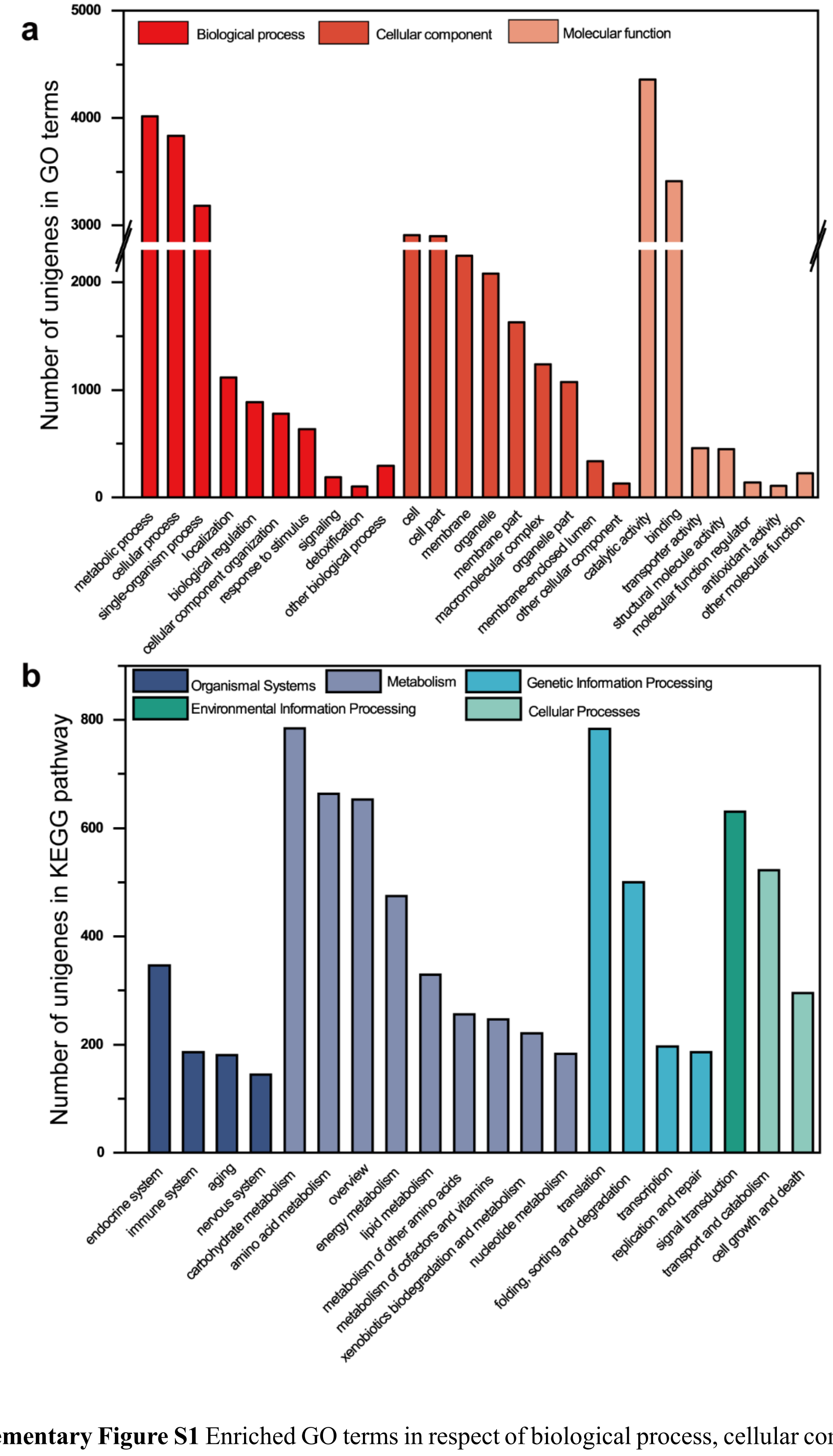

Supplementary Figure S1 Enriched GO terms in respect of biological process, cellular component, molecular function (a), and top 20 enriched KEGG pathways (b) in annotation result.

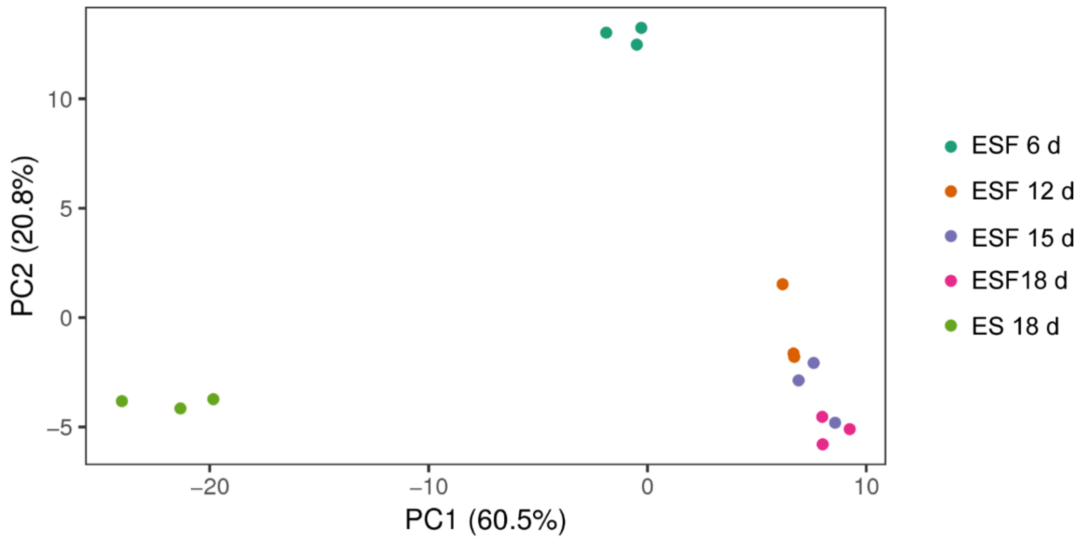

Supplementary Figure S2 Principal component analysis (PCA) of straw extract metabolites over different decomposition conditions. The PCA was analyzed and presented by R (www.r-project.org/). ESF extract of white-rot fungi decomposed straw, ES extract of sterile water submerged straw.

Supplementary Table S1

Decomposition capacity of Trametes versicolor on canola straw and reducing sugar in fungi rotted straw extract(mean ± standard error, n=3).

	Dry weight		Cellulose		Hemicellulose		Lignin		Reducing Sugar	
Days	Mean	SE	Mean	SE	Mean	SE	Mean	SE	Mean	SE
0	10.00	0.00	5.21	0.17	1.50	0.09	1.62	0.08	-	-
3	9.18	0.01	5.18	0.06	1.49	0.06	1.62	0.04	82.59	2.25
6	8.80	0.13	4.43	0.15	1.40	0.10	1.60	0.06	747.92	62.92
9	8.37	0.10	4.31	0.11	1.36	0.03	1.55	0.03	438.28	25.49
12	8.18	0.02	4.15	0.08	1.30	0.07	1.50	0.04	467.91	30.05
15	7.87	0.07	4.12	0.16	1.31	0.04	1.47	0.04	409.77	14.83
18	7.55	0.07	4.08	0.06	1.18	0.02	1.37	0.02	407.18	10.43

- lower than the detection limit

Supplementary Table S2

Ion abundance of metabolites among different straw extracts.

Index	Rt (min)	Molecular Weight (Da)	Formula	Ionization model	Metabolites	Class I	6 ESF	12 ESF	15 ESF	18 ESF	18 ES
mws0017	1.07	145.144	C7H19N3	[M+H]+	Spermidine	Alkaloids	69755	41314.33	50326.33	61230.67	32237.33
mws0018	1.1	202.197	C10H26N4	[M+H]+	Spermine	Alkaloids	30843667	27896667	27188000	26744000	33687000
pmb0490	2.77	234.123	C13H18N2O2	[M+H]+	N-p-Coumaroyl putrescine	Alkaloids	4945	10338.2	6147.533	7645.533	71524
pmb0501	1.14	130.111	C5H14N4	[M+H]+	Agmatine	Alkaloids	4129.267	7278.6	8617.033	10062.07	11757.67
pmp001255	5.26	313.131	C18H19NO4	[M+H]+	N-trans-feruloyltyramine	Alkaloids	5599.3	5557.667	5331.4	5722.867	71903.67
pmp001256	5.41	313.13	C18H19NO4	[M+H]+	N-cis-feruloyltyramine	Alkaloids	17610	13772	14679	14349	74198

		1									
mws0146	4.19	137.042	C7H7NO2	[M+H]+	Nicotinic Acid Methyl Ester(Methyl Nicotinate)	Alkaloids	116196.3	79739	45266	39587.67	72055
mws0191	1.28	117.07	C5H11NO2	[M+H]+	Betaine	Alkaloids	4116100	1811933	1487967	830006.7	3121533
mws0393	4.02	325.174	C20H25N2O2	[M]+	Quinine	Alkaloids	16436.67	4672.783	2990.833	2505.143	24387.67
mws1346	1.3	161.059	C6H11NO4	[M+H]+	DL-2-Aminoadipic acid	Alkaloids	7336.933	9256.4	10120.2	9450.4	8383.367
mws1383	4.96	242.071	C12H10N4O2	[M+H]+	Lumichrome	Alkaloids	58417	42243.67	48685.67	47520.67	44682
mws2218	3.64	194.071	C8H10N4O2	[M+H]+	Caffeine	Alkaloids	5310.7	9625.1	9037.5	13939.33	7609.5
pmb0037	1.95	165.105	C10H15NO	[M+H]+	Hordenine	Alkaloids	64045.33	81579.67	79562	79486.33	80276.67
pmb0374	2.39	135.049	C5H5N5	[M+H]+	Aminopurine	Alkaloids	2153900	1816067	1875300	1928600	1194400

pmb0484	1.24	103.091	C5H13NO	[M+H]+	Choline	Alkaloids	16573667	18043333	12611333	13801333	33165333
pmb1912	2.92	473.144	C20H23N7O7	[M+H]+	10-Formyl-THF	Alkaloids	2223067	165236.7	72364.67	56282	4327800
pmb2211	7.03	342.262	C19H38N2O3	[M+H]+	Cocamidopropyl betaine	Alkaloids	198970	202700	121701.3	100653	89468.67
pme1137	1.93	139.022	C6H5NO3	[M+H]+	6-Hydroxynicotinic acid	Alkaloids	54425667	4529267	1610367	1154917	65179000
pme1691	1.27	146.106	C7H16NO2	[M+H]+	Acetylcholine	Alkaloids	577166.7	646380	561313.3	534970	1653600
pme2268	1.29	137.042	C7H7NO2	[M+H]+	Trigonelline	Alkaloids	1349300	1031693	955410	755536.7	597490
pme2693	1.22	130.1	C6H14N2O	[M+H]+	N-Acetylputrescine	Alkaloids	934203.3	549733.3	645390	593136.7	2258367
pmp001214	3.44	310.147	C16H24NO5+	[M]+	Sinapine	Alkaloids	23958.3	2472.1	2981.367	3957.8	20853333
pmp001245	3.38	280.13	C15H22NO4+	[M]+	Feruloylcholine	Alkaloids	79679	24577	16899.	16809	188800

		8							33		
pmp001272	7.4	477.253	C23H44NO7P	[M+H]+	3-{(2-Aminoethoxy)(hydroxy)phosphoryl]oxy}-2-12-octadecadienoate	Alkaloids	1019633	849140	858876.7	877593.3	36623
pmp001277	7.76	453.253	C21H44NO7P	[M+H]+	3-{(2-Aminoethoxy)(hydroxy)phosphoryl]oxy}-2-hydroxypropyl palmitate	Alkaloids	18750.33	23786.33	20812	17941.67	142206.7
pmp001280	8.07	479.268	C23H46NO7P	[M+H]+	3-{[(2-Aminoethoxy)(hydroxy)phosphoryl]oxy}-2-hydroxypropyl-9,12-octadecenoate	Alkaloids	485926.7	675216.7	795903.3	875496.7	19927.67
pmp001287	2.42	119.067	C8H9N	[M+H]+	N-Benzylmethylene isomethylamine	Alkaloids	19823000	20808000	21229333	23441333	11288833
mws0005	3.22	160.091	C10H12N2	[M+H]+	Tryptamine	Alkaloids	33877.67	31608.67	33464	42120.67	20720
mws0098	5.49	161.042	C9H7NO2	[M-H]-	Indole-2-carboxylic acid	Alkaloids	6426.467	2737.8	2368.667	2249.467	155476.7

mws0102	4.82	161.042	C9H7NO2	[M-H]-	Indole-5-carboxylic acid	Alkaloids	2287900	293250	202100	155580	7560900
mws0103	4.99	145.047	C9H7NO	[M+H]+	Indole-3-carboxaldehyde	Alkaloids	20025000	6759533	5336633	4669767	16385000
mws0597	3.45	191.05	C10H9NO3	[M-H]-	5-Hydroxyindole-3-acetic acid	Alkaloids	261356.7	530793.3	550750	568790	558176.7
mws1320	5.27	161.076	C10H11NO	[M-H]-	Tryptophol	Alkaloids	18452.67	7751.867	3547.5	2803.267	701410
mws1417	4.89	161.042	C9H7NO2	[M-H]-	Indole-3-carboxylic acid	Alkaloids	3581667	464623.3	318110	245420	11986333
pmb1096	2.97	117.053	C8H7N	[M+H]+	Indole	Alkaloids	22443.33	15055	12922.33	12701.33	6073.867
pme2836	3.36	177.07	C10H11NO2	[M+H]+	5-Hydroxytryptophol	Alkaloids	408310	199200	197957.7	91544.67	417446.7
Qingke_Rfmb318-1-2	1.39	144.092	C7H14NO2+	[M]+	Proline betaine(ProBet)	Amino acids and derivativ	6145100	6297667	5183700	4912833	4368867

						es					
Qingke_Rfmb 320-1-3	1.39	143.08 5	C7H13NO2	[M+H]+	1,2-N-Methylpipecolic acid	Amino acids and derivativ es	141140 00	131980 00	121963 33	913560 0	958610 0
mws0001	1.24	132.04 6	C4H8N2O3	[M+H]+	L-Asparagine Anhydrous	Amino acids and derivativ es	23879. 67	27255. 33	30170	24235. 67	6530.3
mws0124	4.53	246.08 9	C13H14N2O3	[M+H]+	N-(3-Indolylacetyl)-L-alanine	Amino acids and derivativ es	19255. 33	4114.2 33	3597.6	3644.6	34262. 67
mws0216	1.26	131.05	C5H9NO3	[M+H]+	Trans-4-Hydroxy-L-proline	Amino acids and derivativ es	683670 00	618720 00	579333 33	511286 67	209003 33

mws0219	1.21	133.03	C4H7NO4	[M-H]-	L-Aspartic Acid	Amino acids and derivatives	257046.7	562630	563630	589543.3	433710
mws0221	1.27	240.012	C6H12N2O4S2	[M+H]+	L-(-)-Cystine	Amino acids and derivatives	33404.67	31939	42931.67	32366.33	34502.33
mws0227	1.7	131.085	C6H13NO2	[M+H]+	L-Leucine	Amino acids and derivatives	2304333	2948100	3258200	2947767	714073.3
mws0250	1.63	181.065	C9H11NO3	[M+H]+	L-(-)-Tyrosine	Amino acids and derivatives	7277067	4246967	5316267	5128667	2374800
mws0254	1.18	155.06	C6H9N3O2	[M+H]+	L-Histidine	Amino	130755	163226	179500	179350	107553

		1				acids and derivatives	.3	.7			.7
mws0256	1.34	117.07	C5H11NO2	[M+H]+	L-Valine	Amino acids and derivatives	45579333	36289333	32034333	26753333	43459000
mws0258	1.73	131.085	C6H13NO2	[M+H]+	L-Isoleucine	Amino acids and derivatives	2695267	3364200	3863567	3468567	830866.7
mws0260	1.23	174.1	C6H14N4O2	[M+H]+	L-(+)-Arginine	Amino acids and derivatives	3358200	3719433	4293400	4094100	7947667
mws0263	1.42	129.036	C5H7NO3	[M-H]-	L-Pyroglutamic acid	Amino acids and	67051.33	17351.33	12295.17	12715.67	151370

						derivatives					
mws0279	1.17	169.076	C7H11N3O2	[M+H]+	3-N-Methyl-L-histidine	Amino acids and derivatives	4781.633	7161.467	6609.367	7183.4	10268.77
mws0282	2.97	204.08	C11H12N2O2	[M-H]-	L-Tryptophan	Amino acids and derivatives	2512067	956026.7	849240	657473.3	654120
mws0340	2.85	144.035	C6H8O4	[M-H]-	2,3-Dimethylsuccinic acid	Amino acids and derivatives	262540	949766.7	1113700	1087767	84568.33
mws0520	3.13	223.074	C11H13NO4	[M+H]+	N-Acetyl-L-tyrosine	Amino acids and derivativ	386793.3	120280	79492	66089.33	1370367

						es					
mws0582	1.73	321.082	C11H19N3O6S	[M+H]+	S-(methyl)glutathione	Amino acids and derivatives	272223.3	38842.67	39081	44751	1314850
mws0629	2.99	280.092	C13H16N2O5	[M+H]+	Asp-phe	Amino acids and derivatives	6926833	6756567	6871267	6968467	1597767
mws0636	4.02	312.132	C18H20N2O3	[M+H]+	Phe-Phe	Amino acids and derivatives	2436400	2247567	2114967	1617833	78024.67
mws0712	1.78	131.05	C5H9NO3	[M-H]-	N-Propionylglycine	Amino acids and derivatives	557760	446276.7	299296.7	182550	242240

mws0715	3.66	264.098	C13H16N2O4	[M-H]-	Phenylacetyl-L-glutamine	Amino acids and derivatives	35774.67	42220.67	42566.33	40986	164026.7
mws0736	2.42	188.103	C8H16N2O3	[M+H]+	N-Glycyl-L-leucine	Amino acids and derivatives	37403000	48593667	50302333	48927000	8248333
mws0805	1.37	309.089	C11H19NO9	[M-H]-	N-Acetylneuraminic acid	Amino acids and derivatives	23638.67	32498	26052	32626.67	9349.133
mws0813	1.74	129.036	C5H7NO3	[M-H]-	5-Oxoproline	Amino acids and derivatives	32422	11851.33	8578.8	7526.067	19909.67
mws0875	1.28	178.03	C5H10N2O3	[M+H]+	Cys-Gly	Amino	7399.2	5501.9	12615.	7910.3	5450.7

		2	S			acids and derivatives	67		4	67	33
mws0923	3.07	199.032	C9H10ClNO2	[M+H]+	L-2-chlorophenylalanine	Amino acids and derivatives	11191000	7680133	8867800	9899333	15230333
mws1050	1.32	147.045	C5H9NO4	[M+H]+	O-Acetylserine	Amino acids and derivatives	22920.67	23750.33	17518.67	17140	11161
mws1570	1.29	131.05	C5H9NO3	[M+H]+	Cis-4-Hydroxy-D-proline	Amino acids and derivatives	497333.3	648300	694900	684863.3	1061540
mws1587	1.85	131.085	C6H13NO2	[M+H]+	α-Aminocaproic acid	Amino acids and	2460133	3102633	3532700	3108700	726003.3

						derivativ es					
mws4134	1.75	612.12 1	C20H32N6O1 2S2	[M-H]-	Oxidized Glutathione	Amino acids and derivativ es	10370. 17	13401. 33	10496. 23	9365.3	3377
mws4176	2.79	236.10 3	C12H16N2O3	[M+H]+	DL-Alanyl-DL-phenylalanine	Amino acids and derivativ es	526570 0	567110 0	620936 7	516013 3	61898. 67
mws5035	4.04	278.14 7	C15H22N2O3	[M+H]+	Leucylphenylalanine	Amino acids and derivativ es	109146 7	110883 3	119836 7	929836 .7	20633. 67
mws5037	2.9	202.11 8	C9H18N2O3	[M+H]+	Alanylleucine	Amino acids and derivativ	940216 .7	691050	665800	669963 .3	160216 7

						es					
mws5041	2.36	188.103	C8H16N2O3	[M+H]+	Glycylisoleucine	Amino acids and derivatives	5203833	3104933	2359100	2522200	1737667
mws5042	2.76	222.089	C11H14N2O3	[M+H]+	Glycylphenylalanine	Amino acids and derivatives	3141133	2533967	2606167	2188300	179076.7
pmb2591	4.33	246.089	C13H14N2O3	[M-H]-	Acetyltryptophan	Amino acids and derivatives	43114.33	9	9	9	126726.7
pme0006	1.29	115.056	C5H9NO2	[M+H]+	L-Proline	Amino acids and derivatives	146306.7	169683.3	180500	224340	17438.33

pme0008	1.26	175.084	C6H13N3O3	[M+H]+	L-Citrulline	Amino acids and derivatives	2943.567	4057.3	4495.367	5307.267	3548.633
pme0014	1.33	147.045	C5H9NO4	[M+H]+	L-Glutamic acid	Amino acids and derivatives	1332700	1698100	1389577	1086100	587103.3
pme0021	2.49	165.07	C9H11NO2	[M+H]+	L-Phenylalanine	Amino acids and derivatives	901356.7	939746.7	987100	1002870	469786.7
pme0026	1.18	146.095	C6H14N2O2	[M+H]+	L-(+)-Lysine	Amino acids and derivatives	4189833	5129267	4563567	4746667	1611933
pme0120	1.3	117.07	C5H11NO2	[M+H]+	5-Aminovaleric acid	Amino	153006	138823	128326	115716	211926

						acids and derivatives	.7	.3	.7	.7	.7
pme0122	1.52	188.103	C8H16N2O3	[M+H]+	N6-Acetyl-L-lysine	Amino acids and derivatives	178703.3	74137.67	69696.67	70151.33	231466.7
pme0124	1.29	172.075	C7H12N2O3	[M+H]+	Glycyl-L-proline	Amino acids and derivatives	51337	25083.33	23081	20019.33	32496.67
pme0137	1.67	188.069	C7H12N2O4	[M-H]-	N-α-Acetyl-L-glutamine	Amino acids and derivatives	3693567	2731100	2445633	2200067	1562533
pme0170	1.62	216.108	C8H16N4O3	[M+H]+	N-α-Acetyl-L-arginine	Amino acids and	674373.3	463963.3	367073.3	521723.3	1238033

						derivatives					
pme0172	3.22	159.079	C7H13NO3	[M+H]+	N-Isovaleroylglycine	Amino acids and derivatives	72560.67	9	9	9	203496.7
pme0181	1.22	169.076	C7H11N3O2	[M+H]+	1-Methylhistidine	Amino acids and derivatives	176380	75938.67	56982.67	51279.67	9646.7
pme0193	1.18	146.06	C5H10N2O3	[M+H]+	L-Glutamine	Amino acids and derivatives	4368067	4956433	4934800	4902633	2054867
pme0253	4	173.094	C8H15NO3	[M+H]+	N-Acetyl-L-leucine	Amino acids and derivativ	2154167	564956.7	599340	544770	5995500

						es					
pme0278	1.23	190.083	C7H14N2O4	[M+H]+	2,6-Diaminooimelic acid	Amino acids and derivatives	63852	59081.33	36083.67	32904.67	14244.67
pme1002	1.7	137.076	C8H11NO	[M+H]+	Tyramine	Amino acids and derivatives	2764933	62689.67	10347.23	11150.77	15355667
pme1210	1.68	149.042	C5H11NO2S	[M+H]+	L-Methionine	Amino acids and derivatives	363583.3	543826.7	745263.3	675526.7	9
pme1228	2.08	220.075	C11H12N2O3	[M+H]+	5-Hydroxy-L-tryptophan	Amino acids and derivatives	168376.7	151363.3	136946.7	143730	306203.3

pme1286	1.81	384.10 3	C14H20N6O5 S	[M+H]+	S-(5'-Adenosy)-L-homocysteine	Amino acids and derivativ es	261076 .7	165533 .3	143230	139206 .7	2482.5
pme2122	1.13	111.07 2	C5H9N3	[M+H]+	Histamine	Amino acids and derivativ es	249020 0	157690 0	146566 7	122330 0	286136 7
pme2566	1.64	218.07 8	C8H14N2O5	[M-H]-	(5-L-Glutamyl)-L-amino acid	Amino acids and derivativ es	23229. 33	50808. 33	80823. 33	62340	20495. 33
pme2602	1.69	185	C3H8NO6P	[M-H]-	O-Phospho-L-serine	Amino acids and derivativ es	16065. 6	62842. 67	84523. 33	75836	11482
pme2634	1.34	117.07	C5H11NO2	[M+H]+	Norvaline	Amino	485710	397140	351256	293320	468803

						acids and derivatives	00	00	67	00	33
pme2743	3.88	193.065	C10H11NO3	[M+H]+	N-Phenylacetylglycine	Amino acids and derivatives	696516.7	9	9	9	2377267
pme2758	1.28	163.039	C5H9NO5	[M-H]-	4-Hydroxy-L-glutamic acid	Amino acids and derivatives	5756.9	7633.9	7104.1	7014.167	22118.33
pme2773	1.27	222.055	C7H14N2O4S	[M+H]+	L-Cystathionine	Amino acids and derivatives	83455.67	95263.33	102781.3	98717.33	1649.813
pme2853	4.42	173.094	C8H15NO3	[M-H]-	Hexanoyl glycine	Amino acids and	35618.67	16051.33	17605.33	15510	681776.7

						derivatives					
pme2890	1.31	268.041	C8H16N2O4S2	[M+H]+	L-Homocystine	Amino acids and derivatives	859930	536103.3	522193.3	543263.3	187100
pme2914	1.9	162.044	C6H10O5	[M-H]-	3-Hydroxy-3-methylpentane-1,5-dioic acid	Amino acids and derivatives	7800333	5949633	6982467	4735333	3815600
pme3017	1.24	103.056	C4H9NO2	[M+H]+	2-Aminoisobutyric acid	Amino acids and derivatives	1824900	2086133	1520633	1687133	4028500
pme3033	1.25	103.056	C4H9NO2	[M+H]+	N,N-Dimethylglycine	Amino acids and derivativ	319326.7	348636.7	313840	299620	479253.3

						es					
pme3193	1.7	117.03 6	C4H7NO3	[M-H]-	N-Acetylglycine	Amino acids and derivativ es	31194. 67	8310.6 33	8052.3 33	6728.3 33	67879
pme3382	1.71	161.05 9	C6H11NO4	[M-H]-	N-Acetylthreonine	Amino acids and derivativ es	171603 .3	74873. 33	56232. 33	33685	64070. 67
pme3388	1.18	188.11 4	C7H16N4O2	[M+H]+	H-HomoArg-OH	Amino acids and derivativ es	200380	224750	236260	241623 .3	481766 .7
pmp001257	3.85	271.19 4	C15H29NO3	[M+H]+	Tridecanoyl glycine	Amino acids and derivativ es	294366 7	252576 7	291970 0	237163 3	356516 7

mws0057	4.16	450.096	C21H22O11	[M+H]+	Eriodictyol 7-O-glucoside	Flavonoids	22321	15956.33	21113.33	19270.83	19269
mws0463	6.13	302.067	C16H14O6	[M-H]-	Hesperetin	Flavonoids	553703.3	35797.67	9788.333	5914.2	1222433
pme0376	6.04	272.058	C15H12O5	[M-H]-	Naringenin	Flavonoids	14695.33	7935.633	9050.167	7793.467	6708.567
pmb0615	3.13	788.199	C34H44O21	[M+H]+	Hesperetin C-hexosyl-O-hexosyl-O-hexoside	Flavonoids	9	9	9	9	2155600
pmb2979	4.49	550.108	C25H26O14	[M-H]-	Hesperetin O-malonylhexoside	Flavonoids	2059.527	3445.033	2644.833	1857.733	93270
pme1598	4.31	464.111	C22H24O11	[M-H]-	Hesperetin 5-O-glucoside	Flavonoids	21542.33	13432.5	8406.3	8813.333	566420
mws0170	3.95	287.045	C15H11O6	[M]+	Cyanidin chloride	Flavonoids	206546.7	32419.33	12251.4	12315.27	160130
mws0175	3.24	493.112	C23H25O12	[M]+	Malvin 3-O-glucoside chloride	Flavonoids	465613.3	123808.3	81342.33	76615.33	9
mws0997	2.92	478.09	C22H22O12	[M+H]+	Petunidin 3-O-glucoside	Flavonoi	173763	54365	42654.	39278.	174300

		1				ds	.3		67	67	0
mws1048	3.18	448.082	C21H20O11	[M+H]+	Cyanidin 3-O-galactoside	Flavonoids	9	9	9	9	1362433
pmb0550	3.06	449.089	C21H21O11	[M]+	Cyanidin 3-O-glucoside(Kuromanin)	Flavonoids	9	9	9	9	993020
pmb2962	3.61	474.096	C23H22O11	[M-H]-	Pelargonidin O-acetylhexoside	Flavonoids	4705.567	2828.4	4250.5	3339.567	1719
pme0443	3.37	493.112	C23H25O12	[M]+	Malvidin 3-O-galactoside	Flavonoids	314336.7	77617.33	51335	43822.67	20124.33
pme0444	3.37	493.112	C23H25O12	[M]+	Malvidin 3-O-glucoside(Oenin)	Flavonoids	181546.7	40779.67	32412.33	25727.33	28031.67
pme1398	2.88	465.084	C21H21O12	[M]+	Delphinidin 3-O-glucoside(Mirtillin)	Flavonoids	9	9	9	9	2367967
mws0058	6.1	300.053	C16H12O6	[M-H]-	Diosmetin	Flavonoids	9	9	9	9	92189.33
mws1073	3.68	594.132	C27H30O15	[M+H]+	Apigenin 6,8-C-diglucoside	Flavonoids	19104.6	4379.267	8609.767	1443.967	36536.33

pmb0566	4.21	580.117	C26H28O15	[M+H]+	Luteolin O-hexosyl-O-pentoside	Flavonoids	3974.6	20851.67	22862.33	28801	1603.2
pmb0588	3.88	610.126	C27H30O16	[M+H]+	Luteolin 3',7-di-O-glucoside	Flavonoids	5244.733	3962.167	2942.067	2652.167	44637.67
pmb0620	3.28	786.184	C34H42O21	[M+H]+	Chrysoeriol 6-C-hexoside 8-C-hexoside-O-hexoside	Flavonoids	37008.67	55291.33	64003.33	64047.67	6312.267
pmb0691	3.56	756.175	C33H40O20	[M+H]+	Luteolin C-hexosyl-O-rhamnoside O-hexoside	Flavonoids	47714	115061.7	4277.367	3147.867	649.7167
pmb0713	3.95	654.149	C29H34O17	[M+H]+	Tricin 7-O-hexosyl-O-hexoside	Flavonoids	38565.67	10018.23	6332.567	5269.367	18135
pmb0716	4.61	654.149	C29H34O17	[M+H]+	Tricin di-O-hexoside	Flavonoids	23835.33	6693.433	3169.567	2933.367	27215.67
pmb0746	6.29	526.125	C27H26O11	[M+H]+	Tricin 4'-O-β-guaiacylglycerol	Flavonoids	9	9	9	9	83370.67
pmb2968	2.71	624.14	C28H32O16	[M-H]-	Chrysoeriol C-hexosyl-O-hexoside	Flavonoids	2097.233	9736.033	10857.87	12343	9
pmb3032	6.13	562.10	C26H26O14	[M-H]-	Tricin O-malonyl rhamnoside	Flavonoi	1651.2	2950.3	2991.8	2647.7	2907.2

		8				ds	33		33		33
pmb3039	6.44	704.112	C35H28O16	[M-H]-	Tricin O-oxalic acid O-coumaroyl shikimic acid	Flavonoids	54302.33	49643.33	50496.67	45182.67	46878
pmb3041	4.24	522.08	C23H22O14	[M-H]-	Tricin O-saccharic acid	Flavonoids	35653.67	13228.8	12116	16665.67	108156.7
pme2459	4.27	448.082	C21H20O11	[M+H]+	Luteolin 7-O-glucoside(Cynaroside)	Flavonoids	953396.7	131599.7	73930.67	63899	778720
pme3509	5.07	254.049	C15H10O4	[M+H]+	7,4'-Dihydroxyflavone	Flavonoids	21866	42867.33	33995.33	36279	5927.467
pmf0374	4.69	478.091	C22H22O12	[M-H]-	Isorhamnetin-3-O-glucoside	Flavonoids	233756.7	109170	64986.67	44441.33	5022067
pmn001668	3.68	416.128	C22H24O8	[M-H]-	Apigenin-3-O-α-L-rhamnoside	Flavonoids	10491.67	42770.67	47917.67	45463	51255
pmp000238	3.89	580.117	C26H28O15	[M+H]+	Luteolin 7-O-β-D-glucosyl-6-C-α-L-arabinose	Flavonoids	4424.533	12947.9	12043	18373	632.5933
pmp000579	4.87	462.096	C22H22O11	[M+H]+	Diosmetin-7-O-galactoside	Flavonoids	1975.2	2417.59	755.64	1670.067	32924.33

pmp000595	3.81	610.126	C27H30O16	[M+H]+	Luteolin 7,3'-di-O-β-D-Glucoside	Flavonoids	107086	19874.33	10343.6	9878.2	93323
GQ512001	3.18	772.17	C33H40O21	[M-H]-	Quercetin-O-rutinoside-hexose	Flavonoids	2209.687	1202.273	910.4133	1523.533	1342400
Li512117	4.39	610.126	C27H30O16	[M+H]+	Quercetin 3-O-rhanosylgalactoside	Flavonoids	11247.67	2098.2	1256.9	647.09	13539.67
mws0032	4.89	318.027	C15H10O8	[M+H]+	Myricetin	Flavonoids	1675.907	1197.267	730.06	1392.64	72339.67
mws0059	4.23	610.126	C27H30O16	[M-H]-	Rutin	Flavonoids	10743.67	9917.833	5425.7	10104.7	48758
mws0066	6.31	316.047	C16H12O7	[M-H]-	Isorhamnetin	Flavonoids	23449.33	7175.833	6399.067	5580.633	63808
mws0089	4.65	448.082	C21H20O11	[M-H]-	Kaempferol 7-O-glucosdie	Flavonoids	284953.3	65022.67	42937.33	37753.33	185723.3
mws0091	4.48	464.076	C21H20O12	[M-H]-	Isoquercitrin	Flavonoids	20061.33	8202.567	7727.533	8044.067	702643.3
mws1068	6.18	286.03	C15H10O6	[M-H]-	Kaempferol	Flavonoi	11697.	10413.	8415.3	6745.9	15454

		8				ds	33	13	33	67	
mws1329	4.5	464.076	C21H20O12	[M-H]-	Gossypitrin	Flavonoids	13850	10136.9	12498	7708.333	642546.7
mws2209	4.66	448.082	C21H20O11	[M-H]-	Astragalin	Flavonoids	301736.7	59783	38723.33	37586.33	145326.7
pmb3894	6.36	330.061	C17H14O7	[M-H]-	Di-O-methylquercetin	Flavonoids	30333000	25721667	24660667	23490333	35800000
pmn001583	4.06	610.126	C27H30O16	[M-H]-	Bioquercetin	Flavonoids	2386.6	1559.6	3241.433	1003.483	21180
pmn001641	4.73	476.076	C22H20O12	[M-H]-	Kaempferol 3-O-β-D-glucuronide	Flavonoids	28403.67	9	9	9	75160.67
pmp000596	3.37	626.12	C27H30O17	[M+H]+	Quercetin 3,7-bis-O-β-D-glucoside	Flavonoids	46635.33	13901.33	14151.67	9870.6	1844267
pmp001105	3.53	756.175	C33H40O20	[M+H]+	Kaempferol 3-neohesperidoside-7-glucoside	Flavonoids	149103.3	75709.33	77289.33	96082.67	348926.7
pmp001107	3.62	756.175	C33H40O20	[M+H]+	Kaempferol-3-rutinoside-7-glucoside	Flavonoids	139977.3	53046.33	75743	83777	313403.3

pmp001309	4.15	464.076	C21H20O12	[M+H]+	6-Hydroxykaempferol-7-O-glucoside	Flavonoids	116503.3	78353	66841	63673.33	1047563
pmp001310	3.82	626.12	C27H30O17	[M+H]+	6-Hydroxykaempferol-3,6-O-diglucoside	Flavonoids	566423.3	128990.7	83291.67	54249.33	18096333
pmp001311	3.38	626.12	C27H30O17	[M+H]+	6-Hydroxykaempferol-7,6-O-diglucoside	Flavonoids	30950	24617	14832.33	12657.37	1286667
pmp001312	3.13	788.164	C33H40O22	[M+H]+	6-Hydroxykaempferol-3,7,6-O-triglycoside	Flavonoids	4890.033	1640.8	600.9733	1800.867	10012967
mws1292	4.02	564.123	C26H28O14	[M+H]+	Isoschaftoside	Flavonoids	40336.67	17381	22792.67	11672.67	65340.67
pmb0618	3.26	626.155	C28H34O16	[M+H]+	8-C-Hexosyl-hesperetin O-hexoside	Flavonoids	9345.733	3241.41	4902.833	4787.667	573206.7
pmb0645	3.87	626.155	C28H34O16	[M+H]+	6-C-Hexosyl-hesperetin O-hexoside	Flavonoids	9	9	9	9	304333.3
pmb0660	4.19	756.158	C36H36O18	[M+H]+	C-Hexosyl-luteolin O-p-coumaroylhexoside	Flavonoids	34494.67	5445.467	1713.753	2070.533	34763.67
pmb0662	4.17	786.16	C37H38O19	[M+H]+	C-Hexosyl-luteolin O-	Flavonoi	42969.	15717.	11953.	6959.8	27783

		7			feruloylhexoside	ds	33	67	63		
pme3227	3.99	578.137	C27H30O14	[M+H]+	Vitexin 2''-O-β-L-rhamnoside	Flavonoids	9074.3	5103.133	6728.667	2994.133	4729.767
pmp000236	4.12	564.123	C26H28O14	[M+H]+	Apigenin 6-C-glucoside-8-C-xyloside (Vicenin 3)	Flavonoids	27579.67	10735.13	6326.767	5622.9	3423.9
pmp000411	4.07	564.123	C26H28O14	[M+H]+	Genistein 8-C-apiosyl(1 → 6)glucoside	Flavonoids	88850.3	2680.533	6419.367	1936.5	7922.9
mws0024	2.25	170.015	C7H6O5	[M-H]-	Gallic acid	Flavonoids	180900	1403227	1530000	1658700	110888.7
mws0049	2.84	306.061	C15H14O7	[M+H]+	(+)-Gallocatechin	Flavonoids	17997	51069	52138	58896.33	5044.3
mws0054	3.49	290.067	C15H14O6	[M-H]-	Catechin	Flavonoids	124596.7	283650	279403.3	278720	117137.7
mws0183	3.05	154.021	C7H6O4	[M-H]-	Protocatechuic acid	Flavonoids	781983.3	761573.3	624660	540113.3	94996667
pme0460	3.77	290.067	C15H14O6	[M+H]+	L-Epicatechin	Flavonoids	20727.67	16962	19482.33	20296.33	8042.733

pme2482	3.51	138.026	C7H6O3	[M+H]+	Protocatechuic aldehyde	Flavonoids	119446.7	41923	30843	32777.67	278126.7
pmn001416	4.72	452.093	C24H20O9	[M-H]-	Catechin-(7,8-bc)-4α-(3,4-dihydroxyphenyl)-dihydro-2-(3H)-pyranone	Flavonoids	87906	20573.67	15099.33	10404.3	65466.33
pmn001494	5.71	418.142	C22H26O8	[M-H]-	(+)-Syringaresinol	Lignans and Coumarins	2279.667	850.4633	420.1833	359.99	122320
mws0987	6.24	160.046	C10H8O2	[M+H]+	6-MethylCoumarin	Lignans and Coumarins	220983.3	67604.33	36358.67	25112.33	33341.67
mws1014	4.07	208.029	C10H8O5	[M+H]+	Fraxetin	Lignans and Coumarins	68268	38782	41709	35190	126046.7
pmb0382	4.34	338.067	C19H14O6	[M+H]+	O-Feruloyl 4-hydroxylcoumarin	Lignans and Coumari	57829.67	36238	36285	29874.33	658840

						ns					
pmb0890	9.45	354.251	C21H38O4	[M+H]+	MAG(18:2)	Lipids	194823.3	472346.7	461653.3	661436.7	2338.133
pmb1656	8.85	352.237	C21H36O4	[M+H]+	MAG(18:3)isomer4	Lipids	11018.93	19095.67	15499.67	22904.67	3539.4
pmb2444	9	352.237	C21H36O4	[M+H]+	MAG(18:3)isomer1	Lipids	53486.33	150910	144933.3	234486.7	14160.63
pmn001495	6.4	330.251	C19H38O4	[M-H]-	Hexadecanoic acid 2,3-dihydroxypropyl ester	Lipids	34565000	29548333	28838667	27541000	39963000
mws0120	1.26	257.103	C8H20NO6P	[M+H]+	Choline alfoscerate	Lipids	45212	51720.33	59210.33	54471	9
pmp001250	7.42	520.304	C26H51NO7P+	[M]+	PC(18:2)	Lipids	15196.67	21305.33	16295.67	18290.33	747733.3
pmp001251	7.24	520.304	C26H51NO7P+	[M]+	PC(18:2)isomer	Lipids	440103.3	326290	322583.3	325493.3	847056.7
pmb0767	6.3	301.272	C18H39NO2	[M+H]+	Sphinganine	Lipids	2213133	5002033	5187200	5204767	39491.67

pmb2221	7.32	317.266	C18H39NO3	[M+H]+	Phytosphingosine	Lipids	25228667	35643667	31004667	40717667	554733.3
pmp001264	6.5	273.243	C16H35NO2	[M+H]+	Hexadecylsphingosine	Lipids	8254967	10525067	10216433	9034200	8859167
mws0126	9.02	523.325	C26H54NO7P	[M+H]+	1-Stearoyl-sn-glycero-3-phosphocholine	Lipids	21634.33	59716.33	53228.33	40171.33	555463.3
pmb0854	6.94	517.282	C26H48NO7P	[M+H]+	LysoPC 18:3	Lipids	53445.33	36572.33	34481	35179.67	435403.3
pmb0855	7.74	495.296	C24H50NO7P	[M+H]+	LysoPC 16:0	Lipids	31292.67	56055.33	50534	45346.67	1374733
pmb0865	6.67	517.282	C26H48NO7P	[M+H]+	LysoPC 18:3(2n isomer)	Lipids	42861	32284.67	27333	28850	397363.3
pmb2319	7.2	481.282	C23H48NO7P	[M+H]+	LysoPC 15:0	Lipids	863.9167	1810.767	1361.553	1503.723	52621.67
pmb2406	8.29	509.311	C25H52NO7P	[M+H]+	LysoPC 17:0	Lipids	1144.467	2413.433	2180.067	2696.3	58877.33
pmd0132	7.57	495.29	C24H50NO7	[M+H]+	LysoPC 16:0(2n isomer)	Lipids	31739.	61372	55979	71710	280933

		6	P				33				.3
pmp001270	7.08	493.28<br>2	C24H48NO7<br>P	[M+H]+	LysoPC(16:2)	Lipids	5202.2<br>33	5991.1<br>33	5680.2	5062.4<br>33	36218
pmp001273	7.43	519.29<br>6	C26H50NO7<br>P	[M+H]+	LysoPC(18:2)	Lipids	14208	17280.<br>33	17931.<br>67	13616.<br>67	702543<br>.3
pmp001278	7.81	495.29<br>6	C24H50NO7<br>P	[M+H]+	LysoPC(16:1)	Lipids	33594.<br>33	58108.<br>67	49395	45587.<br>33	157526<br>7
pmp001281	8.12	521.31<br>1	C26H52NO7<br>P	[M+H]+	LysoPC(18:1)	Lipids	3232.2<br>67	9378	11230.<br>73	14046	150023<br>.3
pmp001286	8.95	523.32<br>5	C26H54NO7<br>P	[M+H]+	LysoPC(18:0)	Lipids	22193.<br>67	56827.<br>67	54130.<br>33	37525.<br>67	559850
mws0289	7.99	479.26<br>8	C23H46NO7<br>P	[M-H]-	LysoPE 18:1	Lipids	216620	274356<br>.7	374420	419896<br>.7	9081.9<br>67
pmb0856	7.8	479.26<br>8	C23H46NO7<br>P	[M+H]+	LysoPE 18:1(2n isomer)	Lipids	531143<br>.3	716013<br>.3	853500	910220	21891.<br>33
pmb0874	7.32	477.25<br>3	C23H44NO7<br>P	[M+H]+	LysoPE 18:2(2n isomer)	Lipids	26144	24629.<br>67	23783.<br>67	23450	15876

pmb0876	7.68	453.253	C21H44NO7P	[M+H]+	LysoPE 16:0	Lipids	19372.67	25159.67	21750.33	17732.33	141823.3
pmd0160	7.55	453.253	C21H44NO7P	[M-H]-	LysoPE 16:0(2n isomer)	Lipids	6011.367	7654.333	7641.7	9322.167	19231.67
Qingke_Rfmb087-1-1	6.51	312.208	C18H32O4	[M-H]-	13-Oxo-9-hydroxy-10-octadecenoic acid	Lipids	1744700	2131233	2260533	3287867	839896.7
Qingke_Rfmb089-2-3	6.48	314.222	C18H34O4	[M-H]-	9,10-Dihydroxy-12-octadecenoic acid	Lipids	67627.67	142546.7	158516.7	185413.3	27790.33
Qingke_Rfmb090-1-3	8.03	296.214	C18H32O3	[M-H]-	13-Hydroxy-9,11-octadecadienoic acid	Lipids	1397133	2046267	1970067	1644800	2619200
Qingke_Rfmb091-1-1	8.03	296.214	C18H32O3	[M-H]-	9-Hydroxy-10,12-octadecadienoic acid	Lipids	1409933	2064200	1948000	1616767	2610433
YC512117	8.68	276.19	C18H28O2	[M+H]+	Octadecatetraenoic acid	Lipids	62097.67	60840.33	35256.33	35249.67	2128.733
mws0119	9.85	228.19	C14H28O2	[M-H]-	Myristic Acid	Lipids	21287667	20889333	20987000	21792000	19676333
mws0359	10.3	242.20	C15H30O2	[M-H]-	Pentadecanoic Acid	Lipids	187580	298763	331636	357990	456756

	8	5					0	3	7	0	.7
mws0361	9.85	254.20 5	C16H30O2	[M-H]-	Palmitoleic Acid	Lipids	48531. 33	49913	31123	36113	53027. 33
mws0362	10.4 4	270.23 4	C17H34O2	[M-H]-	Margaric Acid	Lipids	119214 .7	216680	192643 .3	201560	75167. 33
mws0366	9.78	278.20 5	C18H30O2	[M-H]-	γ-Linolenic Acid	Lipids	360333 33	236580 00	205250 00	212870 00	271336 7
mws0367	9.56	278.20 5	C18H30O2	[M-H]-	α-Linolenic Acid	Lipids	6581.6	3975	2984.6 33	3155.6	4854.8 67
mws0371	9.81	328.21 9	C22H32O2	[M-H]-	Cis-4,7,10,13,16,19-Docosahexaenoic Acid(C22:6n3)	Lipids	17001. 67	20199	20751. 67	23811. 33	18602
mws0383	10.4 7	268.21 9	C17H32O2	[M-H]-	Cis-10-Heptadecenoic Acid	Lipids	554873 3	899843 3	908493 3	102051 67	948086 .7
mws0396	11.1 1	282.23 4	C18H34O2	[M-H]-	Elaidic Acid	Lipids	340496 67	371013 33	362786 67	363110 00	542940 0
mws0752	8.14	186.14	C11H22O2	[M-H]-	Undecylic Acid	Lipids	786526	680386	720806	758126	896030

		7					.7	.7	.7	.7	
mws0967	6.96	352.202	C20H32O5	[M-H]-	5(S),6(R)-Lipoxin A4	Lipids	16348	12569	15506	17274	18259.67
mws1355	6.19	272.214	C16H32O3	[M-H]-	16-Hydroxy hexadecanoic acid	Lipids	5685.233	6899.067	8266.4	10903.67	177133.3
mws1489	10.05	284.248	C18H36O2	[M-H]-	Stearic Acid	Lipids	81285333	75091333	74097000	83539000	67504667
mws1491	11.24	280.219	C18H32O2	[M-H]-	Linoleic acid	Lipids	67937	72970.67	48232	34056.33	42731.67
mws2623	10.9	282.234	C18H34O2	[M-H]-	11-Octadecanoic acid(Vaccenic acid)	Lipids	32476000	35773000	34175333	34217000	5191567
mws5045	7.03	216.156	C12H24O3	[M-H]-	12-Hydroxydodecanoic acid	Lipids	20922.33	16782	13931	14010.67	39191.67
pmb0889	7.91	278.205	C18H30O2	[M+H]+	Punicic acid	Lipids	4567567	4168067	3569600	2925533	3419900
pmb2640	8.62	200.162	C12H24O2	[M-H]-	Lauric acid	Lipids	10617.57	11353.33	8928.267	11133	11772

pmb2643	9	226.176	C14H26O2	[M-H]-	Myristoleic acid	Lipids	2586.6	2519.433	1891.033	2667.033	3832.567
pmb2778	7.92	296.214	C18H32O3	[M-H]-	9,10-EODE	Lipids	8535167	5678633	4804933	4208500	4975467
pmb2786	7.37	294.199	C18H30O3	[M-H]-	9-HOTrE	Lipids	928210	426883.3	299640	269416.7	3558333
pmb2787	8.12	294.199	C18H30O3	[M-H]-	9-KODE	Lipids	123953.3	180030	148726.7	192473.3	18342.33
pmb2792	6.77	294.199	C18H30O3	[M-H]-	13-HOTrE(r)	Lipids	91238	69124.33	86714	73138	118313.3
pmb2799	8.7	296.214	C18H32O3	[M-H]-	12,13-EODE	Lipids	12700.67	47435	55578.67	76925.33	8117.267
pmn001606	10.01	310.262	C20H38O2	[M-H]-	Eicosenoic acid	Lipids	262173.3	248170	241413.3	259343.3	283040
pmn001610	11.09	308.248	C20H36O2	[M-H]-	Eicosadienoic acid	Lipids	242283.3	160623.3	113639.7	134846.7	57251.67
pmn001686	6.64	288.20	C16H32O4	[M-H]-	10,16-Dihydroxy-palmitic acid	Lipids	62717.	56141.	47911.	42029.	17075

		8					67	33	67	33	
pmn001688	7.97	296.214	C18H32O3	[M-H]-	9S-Hydroxy-10E,12E-octadecadienoic acid	Lipids	1421200	2080567	1987600	1646433	2625600
pmn001689	6.35	312.208	C18H32O4	[M-H]-	9-Hydroxy-12-oxo-10-octadecenoic acid	Lipids	827156.7	479226.7	414776.7	331493.3	4970367
pmn001691	6.12	328.202	C18H32O5	[M-H]-	9,12,13-Trihydroxy-10,15-octadecadienoic acid	Lipids	3573667	1198467	783413.3	693116.7	7371833
pmn001694	6.41	330.217	C18H34O5	[M-H]-	9,10,13-Trihydroxy-11-octadecenoic acid	Lipids	32254667	28988000	27257667	26611667	37699667
mws0248	1.69	244.058	C9H12N2O6	[M-H]-	Uridine	Nucleotides and derivatives	1135567	1385633	1343700	1187153	2845000
mws0251	2.06	126.037	C5H6N2O2	[M+H]+	Thymine	Nucleotides and derivatives	412013.3	59590.67	33812	43044.33	1321233
mws0255	1.25	111.03	C4H5N3O	[M+H]+	Cytosine	Nucleotides and	347920	216686	206950	171240	504096

		8				derivatives	0	7	0	0	7
mws0572	1.63	125.052	C5H7N3O	[M+H]+	5-Methylcytosine	Nucleotides and derivatives	894156.7	379646.7	384870	364440	1002957
mws0609	1.87	345.033	C10H12N5O7P	[M-H]-	Guanosine 3',5'-cyclic monophosphate	Nucleotides and derivatives	23425.33	956516.7	2559567	1336467	21842667
mws0675	1.29	334.041	C11H15N2O8P	[M+H]+	β-Nicotinamide mononucleotide	Nucleotides and derivatives	18364.33	26504.67	23831.33	16897.27	2140.533
mws0724	1.98	299.072	C10H13N5O6	[M-H]-	8-Hydroxyguanosine	Nucleotides and derivatives	8597.833	17811.67	18983	21820.67	303896.7
mws0847	1.53	149.063	C6H7N5	[M+H]+	1-Methyladenine	Nucleotides and derivativ	1072520	898236.7	917956.7	935756.7	985906.7

						es					
mws0863	1.73	234.988	C5H9NaO7P	[M-Na]-	2-Deoxyribose 5-phosphate	Nucleotides and derivatives	118310	223500	182260	224506.7	144086.7
mws0872	2.81	281.098	C11H15N5O4	[M-H]-	1-Methyladenosine	Nucleotides and derivatives	147510	205970	197350	179240	228273.3
mws0874	1.33	347.047	C10H14N5O7P	[M-H]-	3'-Aenylic acid	Nucleotides and derivatives	8373.433	12654.67	17997.33	19482	104570
mws0884	2.34	329.038	C10H12N5O6P	[M-H]-	Cyclic AMP	Nucleotides and derivatives	445173.3	375050	368416.7	317643.3	982223.3
mws1715	2.45	251.089	C10H13N5O3	[M+H]+	Cordycepin	Nucleotides and derivativ	867023.3	125965.7	99506.33	93401.67	353313.3

						es					
pmb0530	1.64	663.08	C21H27N7O14P2	[M+H]+	Nicotinic acid adenine dinucleotide	Nucleotides and derivatives	610736.7	640296.7	782203.3	790013.3	6581.033
pmb0981	1.64	347.047	C10H14N5O7P	[M+H]+	Adenosine 5'-monophosphate	Nucleotides and derivatives	25322	95863.67	274930	196173.3	1702033
pmb0998	1.65	363.042	C10H14N5O8P	[M+H]+	Guanosine 5'-monophosphate	Nucleotides and derivatives	20787.33	53724	73494	60583.33	46140.67
pme0040	1.62	135.049	C5H5N5	[M+H]+	Adenine	Nucleotides and derivatives	1118903	627560	772333.3	730253.3	57911000
pme0166	2.48	166.042	C6H6N4O2	[M+H]+	1-Methylxanthine	Nucleotides and derivativ	67906.67	8829.467	1590.387	1976.733	72243.67

						es					
pme0183	1.35	151.043	C5H5N5O	[M+H]+	2-Hydroxy-6-aminopurine	Nucleotides and derivatives	219443.3	149126.7	146056.7	122806.7	742306.7
pme0230	2.38	267.084	C10H13N5O4	[M+H]+	Adenosine	Nucleotides and derivatives	11941000	13935000	14776667	15322000	7339633
pme0256	1.67	152.028	C5H4N4O2	[M-H]-	Xanthine	Nucleotides and derivatives	1264910	383560	350570	202383.3	6285533
pme0257	1.5	112.023	C4H4N2O2	[M-H]-	Uracil	Nucleotides and derivatives	406570	340146.7	324796.7	299943.3	411490
pme0264	2.58	242.078	C10H14N2O5	[M+H]+	Thymidine	Nucleotides and derivativ	4995100	249496.7	126473.3	123177.3	14656333

						es					
pme1109	1.65	151.043	C5H5N5O	[M+H]+	Guanine	Nucleotides and derivatives	454726.7	96175.33	111696.7	103776.3	5831467
pme1178	1.96	283.078	C10H13N5O5	[M+H]+	Guanosine	Nucleotides and derivatives	18108667	17345333	18042000	16995000	13818333
pme1184	2.18	267.084	C10H13N5O4	[M+H]+	Deoxyguanosine	Nucleotides and derivatives	28185667	5744100	4535100	4471633	4842233
pme1194	1.63	227.079	C9H13N3O4	[M+H]+	Deoxycytidine	Nucleotides and derivatives	7992400	1917333	1777967	1543903	3573167
pme1373	1.7	307.042	C9H14N3O7P	[M-H]-	2'-Deoxycytidine-5'-monophosphate	Nucleotides and derivativ	86933.33	49903.33	45059.33	41388.33	288770

						es					
pme1474	3.35	297.075	C11H15N5O3S	[M+H]+	5'-Deoxy-5'-(methylthio)adenosine	Nucleotides and derivatives	10319167	8178833	6484333	7108800	26932.67
pme3007	1.18	403.985	C9H14N2O12P2	[M-H]-	Uridine 5'-diphosphate	Nucleotides and derivatives	52866.67	128083.3	145433.3	146120	178263.3
pme3174	1.28	323.037	C9H14N3O8P	[M+H]+	Cytidine 5'-monophosphate(Cytidylic acid)	Nucleotides and derivatives	43136	83952.33	83104.67	68849.67	49648.67
pme3184	1.64	331.053	C10H14N5O6P	[M+H]+	2'-Deoxyadenosine-5'-monophosphate	Nucleotides and derivatives	41609.67	24779.67	31416.67	25552	196933.3
pme3188	1.73	324.021	C9H13N2O9P	[M-H]-	Uridine 5'-monophosphate	Nucleotides and derivativ	840310	3852900	5408900	4028067	1550267

						es					
pme3337	2.67	383.09	C14H17N5O8	[M+H]+	N6-Succinyl Adenosine	Nucleotides and derivatives	2366933	1253533	1007990	1079063	14097000
pme3732	1.32	243.073	C9H13N3O5	[M+H]+	Cytidine	Nucleotides and derivatives	16794667	13501333	12988000	10561867	9245233
pme3961	2.46	251.089	C10H13N5O3	[M+H]+	Deoxyadenosine	Nucleotides and derivatives	4917233	729493.3	559500	560060	2271433
pme3967	2.73	311.107	C12H17N5O5	[M+H]+	2-(Dimethylamino)guanosine	Nucleotides and derivatives	16665000	3035300	2296867	2103867	33445000
pme3968	1.8	165.057	C6H7N5O	[M+H]+	7-Methylguanine	Nucleotides and derivativ	399793.3	33999	26884	23015.33	2079233

						es					
mws0147	2.59	118.055	C5H10O3	[M-H]-	3-Hydroxy-3-methyl butyric acid	Organic acids	2625400	813410	557980	425530	4073300
mws0177	2.67	112.012	C5H4O3	[M-H]-	2-Furanoic acid	Organic acids	64062333	7613200	4200100	2720700	23828000
mws0192	1.81	118.021	C4H6O4	[M-H]-	Succinic acid	Organic acids	1093987	296236.7	166493.3	82597.67	792443.3
mws0206	2.09	104.041	C4H8O3	[M-H]-	(S)-2-Hydroxybutanoicacid	Organic acids	558900	233676.7	180423.3	110976.7	125340
mws0208	3.13	146.049	C6H10O4	[M-H]-	Adipic Acid	Organic acids	20376000	18926333	18804667	17146000	18437333
mws0237	4.93	188.093	C9H16O4	[M-H]-	Anchoic Acid	Organic acids	2502767	599430	444710	363340	6318333
mws0242	4.32	174.078	C8H14O4	[M-H]-	SubericAcid	Organic acids	2303033	707700	568386.7	474223.3	4400267
mws0274	4.37	166.055	C9H10O3	[M-H]-	Phenyllactate(PLA)	Organic acids	1355140	1410767	723923.3	400796.7	1546900

mws0275	1.47	134.01 5	C4H6O5	[M-H]-	L-(-)-Malic acid	Organic acids	723996 7	263873 3	168196 7	129776 7	771420 0
mws0277	1.63	192.05 3	C7H12O6	[M-H]-	Kinic acid	Organic acids	100485 7	77767	43615	69639. 33	287466 .7
mws0281	1.34	192.01 8	C6H8O7	[M-H]-	Citric Acid	Organic acids	261470 00	168760 0	775786 .7	670410	352740 0
mws0341	4.01	132.07	C6H12O3	[M-H]-	(S)-(-)-2-Hydroxyisocaproic acid	Organic acids	699093 3	247153 3	210670 0	204506 7	108958 33
mws0344	1.45	166.03 8	C5H10O6	[M-H]-	D-Xylonic acid	Organic acids	566383 .3	239796 .7	180280	184093 .3	519790
mws0345	1.49	129.07	C6H11NO2	[M-H]-	Pipecolinic acid	Organic acids	71869	12574. 67	10847. 43	12934	172380
mws0376	1.61	116.00 6	C4H4O4	[M-H]-	Fumaric acid	Organic acids	521800 0	198800 0	187833 3	145526 7	102337 33
mws0425	2.15	130.02 1	C5H6O4	[M-H]-	Citraconic acid	Organic acids	722896 7	153946 7	160860 0	163630 0	309763 33
mws0470	1.82	118.02	C4H6O4	[M-H]-	Methylmalonic acid	Organic	105617	285733	183640	68365.	784250

		1				acids	7	.3		33	
mws0473	2.58	132.035	C5H8O4	[M-H]-	2-Methylsuccinic acid	Organic acids	56968333	97576667	1.01E+08	98825000	29927333
mws0474	6.62	230.136	C12H22O4	[M-H]-	Dodecanedioic aicd	Organic acids	24168.33	7134.7	4596.333	4351.6	274900
mws0489	3.07	150.026	C8H6O3	[M-H]-	Benzoylformic acid	Organic acids	945003.3	394763.3	347026.7	294456.7	911376.7
mws0497	4.37	166.055	C9H10O3	[M-H]-	L-(-)-3-Phenyllactic acid	Organic acids	1271083	1340900	703470	383736.7	1449600
mws0567	1.27	145.076	C5H11N3O2	[M+H]+	4-Guanidinobutyric acid	Organic acids	11531667	14852333	13611333	11982333	26349333
mws0574	2.05	104.041	C4H8O3	[M-H]-	α-Hydroxyisobutyric acid	Organic acids	740900	294640	244726.7	152570	173800
mws0576	1.89	104.041	C4H8O3	[M-H]-	3-Hydroxybutyrate	Organic acids	695486.7	157523.3	93416.67	65511.67	573946.7
mws0596	3.17	153.036	C7H7NO3	[M+H]+	3-Hydroxyanthranilic acid	Organic acids	5836600	14844667	17341000	19253667	161973.3

mws0612	4.4	196.06 4	C10H12O4	[M-H]-	3,4-Dimethoxyphenyl acetic acid	Organic acids	53880	16407. 1	14411. 33	11869. 97	142316 .7
mws0639	3.14	154.02 1	C7H6O4	[M-H]-	2,3-Dihydroxybenzoic Acid	Organic acids	407396 .7	563650	465806 .7	466163 .3	720413 33
mws0640	2.82	118.05 5	C5H10O3	[M-H]-	2-Hydroxy-2-methylbutyric acid	Organic acids	37767	46443. 33	46438	40691. 33	40288. 67
mws0671	1.25	119.05	C4H9NO3	[M+H]+	L-Homoserine	Organic acids	10146. 17	11164.2 3	12609. 67	10708. 4	17312. 33
mws0823	2.49	116.04 1	C5H8O3	[M-H]-	3-Methyl-2-Oxobutanoic acid	Organic acids	150110 0	338436 .7	294250	232166 .7	566540 0
mws0924	3.13	146.04 9	C6H10O4	[M-H]-	2-Methylglutaric acid	Organic acids	210633 33	196083 33	191593 33	174280 00	194120 00
mws0972	3.13	132.07	C6H12O3	[M-H]-	5-Hydroxyhexanoic acid	Organic acids	308796 7	429523 3	355110 0	289296 7	175096 7
mws1167	1.37	132.00 1	C4H4O5	[M-H]-	Oxaloacetic acid	Organic acids	77323	88823. 33	113193 .3	98793. 33	84837. 33
mws1189	1.26	194.03	C6H10O7	[M-H]-	D-Galacturonic acid(Gal A)	Organic	174093	709063	470053	427123	46980.

		3				acids	33	3	3	3	33
mws1213	1.26	194.03<br>3	C6H10O7	[M-H]-	Aldehydo-D-galacturonate	Organic acids	184010<br>00	732703<br>3	542273<br>3	451820<br>0	47669
mws1214	1.32	546.08	C18H26O19	[M-H]-	D-Galactopyranuronate	Organic acids	42318.<br>67	62026	49644	61547	5153.9
mws2125	1.59	205.93<br>1	C3H4KO6P	[M-K]-	Phosphoenolpyruvic acid	Organic acids	692763<br>.3	586436<br>.7	637003<br>.3	593613<br>.3	270613<br>.3
mws2184	5.97	208.06<br>4	C11H12O4	[M-H]-	Ethyl caffeate	Organic acids	63082.<br>67	8187.6	5118.2<br>33	3210.3<br>33	157163<br>.3
mws2628	1.27	117.07	C5H11NO2	[M+H]+	N-Methyl-4-aminobutyric acid	Organic acids	32752.<br>67	24840.<br>33	17709	19019	14637.<br>33
pmb3099	2.11	154.03	C4H11O4P	[M-H]-	Diethyl phosphate	Organic acids	204646<br>.7	205846<br>.7	176956<br>.7	178903<br>.3	241733<br>.3
pme0266	5.52	202.10<br>7	C10H18O4	[M-H]-	Sebacate	Organic acids	151950	304523<br>.3	239703<br>.3	224150	98523.<br>33
pme0271	1.76	116.00<br>6	C4H4O4	[M-H]-	Maleic acid	Organic acids	38567	13530.<br>33	14194.<br>33	10128.<br>83	69181

pme0274	1.5	131.085	C6H13NO2	[M+H]+	6-Aminocaproic acid	Organic acids	5431500	5161600	4465900	3534600	2873600
pme0282	3.48	166.021	C8H6O4	[M-H]-	Phthalic acid	Organic acids	7585467	6209433	6763633	5494000	8910867
pme0295	2.09	145.065	C6H11NO3	[M+H]+	4-Acetamidobutyric acid	Organic acids	11420333	6389967	4153300	3270133	11275433
pme2049	2.07	104.041	C4H8O3	[M-H]-	2-Hydroxybutanoic acid	Organic acids	2708633	1055303	830436.7	521070	696046.7
pme3011	1.27	103.056	C4H9NO2	[M+H]+	γ-Aminobutyric acid	Organic acids	128640	200920	671673.3	625990	601000
pme3154	1.8	148.064	C6H12O4	[M-H]-	(Rs)-Mevalonic acid	Organic acids	425730	63732.33	75071.67	39947	440770
pmn001682	3.77	180.035	C9H8O4	[M-H]-	Sorbic acid	Organic acids	157896.7	16902.67	9117.467	8294.9	397240
pmn001578	10.72	256.219	C16H32O2	[M-H]-	Hexadecanoic acid	Organic acids	23164667	27575000	27304333	27079000	12398300
CMLN000394	1.66	437.04	C12H23NO10	[M-H]-	4-Methylthio-3-hydroxybutyl	Others	6407.6	6337.3	7260.9	4841.5	5332.4

		8	S3		glucosinolate		67				
CMLN000620	1.99	451.065	C13H25NO10S3	[M-H]-	3-Hydroxy-5-(methylthio)pentyl thioglucoside	Others	19350.33	27841.67	24728.5	12858.57	13792.67
CMLN001029	2.65	544.012	C16H20N2O13S3	[M-H]-	1-Sulfo-indol-3-ylmethyl thioglucoside (Glucobrassicin-1-sulfate)	Others	4260.133	5790.767	4542.2	4795.567	9
CMLN001085	2.73	405.076	C12H23NO10S2	[M-H]-	2-Hydroxy-2-Methylbutyl Glucosinolate (Glucocleomin)	Others	149310	117040	97033.33	74386	27731.67
CMLN001498	3.37	419.038	C12H21NO9S3	[M-H]-	4-Methylthio-3-butenyl thioglucoside (Dehydroerucin)	Others	22107.33	19736.67	17754	14062.33	32839.33
CMLN001810	3.87	435.069	C13H25NO9S3	[M-H]-	5-Methylthiopentyl Glucosinolate	Others	23513.67	15682.33	11677.3	9513.533	38319.33
Cmyp001406	2.51	465.08	C14H27NO10S3	[M-H]-	3-Hydroxy-6-(methylthio)hexyl glucosinolate	Others	11508.67	9132.5	7817.867	7119.067	37644
Cmyp001649	2.74	407.038	C11H21NO9S3	[M-H]-	3-Methylthiopropyl Glucosinolate	Others	239706.7	86154.67	60730.67	31559.33	150283.3
Cmyp001733	2.71	375.06	C11H21NO9S	[M-H]-	1-Methylpropyl Glucosinolate	Others	73725	40179	37702.	25375	67799

		6	2						33		
Cmyp002073	3.06	387.06 6	C12H21NO9 S2	[M-H]-	3-Methyl-3-butenyl glucosinolate	Others	16314. 47	14547	10458. 93	8673.8 67	13675. 67
Lmyn002727	4.38	448.06 1	C16H20N2O9 S2	[M-H]-	3-Indolylmethyl glucosinolate	Others	224003 3	173560	58667. 33	31143. 33	445143 3
Lmyn002971	4.71	403.09 7	C13H25NO9 S2	[M-H]-	3-methylpentyl glucosinolate	Others	203156 .7	152460	127743 .3	111713. 3	300073 .3
Lmyn003359	5.19	421.05 3	C12H23NO9 S3	[M-H]-	4-methylthiobutyl glucosinolate	Others	169130 0	138510 0	110837 3	845433 .3	221023 3
Lmyn003825	5.76	435.03 3	C12H21NO10 S3	[M-H]-	4-Methylsufinyl-3-Butenyl Glucosinolate	Others	280326 67	169463 33	111779 33	779650 0	420423 33
mws0757	1.77	359.01 7	C10H17NO9 S2	[M-H]-	(-)-Sinigrin hydrate	Others	60930. 67	50473. 33	63228. 33	29927. 33	31024. 67
pmn001434	3.16	409.03 2	C14H19NO9 S2	[M-H]-	Glucotropaeolin	Others	24173. 33	24422. 33	30861	20334. 33	21951
pmn001673	2.17	373.03 2	C11H19NO9S 2	[M-H]-	Gluconapin	Others	491780	499643 .3	609666 .7	426376 .7	423723

pmn001677	4.3	478.05	C17H22N2O10S2	[M-H]-	Neoglucobrassicin	Others	146350	38066.33	37029	31023.67	158813.3
YC512118	10.19	281.249	C18H35NO	[M+H]+	Octadecenoic amide	Others	166103.3	153130	151903.3	110825	120373.3
mws0491	2.89	121.082	C8H11N	[M+H]+	Phenethylamine	Others	41645000	24752333	17817667	14279000	48824000
mws0983	9.8	325.272	C20H39NO2	[M+H]+	N-Oleoylethanolamine	Others	18190	30464	36384	42562	10789.2
mws1429	4.47	224.127	C13H20O3	[M-H]-	Vomifoliol	Others	532760	184096.7	152393.3	119397	767710
pmn001380	5.03	188.093	C9H16O4	[M-H]-	Eucommiol	Others	7127000	1766400	1302667	1092167	17763333
pmn001592	6.66	444.184	C27H28N2O4	[M-H]-	Aurantiamide acetate	Others	2082.9	675.0967	1305.473	745.4167	7555.967
pmp000509	3.25	126.026	C6H6O3	[M+H]+	Maltol	Others	68069.33	72554.33	66487.67	83616.67	2218667
pmp001259	4.38	525.29	C28H47NO8	[M+H]+	5,8,11,14-Pentadecanoamide	Others	47488.	31674.	38307.	36031	30496.

		6					33	33	67		67
pmp001266	7.18	279.235	C18H33NO	[M+H]+	Crucigasterin E	Others	26102.33	39167.67	31139.67	24572	48762
mws0198	1.26	180.053	C6H12O6	[M-H]-	D-(+)-Glucose	Others	46964	104691.7	100167.7	106759.7	95546.33
mws0213	1.27	152.058	C5H12O5	[M-H]-	Ribitol	Others	634426.7	1063500	1093800	1075120	612463.3
mws0214	1.24	182.067	C6H14O6	[M-H]-	D-Sorbitol	Others	9658.867	19795	18881.33	20251.33	43948.33
mws0264	1.22	342.096	C12H22O11	[M-H]-	D-(+)-TrehaloseAnhydrous	Others	2391.333	6412	5730.8	5897.167	3330.967
mws0437	1.23	152.058	C5H12O5	[M-H]-	D-Arabitol	Others	53669.67	102453.3	101322.3	113130	48398
mws0438	1.26	152.058	C5H12O5	[M-H]-	L-Arabitol	Others	36136.33	99707.67	143550	124976.7	102611.3
mws0866	1.19	260.016	C6H13O9P	[M-H]-	D-Glucose 6-phosphate	Others	583960	811883.3	670283.3	585426.7	94428.33

mws1080	1.25	342.096	C12H22O11	[M-H]-	Galactinol	Others	17344	38073	40523	46118	86908
mws1090	1.28	260.016	C6H13O9P	[M-H]-	Glucose-1-phosphate	Others	592230	813396.7	667773.3	567920	93499
mws1155	1.28	182.067	C6H14O6	[M-H]-	Mannitol	Others	10175.37	15001	12629.33	15906.33	19060
mws1333	1.23	342.096	C12H22O11	[M-H]-	Melibiose	Others	33686.67	64430.33	69636	82160.67	6276.9
mws1499	1.33	150.044	C5H10O5	[M-H]-	D-(-)-Arabinose	Others	45697	39660	46937	44321.33	77834.67
mws1593	1.27	666.184	C24H42O21	[M-H]-	Maltotetraose	Others	3879.967	8515.167	8617.267	7117.6	6335.9
mws2523	1.19	422.06	C12H23O14P	[M-H]-	Trehalose 6-phosphate	Others	33289.33	48919.33	43203	36490	28702
mws4170	1.32	180.053	C6H12O6	[M-H]-	D-Glucose	Others	100798.3	308583.3	401050	385016.7	108181.3
mws5038	1.32	342.09	C12H22O11	[M-H]-	Isomaltulose	Others	46847	75706	92691.	93957.	70473

		6							67	67	
mws5040	1.36	364.078	C12H21O11Na	[M+H]+	Turanose	Others	42594.33	35382	32599.67	18726.33	318846.7
pmb3081	1.59	289.99	C6H11PO11	[M-H]-	Glucarate O-Phosphoric acid	Others	148166.7	80084	89031.67	61491.33	88935.67
pme0513	1.27	152.058	C5H12O5	[M-H]-	Xylitol	Others	42213	121510	247226.7	226893.3	245983.3
pme0516	1.24	180.053	C6H12O6	[M-H]-	Inositol	Others	6993.9	6107.8	4371	6390.967	3891.633
pme0519	1.28	342.096	C12H22O11	[M-H]-	D-(+)-Sucrose	Others	1479.6	2971.767	3038.767	2946	1582.067
pme0534	1.2	196.047	C6H12O7	[M-H]-	Gluconic acid	Others	351850	326680	262146.7	269140	380310
pme2237	1.25	182.067	C6H14O6	[M-H]-	Dulcitol	Others	68199.67	96796	104735.7	110522	92209.67
pme3705	1.22	194.033	C6H10O7	[M-H]-	D-Glucoronic acid	Others	18640000	7672233	5646400	4811500	47983

mws0133	1.99	122.043	C6H6N2O	[M+H]+	Nicotinamide	Others	1929633	2146000	1946200	1994767	11859333
mws0232	3.71	376.12	C17H20N4O6	[M+H]+	Riboflavin	Others	1451133	1064360	1216600	1161067	427456.7
mws1337	2.78	219.097	C9H17NO5	[M+H]+	D-Pantothenic Acid	Others	7930133	10599267	11090000	11533333	8358133
pme0490	1.61	123.027	C6H5NO2	[M+H]+	Nicotinic acid	Others	6923067	5196967	5280167	4672000	4081500
pmf0608	4.65	162.026	C9H6O3	[M-H]-	Umbelliferone	Phenolic acids	377193.3	34744	14744	9088.267	488310
mws0009	5.14	178.055	C10H10O3	[M+H]+	Coniferaldehyde	Phenolic acids	43904.33	30383	34118.33	26808.33	1055067
mws0014	4.53	194.049	C10H10O4	[M-H]-	Ferulic acid	Phenolic acids	63182	33049.33	25816.67	21943	5363533
mws0027	3.88	198.044	C9H10O5	[M-H]-	Syringic acid	Phenolic acids	162736.7	341060	319740	333133.3	13515333
mws0028	3.79	168.03	C8H8O4	[M-H]-	Vanillic acid	Phenolic	312093	787100	859966	104526	292900

		5				acids	.3		.7	0	00
mws0117	3.62	182.049	C9H10O4	[M-H]-	Homovanillic acid	Phenolic acids	115364.3	911700	1073400	1351633	80415.67
mws0145	4.59	152.041	C8H8O3	[M-H]-	2-Methoxybenzoic acid	Phenolic acids	12112.2	826.79	875.2033	863.1733	75758.67
mws0178	3.24	354.079	C16H18O9	[M-H]-	Chlorogenic acid	Phenolic acids	204543.3	78303	39222.67	49676.67	4812233
mws0179	4.17	368.093	C17H20O9	[M-H]-	Chlorogenic acid methyl ester	Phenolic acids	9	9	9	9	2255867
mws0180	3.07	154.021	C7H6O4	[M-H]-	2,5-Dihydroxybenzoic acid	Phenolic acids	275843.3	258276.7	226796.7	199943.3	57163333
mws0182	3.75	152.041	C8H8O3	[M-H]-	p-Hydroxyphenyl acetic acid	Phenolic acids	582933.3	133236.7	95516.33	66228	1712400
mws0444	1.84	153.036	C7H7NO3	[M-H]-	3-Aminosalicylic acid	Phenolic acids	1675033	320300	275646.7	202916.7	2544233
mws0458	4.47	152.041	C8H8O3	[M-H]-	Vanillin	Phenolic acids	1240500	2239167	2441933	2944500	31189333

mws0467	4.34	166.055	C9H10O3	[M-H]-	3-(4-Hydroxyphenyl)-propionic acid	Phenolic acids	2236300	2341067	1226230	690203.3	2546833
mws0628	4.19	122.032	C7H6O2	[M-H]-	4-Hydroxybenzaldehyde	Phenolic acids	3962800	783333.3	701246.7	647726.7	30568667
mws0748	2.71	354.079	C16H18O9	[M-H]-	1-Caffeoylquinic acid	Phenolic acids	66283.67	33378.67	20341.67	20352	1104300
mws0749	3.55	138.026	C7H6O3	[M-H]-	4-Hydroxybenzoic acid	Phenolic acids	13213300	1759300	1368233	1159490	61641000
mws0885	3.35	154.021	C7H6O4	[M-H]-	2,4-Dihydroxy benzoic acid	Phenolic acids	1651733	1172533	988673.3	806260	76312000
mws0921	4	150.061	C9H10O2	[M-H]-	p-Coumaryl alcohol	Phenolic acids	3159.667	9	9	9	243856.7
mws1024	4.75	148.046	C9H8O2	[M-H]-	p-Coumaraldehyde	Phenolic acids	12171.67	2819.133	1609.567	1761.267	264446.7
mws1078	4.51	137.042	C7H7NO2	[M+H]+	Anthranilic Acid	Phenolic acids	574740	406910	348166.7	343943.3	2707700
mws1336	3.24	137.04	C7H7NO2	[M+H]+	4-Aminobenzoic acid	Phenolic	26471.	12189.	12367.	11293.	191370

		2				acids	33	6	67	87	
mws1350	4.6	182.04 9	C9H10O4	[M-H]-	Syringic Aldehyde	Phenolic acids	331076 .7	346640	321246 .7	320333 .3	103271 00
mws1354	4.59	194.04 9	C10H10O4	[M-H]-	Trans-ferulic acid	Phenolic acids	56442. 67	33137	26106. 33	18555. 67	551130 0
mws1358	3.73	110.03 2	C6H6O2	[M-H]-	Pyrocatechol	Phenolic acids	3887.9	5453.8 67	5610.8 33	4495.1 67	74573. 67
mws2108	3.42	354.07 9	C16H18O9	[M-H]-	Cryptochlorogenic acid	Phenolic acids	42077. 33	30668. 33	32430	39974	826963 .3
mws2212	3.64	180.03 5	C9H8O4	[M-H]-	Caffeic acid	Phenolic acids	77074	103602	85807. 33	79855. 67	198780
mws2213	5.72	148.04 6	C9H8O2	[M-H]-	Cinnamic acid	Phenolic acids	4180.1 33	650.6	650.9	421.03	53596. 67
mws4085	4.51	224.05 8	C11H12O5	[M-H]-	Sinapic acid	Phenolic acids	8711.5 67	2712.5	2545.2 33	1911.0 33	31722. 67
mws4193	4	168.07	C9H12O3	[M-H]-	Homovanillic alcohol	Phenolic acids	84402	21873. 33	13399	9606.2 67	137200

pmb0423	3.69	194.049	C10H10O4	[M-H]-	Hydroxy-methoxycinnamate	Phenolic acids	687113.3	58361	17869	9704.5	710793.3
pmb0752	3.49	368.093	C17H20O9	[M+H]+	3-O-Feruloyl quinic acid	Phenolic acids	2955	2487.367	1936.277	2499.967	2089033
pmb1587	7.33	206.118	C13H18O2	[M+H]+	4-Hydroxy-3,5-diisopropylbenzaldehyde	Phenolic acids	260090	118006.7	79462.67	54083.67	199900
pmb2871	2.4	316.065	C13H16O9	[M-H]-	2,5-Dihydroxy benzoic acid O-hexside	Phenolic acids	338653.3	140406.7	135576.7	95199.33	14278667
pmb3068	3.34	338.085	C16H18O8	[M-H]-	1-O-p-Coumaroylquinic acid	Phenolic acids	196663.3	8316.367	11779.63	15263.67	1271700
pmb3074	3.96	338.085	C16H18O8	[M-H]-	3-O-p-Coumaroylquinic acid	Phenolic acids	580463.3	72485.33	86175.67	112933.3	3820833
pmb3107	2.84	360.088	C15H20O10	[M-H]-	Syringic acid O-glucoside	Phenolic acids	178290	31980.67	22768	13028.33	3230600
pme0281	3.5	166.021	C8H6O4	[M-H]-	Terephthalic acid	Phenolic acids	17004000	14616000	15614667	12885000	19131667
pme1439	4.29	164.04	C9H8O3	[M+H]+	p-Coumaric acid	Phenolic	96557	66703.	46022	46659.	60330.

		1				acids		33		67	33
pme1816	3.08	354.07<br>9	C16H18O9	[M-H]-	Neochlorogenic acid(5-O-Caffeoylquinic acid)	Phenolic acids	84255.<br>67	58147.<br>33	53138	51260.<br>33	147317<br>7
pme3437	4.94	212.05<br>8	C10H12O5	[M+H]+	Eudesmic acid(3,4,5-trimethoxybenzoic acid)	Phenolic acids	92872.<br>33	35562.<br>67	26754.<br>67	26979	274433<br>.3
pme3443	4.93	208.06<br>4	C11H12O4	[M-H]-	Sinapinaldehyde	Phenolic acids	84748.<br>33	20630.<br>33	16894	16641.<br>67	157983<br>33
pmf0284	5.15	178.05<br>5	C10H10O3	[M-H]-	Riboprine	Phenolic acids	163653<br>.3	85528.<br>33	86213.<br>33	86831.<br>67	284480<br>00
pmn001367	2.39	316.06<br>5	C13H16O9	[M-H]-	Protocatechuic acid 4-glucoside	Phenolic acids	421286<br>.7	201766<br>.7	211803<br>.3	181396<br>.7	202013<br>33
pmn001421	3.82	338.08<br>5	C16H18O8	[M-H]-	3-O-(E)-p-Coumaroyl quinic acid	Phenolic acids	143333<br>3	355103<br>.3	431066<br>.7	559536<br>.7	840166<br>7
pmn001515	2.62	326.05	C14H14O9	[M-H]-	5-O-Galloyl-(-)-shikimic acid	Phenolic acids	20919.<br>67	104041<br>.3	133540	149253<br>.3	9
pmn001519	4.63	336.03<br>6	C15H12O9	[M-H]-	Galloyl Methyl gallate	Phenolic acids	103823	117216<br>.7	106243<br>.3	89126	99733.<br>33

pmn001627	1.92	332.05 9	C13H16O10	[M-H]-	Glucogallin	Phenolic acids	30011. 67	28502. 67	20507. 33	20218. 67	284116 7
pmn001695	3.96	372.08 8	C16H20O10	[M-H]-	Trihydroxycinnamoylquinic acid	Phenolic acids	229110 0	122230 0	818110	607893 .3	227143 3
pmn001710	4.18	522.11 4	C24H26O13	[M-H]-	Rosmarinyl Glucoside	Phenolic acids	46335. 33	16897. 67	19771. 67	14347	24879. 67
pmn001738	3.77	404.11 1	C17H24O11	[M-H]-	Oleoside 11-methyl ester	Phenolic acids	831260	304026 .7	188030	109215	735963 .3
pmp000086	4.08	268.08 1	C13H16O6	[M+H]+	1-Feruloyl-sn-glycerol	Phenolic acids	103530 7	86613. 33	36633	31101. 33	148400 0
pmp000087	3.88	268.08 1	C13H16O6	[M+H]+	2-Feruloyl-sn-glycerol	Phenolic acids	606626 .7	65437	29554. 33	19987. 47	859363 .3
pmp000232	3.36	338.08 5	C16H18O8	[M+H]+	3-p-Coumaroylquinic acid	Phenolic acids	427910	1099.0 97	2799.6	1145.9 7	363463 3
pmp001285	8.71	148.01 2	C8H4O3	[M+H]+	Phthalic anhydride	Phenolic acids	252783 3	409106 7	190210 0	196613 3	177530 0
pmp000274	3.87	578.12	C30H26O12	[M+H]+	Procyanidin B3	Tannins	14536.	82555.	103687	89766	10097.

							97	67	.3		47
mws4053	8.21	456.32 9	C30H48O3	[M-H]-	Ursolic acid	Terpenoi ds	277660	85847. 67	85590	161803 .3	4333.7
pmp000437	4.55	442.34 9	C30H50O2	[M+H]+	Myricadiol	Terpenoi ds	4011.8 67	8342.2 67	8571.0 33	11363. 33	464.93 33

Supplementary Table S3

Variable importance in projection and fold change of differentially regulated metabolites between the pairwise comparison of 18 day's straw extracts.

Index	VIP	Fold_Change	Log2FC	Type	KEGG
CMLN001029	1.103294	532.8407	9.057561	up	--
CMLN001085	1.063042	2.682349	1.423497	up	--
CMLN001498	1.098618	0.428216	-1.22359	down	--
CMLN001810	1.059342	0.24827	-2.01002	down	ko00966,ko01110,ko01210
Cmyp001406	1.094406	0.189116	-2.40266	down	--
Cmyp001649	1.090567	0.209999	-2.25155	down	ko00966,ko01110,ko01210
Cmyp001733	1.037687	0.374268	-1.41786	down	ko00966,ko01110,ko01210
GQ512001	1.102064	0.001135	-9.78318	down	--
Li512117	1.077598	0.047792	-4.38708	down	--
Lmyn002727	1.094428	0.006996	-7.1592	down	ko00380,ko00966,ko01100,ko01110,ko01210
Lmyn002971	1.081704	0.372287	-1.42551	down	--
Lmyn003359	1.092965	0.382509	-1.38644	down	ko00966,ko01110,ko01210
Lmyn003825	1.094098	0.185444	-2.43094	down	--
Qingke_Rfmb087-1-1	1.084072	3.914609	1.968868	up	--
Qingke_Rfmb089-2-3	1.093961	6.671864	2.73809	up	--
YC512117	1.077421	16.55899	4.049542	up	ko00592,ko01110
mws0001	1.033929	3.711264	1.891911	up	ko00250,ko00460,ko00970,ko01100,ko01110,ko01230
mws0005	1.013912	2.032851	1.023504	up	ko00380,ko00901,ko01100,ko01110
mws0009	1.102263	0.025409	-5.29851	down	ko00940,ko01100,ko01110
mws0014	1.102338	0.004091	-7.93328	down	ko00940,ko01100,ko01110

mws0024	1.099156	14.95825	3.902869	up	--
mws0027	1.102857	0.024649	-5.34235	down	--
mws0028	1.10354	0.035687	-4.80847	down	--
mws0032	1.086071	0.019251	-5.69889	down	ko00941,ko00944,ko01110
mws0049	1.081172	11.67582	3.545452	up	ko00941,ko01110
mws0054	1.047688	2.379423	1.250611	up	ko00941,ko01110
mws0058	1.10416	9.76E-05	-13.3224	down	--
mws0059	1.065296	0.207242	-2.27061	down	--
mws0066	1.079612	0.08746	-3.51524	down	--
mws0089	1.062585	0.203277	-2.29848	down	--
mws0091	1.101692	0.011448	-6.44872	down	--
mws0098	1.101813	0.014468	-6.11097	down	--
mws0102	1.102378	0.020577	-5.60283	down	--
mws0103	1.054976	0.285003	-1.81095	down	--
mws0117	1.103291	16.80808	4.071083	up	ko00350,ko01100
mws0120	1.102722	6052.333	12.56328	up	--
mws0124	1.06854	0.106372	-3.23281	down	--
mws0126	1.097357	0.07232	-3.78945	down	--
mws0133	1.090111	0.168202	-2.57173	down	ko00760,ko01100
mws0145	1.102646	0.011394	-6.45562	down	--
mws0147	1.096819	0.104468	-3.25887	down	--
mws0170	1.050268	0.076908	-3.70072	down	--
mws0175	1.103807	8512.815	13.05542	up	--
mws0177	1.071534	0.114181	-3.13061	down	--
mws0178	1.099395	0.010323	-6.59799	down	ko00940,ko00941,ko00945,ko01110

mws0179	1.103822	3.99E-06	-17.9353	down	--
mws0180	1.096495	0.003498	-8.15936	down	ko00350,ko01100
mws0182	1.097471	0.038676	-4.69243	down	ko00350,ko00360,ko01100
mws0183	1.102735	0.005686	-7.45847	down	ko00400,ko01100,ko01110
mws0191	1.092747	0.265897	-1.91106	down	ko00260,ko01100,ko02010
mws0192	1.085217	0.104232	-3.26213	down	ko00020,ko00190,ko00250,ko00310,ko00350,ko00360,ko00620,ko00630,ko00640,ko00650,ko00760,ko00920,ko01100,ko01110,ko01200
mws0216	1.065263	2.446309	1.290606	up	ko00330,ko01100,ko02010
mws0227	1.082084	4.128101	2.045478	up	ko00280,ko00290,ko00966,ko00970,ko01100,ko01110,ko01210,ko01230,ko02010
mws0237	1.103745	0.057506	-4.12015	down	--
mws0242	1.102574	0.107771	-3.21395	down	--
mws0248	1.023185	0.417277	-1.26092	down	ko00240,ko01100,ko02010
mws0251	1.103915	0.032579	-4.93992	down	ko00240,ko01100
mws0255	1.070491	0.339697	-1.55768	down	ko00240,ko01100
mws0258	1.081416	4.174637	2.061651	up	ko00280,ko00290,ko00460,ko00960,ko00966,ko00970,ko01100,ko01110,ko01210,ko01230,ko02010
mws0263	1.102306	0.084004	-3.5734	down	--
mws0274	1.067893	0.259097	-1.94844	down	--
mws0275	1.099585	0.168231	-2.57149	down	ko00650,ko01100
mws0277	1.069978	0.242252	-2.04542	down	ko00400,ko01100
mws0281	1.096849	0.190058	-2.39549	down	ko00020,ko00250,ko00630,ko01100,ko01110,ko01200,ko01210,ko01230
mws0289	1.103703	46.23411	5.530886	up	--
mws0340	1.101207	12.86258	3.685108	up	--
mws0341	1.08751	0.187693	-2.41356	down	--

mws0344	1.063133	0.354169	-1.49749	down	--
mws0345	1.103951	0.075032	-3.73635	down	--
mws0359	1.087029	7.837652	2.970422	up	--
mws0366	1.098862	7.845235	2.971817	up	--
mws0376	1.09213	0.142203	-2.81398	down	ko00020,ko00190,ko00220,ko00250,ko00350,ko00360,ko00620,ko00650,ko00760,ko01100,ko01110,ko01200
mws0383	1.071336	10.76396	3.428137	up	--
mws0393	1.018403	0.102722	-3.28319	down	--
mws0396	1.089799	6.687848	2.741542	up	--
mws0425	1.100529	0.052824	-4.24266	down	ko00290,ko00660,ko01100,ko01210
mws0437	1.092181	2.337493	1.224962	up	ko00040,ko01100
mws0444	1.098627	0.079756	-3.64827	down	--
mws0458	1.103359	0.094407	-3.40496	down	ko00999,ko01100,ko01110
mws0463	1.092443	0.004838	-7.69136	down	ko00941
mws0467	1.066104	0.271005	-1.88361	down	--
mws0470	1.096841	0.087173	-3.51998	down	ko00240,ko00280,ko00640,ko01100
mws0473	1.092551	3.302165	1.723412	up	--
mws0474	1.102477	0.01583	-5.98122	down	--
mws0489	1.090133	0.32309	-1.62999	down	ko00360,ko01100
mws0491	1.092458	0.292459	-1.7737	down	ko00360,ko01100
mws0497	1.068451	0.264719	-1.91747	down	--
mws0520	1.09924	0.048227	-4.374	down	--
mws0567	1.074219	0.454749	-1.13686	down	ko00330,ko01100
mws0572	1.070398	0.363366	-1.46051	down	ko00240,ko01100
mws0576	1.095827	0.114142	-3.13109	down	--

mws0582	1.084349	0.034035	-4.87683	down	--
mws0596	1.103636	118.8694	6.893233	up	ko00380,ko01100
mws0609	1.094068	0.061186	-4.03065	down	ko00230,ko01100
mws0612	1.099346	0.083405	-3.58372	down	--
mws0628	1.10344	0.021189	-5.56052	down	ko01100
mws0629	1.093577	4.361379	2.124785	up	--
mws0636	1.101965	20.7349	4.373989	up	--
mws0639	1.101397	0.006471	-7.27185	down	ko01110
mws0675	1.056759	7.893952	2.980748	up	ko00760,ko01100
mws0715	1.095206	0.249874	-2.00073	down	ko00360
mws0724	1.074775	0.071803	-3.79981	down	--
mws0736	1.101575	5.931744	2.568456	up	--
mws0748	1.09369	0.01843	-5.76182	down	--
mws0749	1.103268	0.01881	-5.73233	down	ko00130,ko00790,ko01100,ko01110
mws0805	1.053976	3.489807	1.803147	up	--
mws0813	1.046483	0.378011	-1.4035	down	ko00480,ko01100
mws0823	1.10334	0.04098	-4.60895	down	ko00280,ko00290,ko00770,ko00966,ko01100,ko01110,ko01210,ko01230
mws0866	1.100669	6.199693	2.632197	up	ko00500,ko00562,ko00998,ko01100
mws0874	1.08898	0.186306	-2.42426	down	ko00230,ko01100
mws0885	1.102543	0.010565	-6.56452	down	--
mws0921	1.104184	3.69E-05	-14.7257	down	ko00940,ko01100,ko01110
mws0983	1.064849	3.944871	1.979978	up	--
mws0997	1.102874	0.022535	-5.47168	down	--
mws1014	1.046851	0.279182	-1.84072	down	--
mws1024	1.103157	0.00666	-7.23022	down	--

mws1048	1.104185	6.61E-06	-17.2078	down	--
mws1068	1.040945	0.436519	-1.19588	down	ko00941,ko00944,ko01100,ko01110
mws1073	1.088313	0.039521	-4.66122	down	--
mws1078	1.100822	0.127024	-2.97683	down	ko00380,ko00400,ko00998,ko01058,ko01100,ko01110,ko01230
mws1090	1.100123	6.074076	2.602665	up	--
mws1189	1.099726	90.91535	6.506452	up	ko00040,ko00053,ko00520,ko01100,ko02010
mws1213	1.101177	94.78277	6.566553	up	--
mws1214	1.090905	11.94183	3.577952	up	--
mws1292	1.015353	0.178643	-2.48485	down	--
mws1320	1.102689	0.003997	-7.96701	down	--
mws1329	1.102677	0.011997	-6.38124	down	--
mws1333	1.096542	13.08937	3.710324	up	ko00052,ko01100,ko02010
mws1336	1.092662	0.059016	-4.08275	down	ko00790,ko01100
mws1350	1.102936	0.031019	-5.01072	down	--
mws1354	1.101026	0.003367	-8.21439	down	--
mws1355	1.102279	0.061556	-4.02195	down	--
mws1358	1.095806	0.060278	-4.05222	down	ko01100
mws1417	1.102151	0.020475	-5.60999	down	--
mws1429	1.089505	0.155524	-2.68479	down	--
mws1587	1.086769	4.281936	2.098263	up	--
mws1715	1.036142	0.264359	-1.91943	down	--
mws2108	1.091361	0.048338	-4.37069	down	--
mws2125	1.070944	2.193585	1.133291	up	--
mws2184	1.101642	0.020427	-5.6134	down	--
mws2209	1.032772	0.258633	-1.95102	down	--

mws2213	1.099463	0.007856	-6.99208	down	ko00130,ko00360,ko00940,ko00999,ko01100,ko01110
mws2623	1.086261	6.590881	2.720471	up	--
mws4053	1.093573	37.33607	5.222498	up	--
mws4085	1.09002	0.060242	-4.05309	down	ko00940,ko01100,ko01110
mws4170	1.081233	3.558994	1.831469	up	--
mws4176	1.102539	83.36421	6.381356	up	--
mws4193	1.099809	0.070017	-3.83616	down	--
mws5035	1.102508	45.06405	5.493905	up	--
mws5037	1.086555	0.418161	-1.25787	down	--
mws5040	1.093885	0.058731	-4.08972	down	--
mws5042	1.101963	12.21991	3.611161	up	--
mws5045	1.065743	0.357491	-1.48402	down	--
pmb0382	1.101239	0.045344	-4.46295	down	--
pmb0423	1.095311	0.013653	-6.19463	down	--
pmb0484	1.082917	0.416137	-1.26487	down	ko00260,ko00564,ko01100,ko02010
pmb0490	1.090363	0.106895	-3.22574	down	ko00330,ko01100
pmb0530	1.096116	120.044	6.907419	up	ko00190,ko00730,ko00760,ko01100
pmb0550	1.104177	9.06E-06	-16.7515	down	ko00942
pmb0566	1.094587	17.9647	4.167093	up	--
pmb0588	1.084494	0.059415	-4.07302	down	--
pmb0615	1.104173	4.18E-06	-17.8697	down	--
pmb0618	1.083608	0.008352	-6.90359	down	--
pmb0620	1.078975	10.14654	3.342916	up	--
pmb0645	1.104178	2.96E-05	-15.0454	down	--
pmb0660	1.099897	0.05956	-4.06951	down	--

pmb0662	1.054639	0.250506	-1.99708	down	--
pmb0691	1.007597	4.844984	2.276492	up	--
pmb0713	1.059755	0.290563	-1.78308	down	--
pmb0716	1.088294	0.107782	-3.21381	down	--
pmb0746	1.101393	0.000108	-13.1773	down	--
pmb0752	1.101095	0.001197	-9.70671	down	--
pmb0767	1.097361	131.794	7.042141	up	--
pmb0854	1.088109	0.080798	-3.62954	down	--
pmb0855	1.099834	0.032986	-4.92201	down	--
pmb0856	1.10194	41.57901	5.377784	up	--
pmb0865	1.09079	0.072604	-3.78382	down	--
pmb0876	1.0963	0.125031	-2.99964	down	--
pmb0890	1.102685	282.8909	8.144102	up	--
pmb0981	1.066543	0.115258	-3.11706	down	ko00230,ko00908,ko01100,ko01110
pmb1096	1.00345	2.091145	1.064293	up	ko00380,ko00400,ko00402,ko01100,ko01110
pmb1587	1.080155	0.270554	-1.88601	down	--
pmb1656	1.076502	6.471342	2.694065	up	--
pmb1912	1.101427	0.013005	-6.26482	down	ko00670,ko00970,ko01100,ko01200
pmb2221	1.097984	73.40043	6.197717	up	ko00600,ko01100
pmb2319	1.093016	0.028576	-5.12905	down	--
pmb2406	1.098455	0.045795	-4.44866	down	--
pmb2444	1.092531	16.55905	4.049548	up	--
pmb2591	1.104065	7.1E-05	-13.7814	down	--
pmb2786	1.084572	0.075714	-3.72329	down	--
pmb2787	1.094716	10.49339	3.391409	up	--

pmb2799	1.043801	9.476753	3.244393	up	--
pmb2871	1.098105	0.006667	-7.22869	down	--
pmb2968	1.103956	1371.444	10.42148	up	--
pmb2979	1.097916	0.019918	-5.6498	down	--
pmb3041	1.08065	0.154088	-2.69817	down	--
pmb3068	1.101715	0.012003	-6.38051	down	--
pmb3074	1.102183	0.029557	-5.08034	down	ko00940,ko00941,ko00945,ko01110
pmb3107	1.10096	0.004033	-7.95401	down	--
pmd0132	1.071015	0.255256	-1.96998	down	--
pmd0160	1.01381	0.48473	-1.04475	down	--
pme0006	1.101547	12.86476	3.685353	up	ko00330,ko00332,ko00970,ko01100,ko01110,ko01230,ko02010
pme0021	1.077461	2.134735	1.094057	up	ko00360,ko00400,ko00460,ko00940,ko00960,ko00966,ko00970,ko01100,ko01110,ko01210,ko01230,ko02010
pme0026	1.091424	2.944704	1.558123	up	ko00300,ko00310,ko00780,ko00960,ko00970,ko01100,ko01110,ko01210,ko01230,ko02010
pme0040	1.103317	0.01261	-6.3093	down	ko00230,ko00908,ko01100
pme0122	1.091312	0.303073	-1.72226	down	ko00310,ko01100
pme0166	1.095144	0.027362	-5.19168	down	ko00232,ko01100,ko01110
pme0170	1.039173	0.421413	-1.24669	down	--
pme0172	1.102884	4.42E-05	-14.4647	down	--
pme0181	1.085466	5.315773	2.410279	up	ko00340,ko01100
pme0183	1.099118	0.165439	-2.59563	down	--
pme0193	1.097287	2.385864	1.254512	up	ko00220,ko00230,ko00240,ko00250,ko00630,ko00910,ko00970,ko01100,ko01230,ko02010
pme0230	1.090441	2.08757	1.061825	up	ko00230,ko01100,ko02010

pme0253	1.095796	0.090863	-3.46016	down	--
pme0256	1.100128	0.032198	-4.95687	down	ko00230,ko00232,ko01100,ko01110
pme0264	1.101962	0.008404	-6.89464	down	ko00240,ko01100
pme0266	1.101004	2.275096	1.185927	up	--
pme0271	1.096396	0.146411	-2.77191	down	ko00350,ko00650,ko00760,ko01100
pme0295	1.074032	0.290023	-1.78576	down	ko00330,ko01100
pme0460	1.025925	2.523562	1.335461	up	ko00941,ko01100,ko01110
pme1002	1.101646	0.000726	-10.4274	down	ko00350,ko00950,ko01100,ko01110
pme1109	1.098544	0.017796	-5.81231	down	ko00230,ko01100
pme1137	1.099077	0.017719	-5.81855	down	ko00760,ko01100
pme1210	1.104137	75058.52	16.19573	up	ko00270,ko00966,ko00970,ko01100,ko01110,ko01210,ko01230
pme1228	1.082634	0.469394	-1.09113	down	ko00380,ko01100
pme1286	1.102269	56.07519	5.809291	up	ko00270,ko01100,ko01230
pme1373	1.036027	0.143326	-2.80262	down	ko00240,ko01100
pme1398	1.104122	3.8E-06	-18.0053	down	ko00942
pme1474	1.103056	263.9471	8.044105	up	ko00270,ko00908,ko01100
pme1598	1.102613	0.01556	-6.00604	down	--
pme1691	1.087029	0.323518	-1.62808	down	--
pme1816	1.087646	0.034796	-4.84494	down	--
pme2122	1.051987	0.427523	-1.22593	down	ko00340,ko01100,ko01110
pme2459	1.075354	0.082056	-3.60724	down	ko00944
pme2482	1.103878	0.117852	-3.08496	down	ko00950
pme2566	1.040982	3.041668	1.604863	up	ko00480,ko01100
pme2602	1.100128	6.604773	2.723509	up	ko00260,ko00270,ko00970,ko00998,ko00999,ko01100,ko01200,ko01230
pme2693	1.055788	0.26264	-1.92884	down	ko00330,ko01100

pme2743	1.103604	3.79E-06	-18.0109	down	ko00360
pme2758	1.093513	0.31712	-1.6569	down	--
pme2773	1.090987	59.83546	5.902929	up	ko00260,ko00270,ko01100,ko01110,ko01230
pme2836	1.015023	0.219297	-2.18904	down	--
pme2853	1.091954	0.022749	-5.45803	down	--
pme3017	1.082677	0.418799	-1.25567	down	--
pme3154	1.098719	0.09063	-3.46387	down	--
pme3184	1.063546	0.129749	-2.9462	down	ko00230,ko01100
pme3188	1.042149	2.598306	1.377571	up	ko00240,ko01100
pme3193	1.084753	0.099122	-3.33464	down	--
pme3337	1.094781	0.076546	-3.70754	down	--
pme3437	1.096289	0.098308	-3.34655	down	--
pme3443	1.103384	0.001053	-9.89076	down	--
pme3509	1.081323	6.12049	2.613647	up	ko00941,ko00943
pme3705	1.097414	100.2751	6.64782	up	ko00040,ko00053,ko00520,ko00562,ko01100
pme3961	1.054723	0.246567	-2.01995	down	ko00230,ko01100,ko02010
pme3967	1.097846	0.062905	-3.99068	down	--
pme3968	1.095157	0.011069	-6.49731	down	--
pmf0284	1.103951	0.003052	-8.35589	down	--
pmf0374	1.098453	0.008849	-6.82024	down	--
pmf0608	1.092601	0.018612	-5.74765	down	ko00940,ko01110
pmn001367	1.102922	0.008979	-6.79916	down	--
pmn001380	1.103673	0.061484	-4.02364	down	--
pmn001416	1.102351	0.158926	-2.65357	down	--
pmn001421	1.104145	0.066598	-3.90837	down	--

pmn001494	1.090345	0.002943	-8.40849	down	--
pmn001515	1.103718	16583.7	14.01748	up	--
pmn001583	1.062314	0.047379	-4.39961	down	--
pmn001610	1.073913	2.355332	1.23593	up	ko01040
pmn001627	1.102597	0.007116	-7.13465	down	--
pmn001641	1.104105	0.00012	-13.0278	down	--
pmn001677	1.077533	0.195347	-2.35589	down	--
pmn001682	1.070761	0.020881	-5.58164	down	--
pmn001686	1.085818	2.461454	1.299511	up	--
pmn001689	1.080381	0.066694	-3.9063	down	--
pmn001691	1.099709	0.094022	-3.41085	down	--
pmn001695	1.100565	0.267625	-1.90171	down	--
pmn001738	1.098642	0.148397	-2.75246	down	--
pmp000086	1.095917	0.020958	-5.57637	down	--
pmp000087	1.083718	0.023258	-5.4261	down	--
pmp000232	1.096983	0.000315	-11.631	down	--
pmp000238	1.074666	29.04394	4.860165	up	--
pmp000274	1.091711	8.889953	3.152176	up	--
pmp000437	1.031887	24.44078	4.611218	up	--
pmp000509	1.099055	0.037688	-4.72976	down	--
pmp000579	1.072894	0.050724	-4.30118	down	--
pmp000595	1.090427	0.10585	-3.23991	down	--
pmp000596	1.102636	0.005352	-7.54569	down	--
pmp001105	1.078596	0.275366	-1.86058	down	--
pmp001107	1.04754	0.267314	-1.90339	down	--

pmp001214	1.096026	0.00019	-12.3633	down	ko00940
pmp001245	1.098145	0.089031	-3.48955	down	--
pmp001250	1.088613	0.024461	-5.35337	down	--
pmp001251	1.02097	0.384264	-1.37983	down	--
pmp001255	1.041626	0.079591	-3.65126	down	--
pmp001270	1.094409	0.139777	-2.8388	down	--
pmp001272	1.102759	23.9629	4.582731	up	--
pmp001273	1.089831	0.019382	-5.68914	down	--
pmp001277	1.095705	0.126166	-2.9866	down	--
pmp001278	1.09904	0.028939	-5.11082	down	--
pmp001280	1.103354	43.93373	5.457257	up	--
pmp001281	1.074148	0.093625	-3.41696	down	--
pmp001286	1.098293	0.067028	-3.89909	down	--
pmp001287	1.070294	2.076506	1.054158	up	--
pmp001309	1.103423	0.060782	-4.0402	down	--
pmp001310	1.103612	0.002998	-8.38188	down	--
pmp001311	1.101949	0.009837	-6.66752	down	--
pmp001312	1.103869	0.00018	-12.4409	down	--

Supplementary Table S4

Development toxicity, mutagenicity, 50% lethal (LC_{50}, mg/L) and growth inhibition (IGC_{50}, mg/L) concentration of sphingosines on nontarget organisms.

Sphingosines	Development toxicity	Mutagenicity	*Fathead minnow* LC_{96-50}	*Daphnia magna* LC_{48-50}	*Tetrahymena pyriformis* IGC_{48-50}	Oral rat LC_{50}
DHS	Non-toxicant	Negative	0.79	4.65	1.66	8469.37
PHS	Non-toxicant	Negative	1.86	25.87	4.93	11700.68
SPH	Non-toxicant	Negative	0.38	3.67	1.83	9741.48
APH	Non-toxicant	Negative	2.31	12.26	2.09	8735.12

Supplementary Table S5

Information of primers used in RT-qPCR for *A. carterae*.

Gene description	Primer name	Primer sequence (5'-3')	Product size (bp)
18S rRNA	18S-F	GAAGAACACGACTCTCAATG	140
	18S-R	CTGACTTGTAGGCTGTAGG	
High affinity nitrate transporter	HANT-F	CCACCATCTCGTTCTTCTT	163
	HANT-R	TTCTCCGTCTTCAAGTTCTT	
Nitrite transporter	NIT-F	CTTCTCCTCTGCCTTGTAA	166
	NIT-R	GGATGTGTTGGTAGATTGG	
Ammonium transporter	AMT-F	CACTCGGAGCACAGATTG	167
	AMT-R	GACATACGCCTTGGATGG	
Phosphate transporter	PST-F	TTGGTATGCTTCCTCCTGTA	130
	PST-R	CGGCAAGTGGATTGTCAA	
Alkaline phosphatase	ALP-F	GTCTGCTGTCAACTACTCA	152
	ALP-R	TGTCACACTGGTTCTTCTC	
Heat shock protein - 90	HSP90-F	ACAAGGTAAGCGGAGTAGA	145
	HSP90-R	TCGGAATGACCAAGAATGAA	
Photosystem I P700 chlorophyll a apoprotein A1	psaA-F	GGCAGGTGGCTCTATTAG	161
	psaA-R	AGTGAGACGCAAGGAATG	
The core protein of photosystem I processes	psaB-F	ACAGCGAGGTCATTAGAAC	142
	psaB-R	TATTGTCCTTGTGGCAGTC	.
Photosystem II protein D1	psbA-F	TTGACCGAATCTGTAACCTT	161
	psbA-R	CTGAGCACAACATCCTTATG	
Rhodopsin	RHO-F	AGTGTTGCCGCTCAATAG	157

	RHO-R	GTTGGTTCAGACGGTCAT	
Ribulose-1,5-bisphosphate carboxylase/oxygenase II	RUB-F	ACTCCAATGTCATCCTCAC	159
	RUB-R	CATACTCAATCACGCCATC	
Metacaspase	MET-F	TATTGCTCGTCTCTGGATG	134
	MET-R	CTGTGGTAATGTCAAGATGG	
